# Convergent and selective representations of pain, appetitive processes, aversive processes, and cognitive control in the insula

**DOI:** 10.1038/s41467-026-71568-9

**Published:** 2026-04-14

**Authors:** Mijin Kwon, Ke Bo, Rotem Botvinik-Nezer, Philip A. Kragel, Lukas Van Oudenhove, Tor D. Wager, The Affective Neuroimaging Consortium, Yoni K. Ashar, Lauren Atlas, Lisa Feldman Barrett, Benjamin Becker, Luke Chang, Luana Colloca, Christopher G. Davey, Sigrid Elsenbruch, Miquel A. Fullana, Ben J. Harrison, Olivia K. Harrison, Alec Jamieson, Christian Keysers, Brian Knutson, Leonie Koban, Hedy Kober, Kevin S. LaBar, Claus Lamm, Martin Lindquist, Tina Lonsdorf, Marina Lopez-Sola, Elizabeth Reynolds Losin, Yina Ma, Christian J. Merz, Lauri Nummenmaa, Kyle Pattinson, Pierre Rainville, Marianne Reddan, Rebecca Saxe, Daniela Schiller, Alexander J. Shackman, Dana Small, Carles Soriano-Mas, Rudolf Stark, Choong-Wan Woo, Fadel Zeidan, Feng Zhou

**Affiliations:** 1 Department of Psychological and Brain Sciences, Dartmouth College, Hanover, NH USA; 2 Department of Psychology, The Hebrew University of Jerusalem, Jerusalem, Israel; 3 Department of Psychology, Emory University, Atlanta, GA USA; 4 Department of Psychiatry and Behavioral Sciences, Emory University, Atlanta, GA USA; 5 Laboratory for Brain-Gut Axis Studies (LaBGAS), Translational Research in Gastrointestinal Disorders (TARGID), Department of Chronic Diseases and Metabolism (CHROMETA), University of Leuven, Leuven, Belgium; 6 Division of General Internal Medicine, University of Colorado Anschutz Medical Campus, Aurora, CO USA; 7 National Center for Complementary and Integrative Health, National Institute of Health, Bethesda, MD USA; 8 National Institute of Mental Health, National Institute of Health, Bethesda, MD USA; 9 National Institute on Drug Abuse, National Institute of Health, Baltimore, MD USA; 10 Department of Psychology, College of Science, Northeastern University, Boston, MA USA; 11 Department of Psychiatry and the Athinoula A. Martinos Center for Biomedical Imaging, Massachusetts General Hospital, Boston, MA USA; 12 Department of Psychology, The University of Hong Kong, Hong Kong, China; 13 Department of Pain and Translational Symptom Science, School of Nursing, University of Maryland, Baltimore, MD USA; 14 Department of Psychiatry, The University of Melbourne, Melbourne, Australia; 15 Department of Medical Psychology and Medical Sociology, Center for Medical Psychology and Translational Neurosciences, Ruhr University Bochum, Bochum, Germany; 16 Department of Neurology, Center for Translational and Behavioral Neuroscience (C-TNBS), University Hospital Essen, University of Duisburg-Essen, Essen, Germany; 17 Institut d’Investigacions Biomèdiques August Pi i Sunyer (IDIBAPS), Barcelona, Spain; 18 Adult Psychiatry and Psychology Department, Institute of Neurosciences, Hospital Clinic, Barcelona, Spain; 19 Department of Psychology, University of Otago, Dunedin, New Zealand; 20 Translational Neuromodeling Unit, University of Zurich and ETH Zurich, Zurich, Switzerland; 21 The Netherlands Institute for Neuroscience, KNAW research institute, Amsterdam, The Netherlands; 22 Department of Psychology, University of Amsterdam, Amsterdam, The Netherlands; 23 Department of Psychology, Stanford University, Stanford, CA USA; 24 Lyon Neuroscience Research Center (CRNL), CNRS, INSERM, Université Claude Bernard Lyon 1, Bron, France; 25 Le Vinatier Psychiatrie Universitaire Lyon Métropole, Bron, France; 26 Department of Psychology, University of California Berkeley, Berkeley, CA USA; 27 Department of Psychiatry, Yale University, New Haven, CT USA; 28 Department of Psychology and Neuroscience, Duke University, Durham, NC USA; 29 Department of Cognition, Emotion, and Methods in Psychology, Faculty of Psychology, University of Vienna, Vienna, Austria; 30 Department of Biostatistics, Johns Hopkins Bloomberg School of Public Health, Baltimore, MD USA; 31 Biological Psychology and Cognitive Neuroscience, Bielefeld University, Bielefeld, Germany; 32 Institute for Systems Neuroscience, University Medical Center Hamburg Eppendorf, Hamburg, Germany; 33 Serra Hunter Programme, Department of Medicine, School of Medicine and Health Sciences, University of Barcelona, Barcelona, Spain; 34 Institute of Neuroscience, University of Barcelona, Barcelona, Spain; 35 Institut d’Investigacions Mèdiques August Pi i Sunyer, Barcelona, Spain; 36 Department of Biobehavioral Health, Pennsylvania State University, University Park, PA USA; 37 State Key Laboratory of Cognitive Neuroscience and Learning IDG/McGovern Institute for Brain Research, Beijing Normal University, Beijing, China; 38 Department of Cognitive Psychology, Institute of Cognitive Neuroscience, Faculty of Psychology, Ruhr University Bochum, Bochum, Germany; 39 Turku PET Centre and Turku University Hospital, Turku, Finland; 40 Department of Psychology, University of Turku, Turku, Finland; 41 Nuffield Department of Clin. Neurosciences, University of Oxford, Oxford, UK; 42 Department of Stomatology, Université de Montréal, Montréal, Canada; 43 Research Center of the Montreal Geriatric University Institute, Montréal, Canada; 44 School of Psychology and Neuroscience, Center for Cognitive Neuroimaging, University of Glasgow, Glasgow, UK; 45 Department of Brain and Cognitive Sciences, Massachusetts Institute of Technology, Cambridge, MA USA; 46 McGovern Institute for Brain Research, Massachusetts Institute of Technology, Cambridge, MA USA; 47 Department of Psychiatry, Department of Neuroscience, Icahn School of Medicine, Mount Sinai, New York, NY USA; 48 Friedman Brain Institute, Icahn School of Medicine, Mount Sinai, New York, NY USA; 49 Department of Psychology, University of Maryland, College Park, MD USA; 50 Neuroscience and Cognitive Science Program, University of Maryland, College Park, MD USA; 51 Maryland Neuroimaging Center, University of Maryland, College Park, MD USA; 52 Department of Neurology and Neurosurgery, Department of Medicine, and Department of Psychology, McGill University, Montréal, Canada; 53 Research Institute of the McGill University Health Centre, Montréal, Canada; 54 Modern Diet and Physiology Research Center (MDPRC), Montréal, Canada; 55 Department of Psychiatry, Bellvitge University Hosp., Bellvitge Biomed. Institute-IDIBELL, Barcelona, Spain; 56 CIBERSAM, Madrid, Spain; 57 Department of Social Psychology and Quantitative Psychology, Institute of Neurosciences, University of Barcelona, Barcelona, Spain; 58 Department of Psychotherapy and Systems Neuroscience, Justus Liebig University Giessen, Giessen, Germany; 59 Bender Institute of Neuroimaging, Justus-Liebig-University Giessen, Giessen, Germany; 60 Center of Mind, Brain, and Behavior, Universities of Marburg and Giessen, Giessen, Germany; 61 Center for Neuroscience Imaging Research, Institute for Basic Science, Suwon, Republic of Korea; 62 Department of Biomedical Engineering, Sungkyunkwan University, Suwon, Republic of Korea; 63 Department of Intelligent Precision Healthcare Convergence, Sungkyunkwan University, Suwon, Republic of Korea; 64 Department of Brain Science and Engineering, Sungkyunkwan University, Suwon, Republic of Korea; 65 Department of Anesthesiology, University of California San Diego, La Jolla, CA USA; 66 Faculty of Psychology, Southwest University, Chongqing, China

**Keywords:** Insula, Cognitive neuroscience

## Abstract

Brain regions that integrate multiple types of information (“convergence zones”) are crucial for the brain to generate coherent experiences and behaviors. The insula, known for its functional diversity, has been hypothesized as a key convergence hub, yet empirical evidence remains incomplete. To address this gap, we analyzed functional convergence across four domains—pain, non-somatic appetitive processes, non-somatic aversive processes, and cognitive control—in a Bayesian mega-analysis of fMRI data (*n* = 540, 36 study contrasts). Bayes Factor analyses identified both multi-domain convergent and single-domain selective zones, validated with independent datasets (*n* = 608). Results revealed a hierarchical architecture, with a multi-domain convergence zone in bilateral dorsal anterior insula surrounded by progressively converging zones. Functional decoding and coactivation analyses further support the insula’s role as a convergence hub, while cytoarchitectonic and neurotransmitter profiling characterize the potential neuroanatomical basis of these zones. Together, the findings demonstrate a structured functional topography in the insula that bridges specialized and convergent processing, providing a potential neural basis for combining diverse information streams into unified experiences.

## Abstract (web summary)

Bayesian fMRI mega-analysis reveals structured insular topography, with domain-selective zones with distinct neurobiological properties for pain, appetitive, aversive, and cognitive control processing progressively converging in dorsal anterior insula, providing a potential neural basis for unified subjective experiences.

## Introduction

The human brain possesses an extraordinary ability to integrate a wide array of information from the body and environment into a seamless, unified subjective experience. The insula is thought to be central to this capacity, operating as a convergence zone—a region where diverse information streams are integrated—for a remarkably diverse range of interoceptive and exteroceptive processes, including visceral, autonomic, and homeostatic signals^1,2^, along with information from somatosensory, olfactory, gustatory, and auditory sensory inputs^3–7^.

Most empirical research has focused on identifying subregions specific to particular aspects of sensation, emotion, and cognition, documented by human neuroimaging^1–4,8–23^, pathways in non-human animals^24–27^, and human brain stimulation and electrophysiology^28–33^. In contrast, the concept of functional convergence zones in the insula has been largely theoretical^34,35^.

Convergence zones are thought to exist at multiple levels throughout the brain, from modality-specific integration to higher-order multi-modal convergence^36,37^. Of particular interest are zones that integrate external sensory information with internal states, as this integration is thought to be fundamental for constructing the sense of self^34,38^. An influential theory by Bud Craig^38,39^ posited that the anterior insula (AIns) constitutes a convergence zone contributing to subjective, conscious experience. This aligns with concepts of embodied cognition, where the integration of interoceptive states and sensorimotor capacities underlies cognitive and affective processes^40,41^.

Several lines of evidence support this view. The insula is one of the most functionally diverse regions of the brain^42^, integrating information at long time scales (several seconds or longer^43,44^), and coordinating functional relationships across brain networks^45^. Also, different insular neuronal populations encode diverse interoceptive and special sensory inputs, including visceroception^46,47^, immune afferents^25,48–50^, heartbeat perception^51–53^, pain^54–57^, and taste and smell^58,59^. Von Economo Neurons (VENs) located in AIns might provide a cellular substrate for rapid information integration^60,61^.

Despite the prominence of Craig’s theory, only a few studies have directly evaluated multi-modal convergence zones in AIns, and their precise locations have not been firmly established. Available research has relied on evidence of functional co-localization derived from Coordinate-Based Meta-Analyses (CBMAs)^4,19,20^, including Kurth et al.^4^, which identified partial overlap in AIns across functional domains. While informative, CBMA relies on smoothed peak coordinates with limited spatial precision^62^, which can produce artificial convergence where none exists and cannot systematically evaluate functional specificity across domains or test effect sizes in an unbiased manner.

To provide a comprehensive test of convergent and functionally selective insular zones, we conducted a mega-analysis of participant-level fMRI contrast maps sampled from the Affective Neuroimaging Consortium (www.anic.science) database, focusing on four functional domains: somatic pain, non-somatic appetitive processes, non-somatic aversive processes, and cognitive control^63^. We systematically included three subdomains per domain (e.g., three distinct types of somatic pain), with three studies per subdomain and 15 participants per study (Supplementary Fig. 1; *k* = 36 study contrasts, *n* = 540). This design enabled us to test whether insular subregions encode domain information in a generalizable way across studies, which is critical for construct validation^64^. For example, pain-selective regions should consistently respond to different types of pain (e.g., thermal, mechanical, and visceral pain), but not to non-nociceptive stimuli (e.g., non-somatic, emotionally arousing stimuli). We employed a two-part analytic strategy, examining: (1) global insular activation for domain differences in whole-insula activation, and (2) relative local patterns after z-score normalization to identify regions preferentially engaged by specific domains. We first validated these patterns using cross-validated support vector machines (SVM). Bayes Factors (BFs)^65 that^ provided direct, voxel-wise tests for both the presence and absence of domain-specific activation. This enabled systematic identification of insular subregions that: (1) activate across all domains (“domain-general”), indicating convergence zones, (2) activate selectively to specific functional domains (“domain-selective”), and (3) show gradients of cross-domain convergence. Global analyses revealed that pain produces significantly more widespread activation across the insula compared to other domains. The analysis of relative patterns identified both multi-domain convergence zones in bilateral dorsal anterior insula (dAIns) and domain-selective zones for each domain, which were validated with independent datasets (*n* = 608). Domain-selective inputs, processed separately in distinct insular subregions, progressively converge toward multi-domain zones in bilateral dAIns. To further characterize these zones, we examined meta-analytic functional decoding^19,42^, coactivation with extra-insular brain regions^66,67^, cytoarchitectonic mapping^23,68–72^, and neurotransmitter system profiling^73,74^. Together, these findings provide support for both considerable functional diversity and multi-modal convergence zones in specific parts of the insula, especially in dAIns.

## Results

### Global insular activation across domains

**Fig. 1:**
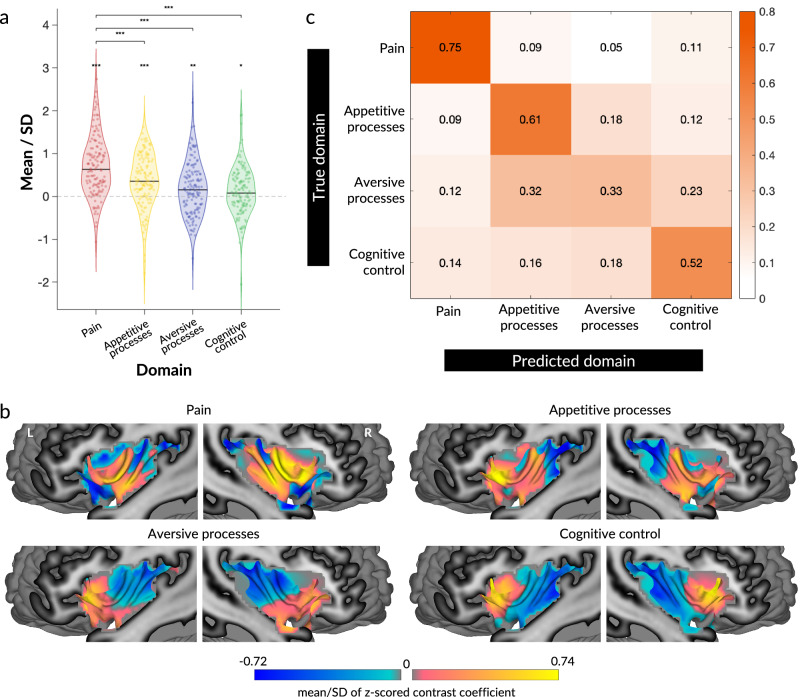
Global insular activation and multi-class SVM classification across four functional domains. **a** Global insular activation across four functional domains. Violin plots show the distribution of whole-insula activation values across participants (points; normalized by study-specific standard deviation). These values are removed when examining relative local patterns. All domains showed significant activation (all FDR-corrected *q* < 0.05). Whole-insula activation was higher for pain than all other domains (all FDR-corrected *q* < 0.001). **b** Participant-level contrast images were z-scored for cross-study harmonization and subsequent analyses. Summary domain activation patterns (voxelwise mean/SD) after z-score normalization are shown here. **c** Prediction accuracy for SVM classifiers trained to discriminate functional domains using leave-one-study-out cross-validation on normalized contrast images. Accurate classification (see text) indicates separable, consistent neural representations for each domain. Source data are provided as a Source Data file.

Analysis of whole-insula activation revealed significant task-related activation compared to within-study controls (i.e., contrast values > 0) for each domain (Fig. 1a; Supplementary Fig. 2): Pain (mean = 0.70*, t*(134) = 11.78, 95% CI [0.58, 0.82], Cohen’s *d* = 1.02); appetitive processes (mean = 0.36, *t*(134) = 6.77, 95% CI [0.26, 0.47], Cohen’s *d* = 0.59); aversive processes (mean = 0.17, *t*(134) = 3.38, 95% CI [0.07, 0.28], Cohen’s *d* = 0.29); and cognitive control (mean = 0.12, *t*(134) = 2.50, 95% CI [0.03, 0.22], Cohen’s *d* = 0.22; all FDR-corrected *q* < 0.05). Pain showed significantly higher whole-insula activation compared to all other domains: versus appetitive processes (*t(268)* = 4.22, 95% CI [0.18, 0.50], Cohen’s *d* = 0.52)*,* aversive processes (*t*(268) = 6.60, 95% CI [0.37, 0.68], Cohen’s *d* = 0.81), and cognitive control (*t*(268) = 7.54, 95% CI [0.43, 0.73], Cohen’s *d* = 0.92; all FDR-corrected *q* < 0.001). These results indicate that pain elicits stronger widespread activations across the insula, consistent with its higher whole-insula activation, possibly due to its engagement of diffuse modulatory systems^75,76^. One consequence is that local posterior insular activity for pain, which was apparent in pre-normalized maps and alternative normalization methods that preserve global insular signals through magnitude scaling without mean centering (see Supplementary Fig. 3), was not significant after normalization.

### Multivariate classification reveals distinct patterns across domains

We next examined relative activation patterns after accounting for the global differences described above (i.e., after normalization; Fig. 1b). We trained multiclass linear SVM classifiers to distinguish each domain from the others based on relative multivariate activation patterns in the insula (see “Methods” for details).

Domain-level classifiers achieved above-chance (>25%) prediction accuracy for three of the four domains: pain (74.71%; range: 40–100%), appetitive processes (60.73%; range: 40–80%), and cognitive control (52.00%; range: 17.78–75.56%; all *p* < 0.001). Aversive processes showed lower accuracy (33.17%; range: 4.44–60%; *p* = 0.1327). The confusion matrix (Fig. 1c) indicated that pain was most discriminable from all other domains. Aversive processes were confusable with appetitive processes, but less confusable with pain and cognitive control (see Supplementary Fig. 4 for pairwise predictive accuracy). Additional subdomain-level classification analyses revealed that some subdomains showed distinct neural patterns (Supplementary Fig. 5), especially responses to aversive sounds (a subtype of aversive processes) and working memory (a subtype of cognitive control; see Methods: Study design for subdomain descriptions).

These findings demonstrate that at the global level, domains differ markedly in their overall insular engagement, with pain showing particularly widespread activation. At the pattern level, pain, appetitive processes, and cognitive control domains show separable insular representations that generalize across studies. These patterns emerge despite substantial methodological variation in stimulus parameters (e.g., stimulus type, dynamics, and durations), task designs, and analytical choices (see Supplementary Table 4).

To localize domain-general and domain-selective patterns within the insula, we applied BF analysis to the normalized images, identifying domain-general and domain-selective zones.

### Domain-general and domain-selective zones identified using Bayes Factors

**Fig. 2:**
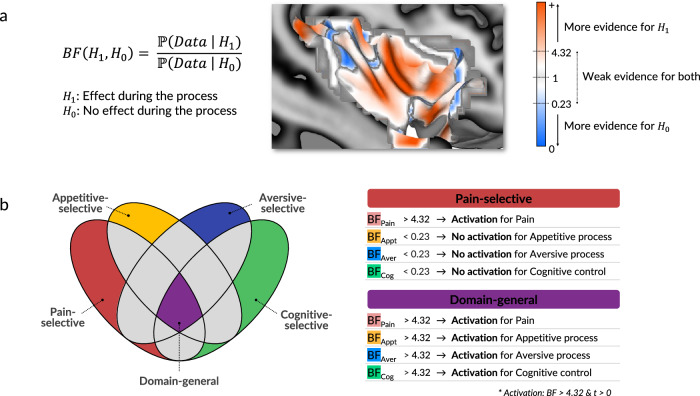
Identifying domain-general and domain-selective insular zones using Bayes Factors. Bayes Factors (BFs) were calculated for each domain. BFs > 1 indicate evidence favoring an effect, while BFs < 1 indicate evidence favoring a null effect. **a** An example map showing voxel-wise BFs for pain (red: favors effect; blue: favors null). Thresholds for sufficient evidence were set at 4.32 for effect (equivalent to FDR-corrected *q* < 0.01) and 0.23 (the inverse of 4.32) for no effect. **b** Definition of domain-general and domain-selective voxels. Voxels were considered activated only when BF > 4.32 and t-statistics were positive, and not activated when BF < 0.23 or BF > 4.32 with negative t-statistics (i.e., significant deactivation). Domain-general voxels were defined as those showing activation in all domains. Domain-selective voxels showed activation in their designated domain and evidence favoring no activation in other domains.

To identify insular zones encoding domain-general or domain-selective responses, we used BFs to assess both presence and absence of domain-specific activation using a Bayesian one-sample t-test^65^ (*n* = 135 per domain). We set a threshold of 4.32:1 odds favoring an effect versus no effect, corresponding to *q* < *0.01* FDR correction on average across domains (Fig. 2a; see “Methods” for details). Voxels were considered activated only when showing both BF > 4.32 and positive t-statistics. Evidence for no activation included cases of either more evidence for no effect (BF < 0.23) or evidence for deactivation (BF > 4.32 with negative t-statistics). Based on these criteria, voxels activated in all four domains independently were classified as domain-general (Fig. 2b, purple), and voxels activated in one domain and showing evidence favoring no activation in the other domains were classified as single domain-selective (Fig. 2b; pain in red, appetitive in yellow, aversive in blue, and cognitive in green). For brevity, we refer to zones selective for each domain as pain-selective, appetitive-selective, aversive-selective, and cognitive-selective throughout the paper. We note that fMRI selectivity at the voxel level should not be interpreted as homogeneous neuronal tuning, as individual voxels likely contain mixed neuronal populations responsive to multiple features.

**Fig. 3:**
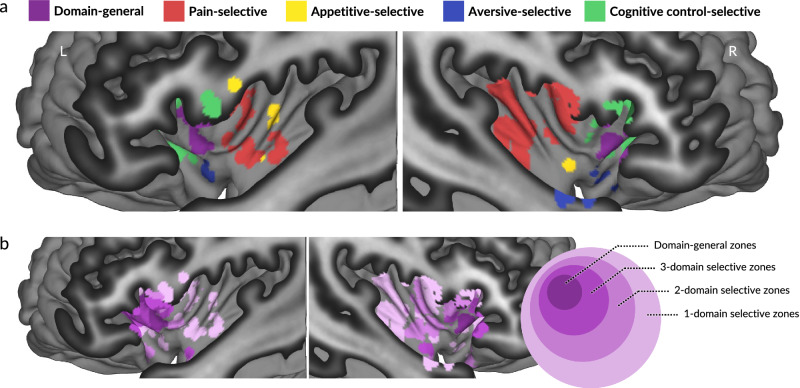
Domain-general and domain-selective zones in the insula. **a** Domain-general voxels (purple) were located in dorsal anterior insula, pain-selective voxels (red) in mid-posterior insula, appetitive-selective voxels (yellow) in mid-insula, aversive-selective voxels (blue) in ventral anterior insula, and cognitive-selective voxels (green) in dorsal anterior insula, anterior and dorsal to domain-general zones. **b** Spatial gradient of functional convergence in the insula. Darker colors indicate greater generalization across domains (from lightest = 1 to darkest = 4 domains) and therefore higher functional convergence. Volumetric data were projected onto insular cutaway surfaces for visualization.

Two specific regions within dAIns bilaterally were activated across all domains and therefore classified as domain-general (Fig. 3a, purple). These zones included 120 of 6012 insular voxels with relatively balanced bilateral distribution and spanned anterior short and inferior gyri^77^. This part of dAIns maps onto regions most frequently activated across task domains in the Neurosynth database^42^ and highest along the principal cortical gradient identified by Margulies et al.^78^, indicating transmodal (as opposed to unimodal) function.

We also identified zones selective to each domain. Pain-selective zones (320 voxels, red in Fig. 3a) included voxels clustered in mid-posterior insula bilaterally, spanning from the middle short to the posterior long gyrus. Appetitive-selective zones (14 voxels, yellow) were proximal to pain-selective zones in left dorsal and bilateral ventral mid-insula, spanning anterior and posterior long gyri. Aversive-selective zones (75 voxels, blue) were primarily located in the ventral-most portion of the bilateral anterior insula, on the anterior inferior gyrus. Cognitive-selective zones (142 voxels, green) were predominantly located in the anterior-most portion of dAIns and most dorsal frontal opercular border bilaterally and spanned anterior and middle short gyri. These activation patterns were consistent across subdomains and studies with few exceptions (Supplementary Fig. 6). Interestingly, these zones displayed distinct patterns of hemispheric asymmetry, with pain and cognitive control showing right hemispheric asymmetry (see Supplementary Table 1 for further information on each insular zone).

We further identified zones activated across two or three domains, surrounding domain-general zones activated across all domains (Fig. 3b). Coupled with the findings above, the topography of these zones demonstrated a spatial gradient of convergence, progressing from domain-selective zones (lightest purple) in posterior and ventral insular areas, through zones responsive to two or three domains, to full multi-domain convergence. This gradient showed bilateral symmetry, revealing a progression from specialized processing to multi-domain convergence.

### Extended functional organization in adjacent opercular regions

While anatomically separable, the insula and operculum often function as an integrated system^38,79,80^ via specific structural connections^81^. We therefore conducted supplementary analyses with an expanded mask including opercular parcels (OP3, 5, 7, and 9) from the Julich-Brain Cytoarchitectonic Atlas^68^ (see Supplementary Methods for details). This revealed similar domain-general and domain-selective patterns in opercular areas with known structural connections to the insula (Supplementary Fig. 7). For example, pain-selective activation appeared primarily in OP5, and domain-general activation was found in OP7 and OP9, consistent with their connectivity to mid-posterior and dorsal anterior insula.

### Validation with independent datasets

The distinct functional zones in the insula that we identified may be limited to the particular studies and task contrasts included. To assess the generalizability of our findings, we validated the identified domain-general and domain-selective zones using 4 independent datasets not included in the primary analysis (total *n* = 608): painful thermal stimulation for pain (*n* = 51)^82^, monetary reward anticipation for appetitive processes (*n* = 32)^83^, aversive image viewing for aversive processes (*n* = 160)^84^, and an n-back working memory task for cognitive control (*n* = 365)^85^ (see “Methods” for detailed task descriptions and contrasts). We examined activation levels within all five functional zones to assess whether domain-selective zones showed preferential responses to their target domains and whether the domain-general zone responded to all domains.

The validation datasets replicated our primary findings regarding global insular activation, with pain showing the highest whole-insula activation (mean/SD = 0.66 ± 0.44), significantly exceeding all other validation datasets (vs. appetitive: *t*(81) = 3.93, *d* = 0.89; aversive: *t*(209) = 3.92, *d* = 0.63; cognitive: *t*(414) = 24.77, *d* = 3.70; all *p* < 0.001).

All domain-selective zones showed significant domain selectivity, with higher activation for the hypothesized domain compared to pooled off-target domains (Supplementary Fig. 8). Pain-selective zones showed the strongest effect (*t*(606) = 6.98, *p* < 0.001, *d* = 1.02), followed by cognitive-selective (*t*(606) = 6.86, *p* < 0.001, *d* = 0.57), aversive-selective (*t*(606) = 4.32, *p* < 0.001, *d* = 0.40), and appetitive-selective zones (*t*(606) = 2.04, *p* = *0*.042, *d* = 0.37). Activation in the domain-general convergence zones was significantly above zero for all test datasets: pain (*t*(50) = 6.01, *p* < 0.001, *d* = 0.84), appetitive processes (*t*(31) = 4.32, *p* < 0.001, *d* = 0.76), aversive processes (*t*(159) = 15.63, *p* < 0.001, *d* = 1.24), and cognitive control (*t*(364) = 22.79, *p* < 0.001, *d* = 1.19). Some studies did show activation to non-target domains, potentially because some tasks can engage psychological processes related to multiple domains. Most notably, the validation task for the appetitive domain (Monetary Incentive Delay task^86^) also engaged cognitive-selective zones, likely because it involves both reward processing and cognitive performance under time pressure that is adaptively adjusted based on feedback.

### Functional decoding using neurosynth topic and term maps

**Fig. 4:**
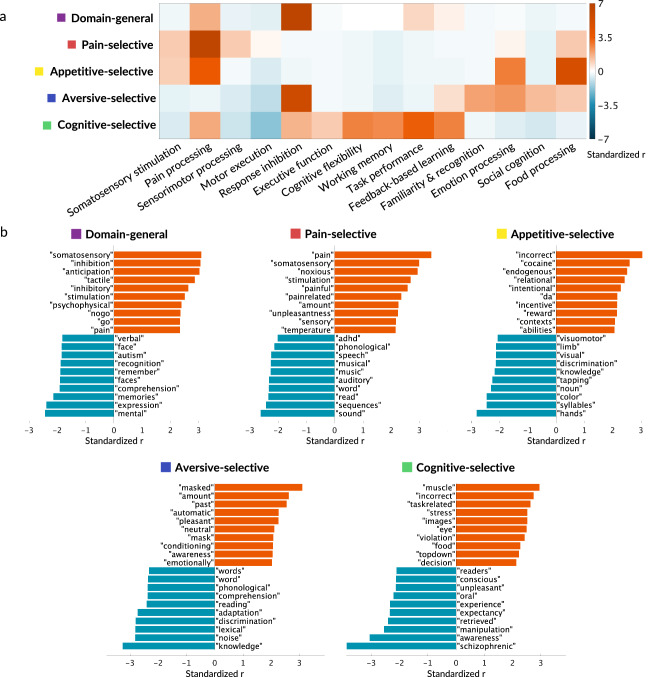
Meta-analytic functional decoding of domain-general and domain-selective insular zones using Neurosynth. **a** Topic-level decoding. Heatmap shows point-biserial correlations between domain-general and domain-selective zones and psychological topics registered in Neurosynth, z-scored across topics (Z(r)). Topics shown (*x*-axis) have Z(r) > 1 for at least one domain (see Supplementary Fig. 9 for complete topic heatmap). **b** Term-level decoding. Each subplot shows the top 10 highest and lowest correlating terms for each insular zone, selected from 525 psychological terms. da Dopamine. Source data are provided as a Source data file.

To map the identified insular zones onto psychological topics, we employed meta-analytic functional decoding using Neurosynth^42^. We examined point-biserial correlations with 525 terms and 50 topics derived from 11,406 studies in the Neurosynth database and z-scoring correlations across topics (Fig. 4a, b; see Supplementary Table 2 for a full list of topics). Domain-general zones in dAIns demonstrated high correlations with diverse topics and terms, reflecting their hypothesized functional diversity. The strongest topic associations were with “response inhibition”, “pain processing”, and “task performance”. The convergence zones ranked in the top 5% of voxels in terms of functional diversity across Neurosynth topic maps (uniformity test maps across 50 topics), showing activation across the most heterogeneous set of topics.

In contrast, domain-selective zones showed associations matching their hypothesized domains. Pain-selective zones exhibited the strongest topic associations with “pain processing”, “somatosensory stimulation,” and “sensorimotor processing” (but also “food processing”). Among individual terms, the strongest were “pain”, “somatosensory”, “noxious”, and “stimulation”. Appetitive-selective zones were associated with “food processing”, “pain processing”, and “emotion processing” topics, and with appetitive processing-specific terms such as “cocaine”, “incentive”, and “reward”. Aversive-selective zones exhibited the strongest associations with topics “response inhibition”, “emotion processing”, “social cognition”, and terms “masked” (indicating priming), “automatic”, “pleasant”, and “conditioning”. Cognitive-selective zones showed the strongest associations with topics “task performance”, “cognitive flexibility”, “feedback-based learning”, and “working memory”, and terms “muscle”, “task-related”, “top-down”, and “decision”, indicating links with top-down control of motion and behaviors.

### Coactivation between insular zones and other brain systems

To better understand how these insular zones participate in brain-wide systems, we investigated their coactivation patterns with other regions across 27,072 contrast maps in the Neurosynth database. This approach provides a meta-analytic proxy of functional connectivity^19,66,67^. We also examined the relationship between each extra-insular coactivated system and seven canonical resting-state networks in cortical, subcortical, and cerebellar regions^87–89^.

**Fig. 5:**
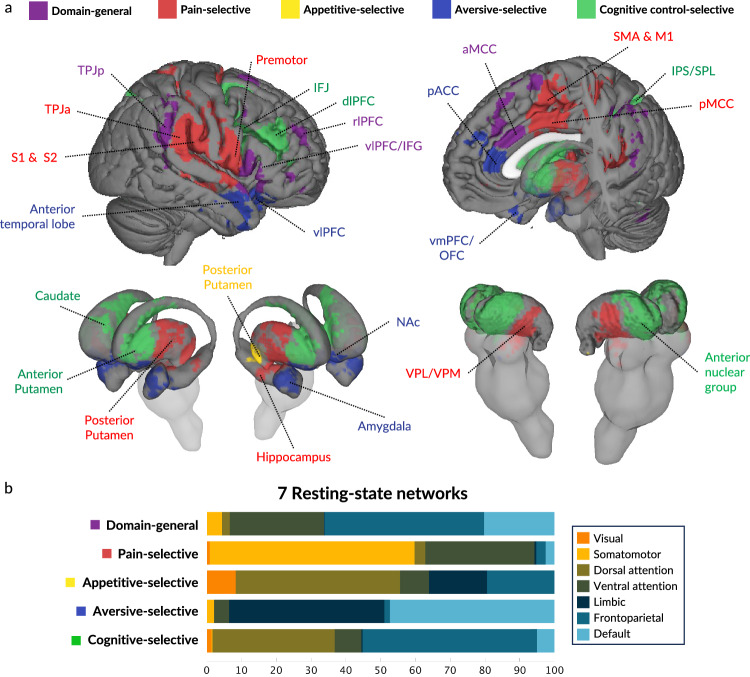
Brain-wide coactivation patterns and resting-state network affiliations of domain-general and domain-selective insular zones. **a** Meta-analytic coactivation patterns between insular zones and extra-insular regions. Coactivated voxels outside of the insula are assigned to the maximally correlated insular zone, requiring that maximum correlation to be at least 10% higher than the next highest correlation. aMCC anterior midcingulate cortex, dlPFC dorsolateral prefrontal cortex, IFJ inferior frontal junction, IPS intraparietal sulcus, SPL superior parietal lobule, M1 primary motor cortex, NAc nucleus accumbens, OFC orbitofrontal cortex, pACC pregenual anterior cingulate cortex, pMCC posterior midcingulate cortex, rlPFC rostrolateral prefrontal cortex, S1 primary somatosensory cortex, S2 secondary somatosensory cortex, SMA supplementary motor area, TPJa anterior temporoparietal junction, TPJp posterior temporoparietal junction, vlPFC ventrolateral prefrontal cortex, vmPFC ventromedial prefrontal cortex, VPL ventral posterolateral nucleus, VPM ventral posteromedial nucleus. **b** Distribution of coactivated regions across seven resting-state networks^87–89^. Bar graph shows the percentage of coactivated voxels (FDR *q* < 0.001) overlapping with each network across cortical, subcortical, and brainstem regions. Source data are provided as a Source data file.

Domain-general zones showed high coactivation with other putative convergence zones across the brain (Fig. 5a), including bilateral rostrolateral (rlPFC), right ventrolateral (vlPFC), and right posterior dorsomedial prefrontal cortices (dmPFC), bilateral posterior temporal parietal junction (TPJp), and bilateral anterior midcingulate cortex (aMCC). No coactivated voxels were found in subcortical regions or the thalamus. These regions lie at the transmodal end of the principal cortical gradient^78^. In relation to the resting-state networks, a majority of voxels coactivating with domain-general zones were located in frontoparietal (45.87%), ventral attention (27.08%), and default (20.30%) networks. These networks are also among the most transmodal networks across the brain.

Pain-selective insular zones showed high coactivation with brain regions involved in pain, somatosensory and somatomotor processing, including bilateral primary and secondary somatosensory cortices (S1 and S2), right premotor and primary (M1) and supplementary motor area (SMA), bilateral posterior operculum, bilateral anterior TPJ (TPJa), and bilateral posterior medial (pMCC) and dorsal posterior cingulate cortex (dPCC). Subcortical coactivation was observed in ventral posterior thalamus, including ventral posteromedial nucleus (VPM), as well as both posterior and anterior putamen, external and internal globus pallidus, subthalamic nucleus in basal ganglia, and the amygdalostriatal transition area and centromedial amygdala. Coactivated voxels were predominantly in somatomotor (58.88%) and ventral attention (31.36%) networks (Fig. 5b). This finding aligns with predictive models of pain^90,91^, where most voxels with positive pain-predictive weights were located in somatomotor and ventral attention networks.

Appetitive-selective insular zones showed high coactivation with the posterior putamen. These coactivated regions, predominantly associated with the dorsal attention network (47.22%), have been implicated in affective processing across multiple meta-analyses^92^ and individual studies^93,94^.

Aversive-selective insular zones showed the highest coactivation with bilateral orbitofrontal cortex (OFC), anterior medial wall—left ventromedial prefrontal cortex (vmPFC), and right posterior dmPFC, bilateral pregenual anterior cingulate cortices (pACC)—bilateral temporal pole, right superficial amygdala, and right nucleus accumbens (NAc). These regions have been broadly associated with the regulation of affective and motivational processes in previous studies. The majority of coactivated voxels were in default mode (47.34%) and limbic (44.65%) networks. This contrasts with somatic pain, which involves different regions and networks despite also being aversive, helping to elucidate why pain and non-somatic negative affect have been strongly dissociable in prior studies^19,93^.

Cognitive-selective zones showed greater coactivation with bilateral dorsolateral PFC (dlPFC), including inferior frontal junction (IFJ), and bilateral intraparietal sulcus (IPS) and superior parietal lobule (SPL). Subcortical regions included thalamus (ventral anterior (VA), ventral lateral (VL), lateral dorsal (LD), mediodorsal (MD), ventromedial (VM) nuclei) and bilateral anterior striatum (putamen and caudate head). These regions have broadly been associated with cognitive control and goal-based action selection. A network including dlPFC, IFJ, and anterior insula has been proposed to select actions to execute via evidence accumulation^95,96^. The mid-caudate zone identified here has also been associated with executive control in a previous meta-analysis^97^. Coactivating voxels were distributed predominantly among frontoparietal (50.14%) and dorsal attention (35.14%) networks.

### Cytoarchitectonic characterization

**Fig. 6:**
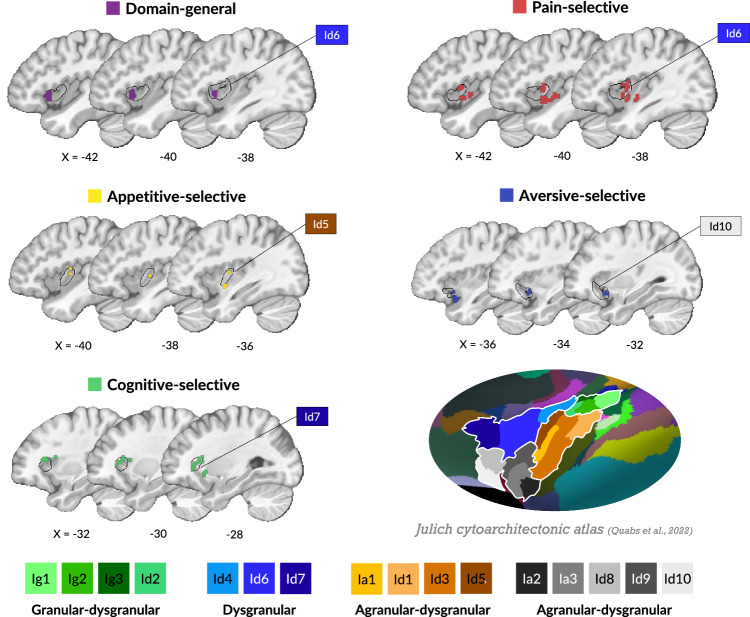
Cytoarchitectonic profiles of insular zones. For each functional zone, only the best matching cytoarchitectonic parcels are shown from the Julich-Brain Cytoarchitectonic Atlas^23,68^ (32 parcels across left and right insula). Inset illustrates the full cytoarchitectonic parcellation of the insula as defined by Quabs et al.^23^ using the most up-to-date atlas.

To explore the cytoarchitectonic associations of the insular zones, we examined their overlap with the Julich-Brain Cytoarchitectonic Atlas^23,68^, which parcellates the insula into 16 areas with varying cytoarchitectonic characteristics: Ig (granular), Id (dysgranular), and Ia (agranular) parcels (Fig. 6). Spatial overlap between each insular zone and atlas parcels was quantified using Dice coefficients, reporting parcels with coefficients > 0.1.

Insular zones mapped onto different cytoarchitectonic regions, though the overlap was imperfect. Domain-general zones were contained predominantly within dysgranular areas, particularly left Id6 and right Id8. Cognitive-selective zones also demonstrated high overlap with bilateral Id7, dorsal and anterior to domain-general zones. In contrast, appetitive and aversive-selective zones were primarily in agranular, dysgranular, or dysgranular-agranular transitional areas: appetitive-selective zones overlapped most with left Id5 and right Ia3, and aversive-selective zones with Id10 bilaterally. Pain-selective zones showed the strongest associations with Id6, but also Id5 and Id3 (in a granular-dysgranular transitional area; see Supplementary Table 3).

### Neurochemical associations with functional insular zones

To characterize associations with neurotransmitter systems derived from human molecular imaging, we estimated spatial associations (point-biserial correlations) between insular zones and PET binding maps for 36 receptors and transporters across 8 neurotransmitter systems from Neuromaps^73,74^. Only associations replicated across at least two independent PET studies were interpreted^98^.

**Fig. 7:**
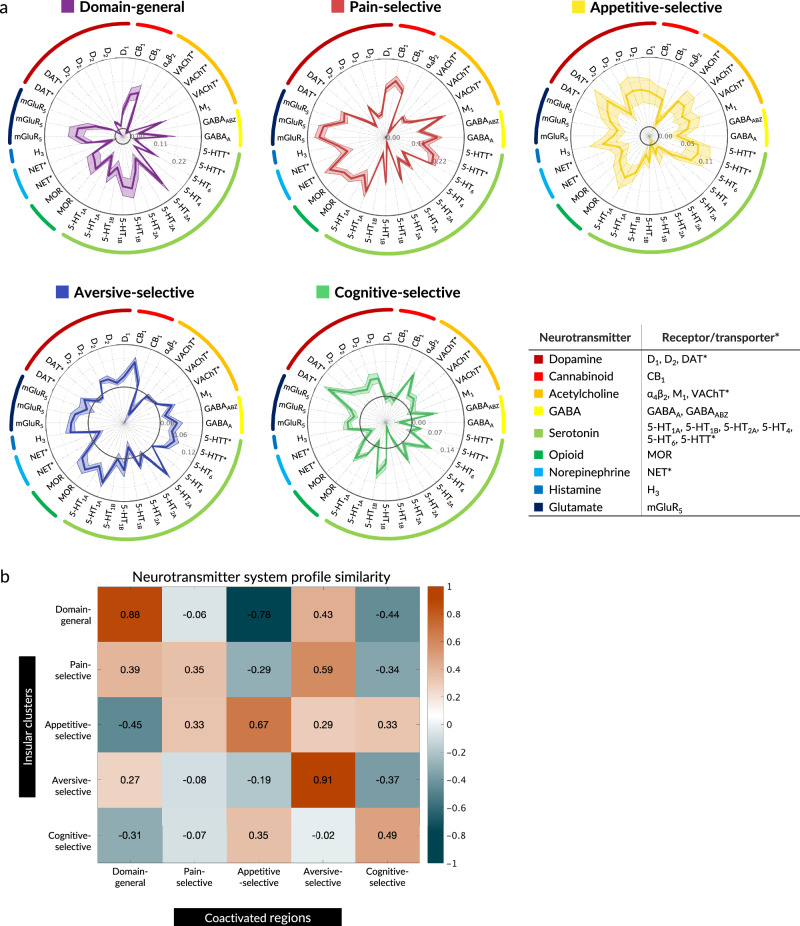
Neurotransmitter system profiles of insular zones. **a** Spatial similarity between the insular zones and 36 neurotransmitter maps from Neuromaps^73,74^ with mean spatial similarity (center) and standard errors (error bars) estimated from 100 bootstrap samples. Black rings in the center of each graph indicate the zero-correlation line; values outside the ring are positive, and inside the ring are negative. Inset shows neurotransmitter systems and their corresponding receptors and transporters in the atlas. **b** Correlation between the insular zones and their extra-insular coactivated systems. Source data are provided as a Source data file.

Several neurotransmitter systems showed reliable associations with particular insular zones (Fig. 7a). Mu-opioid (MOR) and cannabinoid (CB1) receptor maps were positively associated with domain-general zones and all affective functional domains (appetitive, aversive, and pain-selective zones). Affective domains also had particularly strong associations with serotonin receptor 5-HT1a  and transporter 5HTT maps, whereas domain-general and cognitive-selective zones showed relatively stronger associations with serotonin receptor 5-HT1b. Both domain-general and pain-selective zones showed strong correlations with glutamate mGluR5 receptor distributions. Appetitive and cognitive-selective zones shared particularly strong associations with the dopamine D2 receptor distribution. Overall, these findings suggest that different neurotransmitter systems may be differentially related to different functional domains.

Next, we compared neurotransmitter system profiles between the insular zones and their corresponding coactivated brain-wide systems (Fig. 7b). These profiles were highly similar (mean Pearson’s *r* = 0.66; range: 0.35–0.91). For example, positive associations between domain-general insular zones and CB1, 5-HT1b, mGluR5, and MOR were also present in their coactivated brain-wide systems (e.g., anterior PFC, TPJp, aMCC).

Off-target correlations across different functional domains (e.g., between the aversive-selective insular zones and appetitive-selective brain-wide systems) were generally weaker than on-target correlations and were often negative. This correspondence was strongest for domain-general and aversive domains. Domain-general regions showed on-target similarity *r* = 0.88 (off-target range: *r* = −0.78–0.43), and aversive processes showed on-target similarity *r* = 0.91 (off-target range: *r* = −0.37–0.27). Pain showed the least correspondence, suggesting distinct neurotransmitter profiles for pain-selective insular zones compared to their extra-insular coactivated regions. The strongest cross-domain correspondence was that between pain-selective insular zones and aversive-selective coactivated zones, suggesting a possible substrate linking nociceptive representations in the insula and representations of negative emotion in other cerebral areas.

## Discussion

The insula contains subregions associated with diverse cognitive, affective, and interoceptive processes, and its hypothesized role in integrating these streams may be fundamental for unified subjective experiences. However, spatially precise, empirical evidence characterizing functional convergence zones in the insula has been limited. Using large-scale fMRI data (*n* = 540) systematically sampled across four functional domains combined with BFs, we evaluated both presence and absence of activation across domains. We identified a domain-general convergence zone in bilateral dAIns surrounded by domain-selective zones for pain, appetitive processes, aversive processes, and cognitive control. Each showed distinct meta-analytic functional profiles, brain-wide coactivation patterns, cytoarchitectonic organization, and neurotransmitter receptor distributions. We also validated these functional zones using four independent datasets (*n* = 608), confirming both domain-general activation in convergence zones and functional selectivity in domain-selective zones.

We found an orderly progression from domain-selective zones in ventral (negative affect), posterior (pain and appetitive), and dorsal (cognitive) insular regions to the fully domain-general zones in dAIns, extending beyond previously proposed posterior-to-anterior progressions^38,39,99^. These findings support theories proposing hierarchical organization of convergence zones and spatial topography based on functional similarity^36,37^, and align with theories of embodied cognition, emphasizing how the progressive integration of bodily signals enables goal-directed behavior and conscious awareness^34,35^.

Our findings identified insular areas of functional convergence and selectivity with increased precision relative to previous work. The domain-general anterior insula, heuristically defined by Craig^39^ and in early meta-analyses^19^, contains both domain-general and functionally selective zones for cognitive control, somatic pain, and/or non-somatic aversive processes in our analysis. While overlapping BOLD signals alone cannot establish functional convergence, multiple levels of evidence support this interpretation: (a) hierarchical progression from domain-selective to domain-general zones, (b) functional associations based on previous literature, (c) coactivated brain-wide systems, (d) cytoarchitectonic organization, and (e) neurotransmitter system profiles.

Our findings both confirm and extend Kurth et al.’s seminal meta-analysis^4^. While Kurth et al. identified partial overlap across domains in anterior-dorsal insula using coordinate-based methods, our image-based mega-analysis provides greater spatial precision, localizing convergence to specific regions in bilateral dorsal anterior insula. Furthermore, our approach enables us to differentiate domain-general from domain-selective zones and reveals a functional gradient from specialized processing in posterior/ventral regions to multi-domain integration in dorsal anterior insula—a nuanced organization not fully captured in previous work.

The patchy organization we observe aligns with high-resolution tract-tracing studies in non-human primates. Tract-tracing studies show that connections between prefrontal/cingulate cortex and macaque insula consistently form “sharply delimited patches” that precisely coincide with cytoarchitectonic boundaries^100^. This patchy organization, visible across neuroanatomical studies spanning decades^1,101,102^ but previously overlooked, suggests that modular, patchy organization is a fundamental feature of insular architecture rather than statistical noise.

Functional associations using Neurosynth meta-analytic topic maps revealed that domain-general zones in dAIns were among the most functionally diverse (top 5%) across the brain, alongside aMCC, IFJ, and medial thalamus. Meta-analytic coactivation analysis further revealed high coactivation with other cortical convergence zones such as rlPFC, TPJp, and aMCC, which are situated at the transmodal end of unimodal-to-transmodal cortical gradients^78^.

Coactivation patterns across the insular zones showed systematic topographical organization, with organized transitions between functional domains across cortical and subcortical regions. For example, in lateral frontal cortex, we observed an anterior-to-posterior hierarchy from domain-general rlPFC to cognitive control-related dlPFC to pain-related somatomotor regions. This is consistent with findings on prefrontal hierarchy, where anterior regions support more abstract, temporally extended processes and posterior regions support immediate task goals and actions^103–106^. In lateral temporal-parietal cortex, domain-general regions in TPJp were situated between cognitive control-related SPL and pain-related TPJa and S2. Similarly, domain-general regions in IFG were positioned between cognitive control-related premotor regions and anterior temporal regions (connected to medial temporal and hippocampal circuits), processing non-somatic aversive experiences. In the subcortex, a progression emerged from anterior striatal and thalamic zones specialized for cognitive control to posterior zones for pain, with appetitive processing localized to extreme posterior striatum, consistent with stable and long-term value encoding in non-human primates^107^.

With respect to canonical resting-state networks, domain-general and domain-selective insular zones and their coactivated systems showed distinctive distribution patterns. While each domain showed stronger affiliation with some networks (e.g., aversive processes were most affiliated with default and limbic networks), the functional zones we identified spanned multiple networks and did not align with them in a 1:1 fashion. This observation is consistent with findings that decoding models predicting affective experiences span multiple networks^108–111^. Although resting-state networks may relate to task-based patterns at a coarse level^112,113^, tasks evoke reorganization of functional connections in ways not captured by static resting-state patterns^114–116^, including alterations of brain-wide neuronal activity patterns by stimuli and activation of neuromodulatory nuclei^117–121^. This may explain why the insular zones and their coactivated systems identified here are not reducible to resting-state networks, but instead constitute a distinct way of understanding large-scale brain organization.

As with resting-state networks, our functional insular zones mapped onto cytoarchitecture to some degree, but cytoarchitectonic boundaries did not fully describe them. Domain-general convergence zones were located largely within cytoarchitectonic region Id6^23^ in dAIns, but our results also indicate functional heterogeneity within Id6, including pain and cognitive-selective zones in non-overlapping portions of Id6.

The observed cytoarchitectonic mappings were corroborated by evidence from intracranial studies, which provide more direct neural measurements (electrophysiology) and establish causal links to behavior (stimulation). Domain-general and cognitive-selective zones were predominantly located in dysgranular areas Id6 and Id7, respectively, aligning with findings that dysgranular dAIns is key for transmodal processes such as arousal and cognitive control^20,100,122–129^. For example, Sabat et al.^128^ identified a domain-general arousal hub in similar cytoarchitectonic regions to our cross-domain convergence zone, centered in Id7 but extending into Id6 and Id8. Intracranial electrophysiology and stimulation studies showed that Id6 stimulation produced primarily visceral sensations with anxiety/hypervigilance in a small subset of cases, whereas Id7 showed increased activity during task-related salience detection and no responses when stimulated^28^. Aversive-selective zones in Id10 align with known “limbic” projections^127^ and correspond to regions showing specific responses to negative emotional stimuli in electrophysiological recordings^130^.

Pain-selective zones spanned multiple cytoarchitectonic areas in ways that both align with and diverge from previous findings. Beyond Id6’s role in vigilance discussed above, Duong et al.^28^ found that Id6 stimulation evoked visceroception, posterior insular areas Id2 and Ig2 evoked pain, and Id3 evoked somatosensation—consistent with other stimulation findings on somatic and visceral sensations^6,31,57,131,132^. This broad distribution may reflect both the importance of the insula for interoception and the multidimensional nature of pain, which involves a relatively unique combination of regions across the brain^82,133^. However, our findings diverge from established work showing that the posterior granular insula is a key region for somatosensory and nociceptive information^127^. Specifically, we did not observe pain-selective activity in Ig1 (bilateral), Ig2 (left), and Id2 (left) of dorsal posterior insula—regions where previous studies found pain-selective activity^54,55,80,134^ and direct stimulation reliably evokes pain^29,56,126–128,135,136^. While right Ig2 and Id2 showed pain-selective activity, they were not the most strongly associated parcels with our pain-selective zones. This discrepancy likely reflects data harmonization constraints required for analyzing heterogeneous multi-study data: after evaluating several methods (see Methods: Data harmonization), we chose image-wise z-scoring, which eliminates whole-insula activation differences across individuals and domains. This affects the detection of pain-selective responses in the posterior insula, as this region showed pain-selective activity under alternative normalization methods that preserve global insular activation patterns (see Supplementary Fig. 3). Additionally, somatotopic organization and lateralization may have contributed, as our dataset included varied stimulation sites that could obscure somatotopically mapped activity in the posterior insula.

Neurotransmitter receptor and transporter binding patterns from Neuromaps^73,74^ revealed distinct neurochemical profiles across insular zones. Similar patterns were also observed in their coactivated brain-wide systems. This supports the hypothesis that functional organization is guided by co-expression of neurotransmitter systems in functionally related areas, even without direct structural connections^73,137–140^. For instance, language-related areas show similar receptor distributions despite being anatomically dispersed^141^, suggesting a common molecular basis for functionally related regions. Optogenetic and chemogenetic fMRI studies^142–148^ provide corroborating evidence that manipulating neurotransmitter release from subcortical centers modulates functional connectivity among regions with shared receptor profiles.

One exception to the rule of neurotransmitter similarity between functional insular zones and corresponding extra-insular systems was pain. Pain-selective insular zones showed higher neurochemical similarity to the extra-insular aversive processes system than to their own extra-insular pain system. This implies that pain-related neuromodulatory systems may be more region-specific within the insula, or that pain processing in the insula may not be exclusively nociceptive, but also involve pain-specific affective components, i.e., “pain” representations across nociceptive and vicarious pain, as recently observed in mid-insula^111^. This may also reflect the multifaceted nature of pain, which elicits diverse emotional reactions (e.g., fear, anger) and motivates action policies (e.g., avoidance, escape).

These findings highlight the value of systematic multi-domain sampling in revealing both convergent and divergent patterns of insular functional organization and present an important direction for future research. Activity patterns related to broad constructs like “pain” or “cognitive control” can only be effectively identified by testing across distinct subdomains with varied stimuli and task designs^149–151^. While large-sample studies^152–156^ enable investigation of how multiple psychological constructs are represented in the brain across large populations, reliance on single tasks to represent broad psychological constructs (e.g., an emotional face-matching task to study emotion) limits identification of generalizable representations. Characterizing generalizable neural representations requires systematic sampling across multiple implementations of each construct, as demonstrated here and in previous work^157^.

Finally, the present study has several limitations that should be addressed in future research.

First, we selected a Bayes Factor threshold of 4.32:1 (corresponding to *q* < 0.01 FDR correction; moderate evidence by Bayesian standards), balancing sufficient evidence with practical considerations in detecting effects in neuroimaging data. Future studies with larger samples could employ higher thresholds to identify effects with even stronger evidence.

Second, a more comprehensive sampling of each domain would be ideal, and testing the generality and functional specificity in different sets of studies would be valuable as person-level data become available. We have partially addressed this through validation with independent datasets (*n* = 608), which confirmed the robustness of our findings. With sufficient person-level data, future studies could even test studies within subdomains as random effects. Such comprehensive datasets with systematically coded methodological variations would also shed light on how specific methodological variables impact activation patterns. Both advances, however, require systematic sharing of person-level activation maps across the field, extending current practices of reporting study-level coordinates and publishing study-level maps. Our approach here balances comprehensive sampling with practical constraints. With 9 studies per domain across 3 subdomains (540 participants total), our sampling exceeds comparable work^157^ and the key strength lies in systematic sampling across different operationalizations of each domain, enabling identification of patterns that generalize across experimental paradigms. Furthermore, while our study systematically sampled four functional domains, several domains important to insular function remain to be examined—e.g., chemosensation such as smell and taste^58,59^, heartbeat perception^51–53,158^, non-pain somatic aversive experiences like breathlessness^159^ and itch^160^, autonomic arousal^158^, inflammation and sickness^25,48–50^, nausea^161^, tussis^162^, and others. Addressing this will require collaboration and sharing of multiple studies of sufficient size (*n *≥ 15). We view this work as an important step toward understanding generalizable insular representations, while recognizing that future work can extend these findings with additional studies and paradigms.

Third, our psychological domain structure represents just one possible ontology for classifying studies^163–166^. The development and validation of ontologies is a complex and important research topic, and there is no single “correct” solution. Future research with extended samples could empirically compare different candidate ontologies, working toward developing psychological categories better aligned with the functional architecture of the brain^165,167^. Our validation findings underscore this need: while confirming domain selectivity, they also revealed that tasks can activate multiple functional zones based on their component processes. The appetitive task^83^ in our validation dataset elicited activation in both appetitive- and cognitive-selective zones, consistent with the task’s cognitive demands—maintaining stimulus-reward mappings and sustaining inhibitory control during anticipatory delays—in addition to reward anticipation. This also highlights the iterative nature of brain-behavior mapping: task categorization influences identification of neural patterns, while neural results inform understanding of task components.

Fourth, we acknowledge that the insula exhibits considerable inter-individual variability in sulcal and gyral morphology. Our analyses identify group-level organization using template-based normalization to MNI space, mapping function onto anatomical features (gyri and sulci) at the group level. Future studies using precision neuroimaging approaches with within-person tasks could examine how anatomical variability influences functional localization and how closely domain-general and domain-selective regions map onto individual anatomical features.

Fifth, our cytoarchitectonic mapping relied on maximum probability maps from the Julich-Brain atlas, where each voxel is assigned to the area with the highest probability. This approach simplifies the inherent uncertainty in these assignments, particularly at borders between areas. Future studies could address this by using probabilistic approaches that preserve uncertainty information or by examining structure-function relationships at the individual subject level. We hope our study inspires future work to expand this approach and address these outstanding long-term goals.

Lastly, our analysis focuses primarily on activation patterns and does not extensively consider information carried by deactivation. While aggregating individual-level contrast images allows for observation of deactivation patterns that can provide valuable insights, interpreting these patterns presents significant challenges, as their underlying mechanisms are less well understood than those of activations. The relationship between BOLD signal decreases and neural activity may vary across brain regions, involving interactions between neural activity, neurovascular coupling, and hemodynamics^168–170^. Beyond this regional variability, BOLD decreases can reflect various neural processes, from inhibition of task-irrelevant activity to resource reallocation or shifts between functional modes^171,172^. Additionally, the choice of baseline condition significantly affects observed deactivation patterns, complicating cross-study comparisons. Given these complexities and the activation-based nature of our subsequent multi-level characterization analyses, we focused our primary Bayes Factor analysis on activation patterns. However, recognizing the potential importance of deactivation, we have included deactivation-based results in Supplementary Fig. 10 for reference. We encourage future studies to further investigate deactivation patterns for various brain functions in and beyond the insula.

Overall, this study provides strong empirical evidence for a fundamental organizing principle in the insula: the coexistence of functionally convergent and selective zones within a brain region long hypothesized to be crucial in integrating diverse information streams. Through systematic sampling across four functional domains and rigorous Bayes Factor analysis of both presence and absence of activation, we reveal a gradient of convergence from domain-selective zones to a multi-domain convergence zone in bilateral dAIns, combining spatial coverage, precision, and functional diversity beyond previous approaches. Our multi-level characterization demonstrates that these functional zones are distinguished by specific patterns of cytoarchitecture, coactivation with other brain regions, and neurotransmitter distribution, providing evidence beyond mere overlap of BOLD signals that these represent distinct functional units. These results reconcile previous work by demonstrating how functional specialization and information convergence coexist within the insula’s hierarchical organization, suggesting a potential role for this architecture in integrating diverse information streams to support unified subjective experiences and the construction of the sense of self.

## Methods

### Study design

This study uses a construct-validation approach, building upon previous research by Kragel et al.^157^ and Van Oudenhove et al.^173^, to explore domain-general and domain-selective representations of four functional domains in the insula: somatic pain, non-somatic appetitive processes, non-somatic aversive processes, and cognitive control. This approach aims to identify brain regions that consistently respond to a particular psychological construct (e.g., pain) across multiple experimental manipulations and studies, ensuring that the voxels identified as domain-general and domain-selective are not driven by specific experimental conditions or study-specific factors, but rather represent the underlying latent construct (for further details on the rationale of this approach, see refs. ^157,173^).

Specifically, we systematically sampled participant-level fMRI activation maps from the Affective Neuroimaging Consortium (www.anic.science) database across the four domains above. Each domain includes three subdomains representing different experimental manipulations that engage processes within that domain. Pain domain includes responses to thermal, mechanical, and visceral stimulation; appetitive processes domain includes responses to food, drug, and sexual images; aversive processes domain includes responses to negative images, aversive sounds, and negative social interactions; and cognitive control domain includes responses during working memory, response inhibition, and attention switching tasks. For each subdomain, we included three independent studies using similar experimental manipulations, with 15 randomly sampled participants per study (total *k* = 36 study contrasts, *n* = 540). This hierarchical and balanced design allows us to dissociate insula sub-areas that are selective for a specific domain and those that generalize across all four domains. See Supplementary Table 4 for a full list of studies included in the current dataset.

The current study was a meta-analysis of multiple independent studies. Participants were recruited independently for each study and informed consent was provided by all subjects in accordance with local ethics and institutional review boards. Descriptions of ethics approvals, image acquisition, and demographics are described briefly for all studies in Supplementary Table 4 and in full detail in the corresponding references (see also the Life Sciences Reporting Summary).

### Data harmonization

To address potential differences in data scaling across studies, we employed a two-part analytic strategy capturing both global and local functional properties: (1) we analyze global insular activation to identify domain differences in overall engagement using mean activation values, and (2) we analyze relative local patterns after normalization to identify regions preferentially engaged by specific domains. This dual approach is necessary because some domains may produce widespread activation that could mask more subtle selective patterns in other domains.

To implement this strategy, we first resampled all data into a reference space (Study 1^174^) and analyzed the mean activation values normalized by each study’s average voxelwise between-subject SD to assess differences in whole-insula activation between domains, preserving information about global activation patterns before z-score normalization.

Next, we applied within-subject z-score normalization across voxels within the insula to examine relative local patterns. By analyzing patterns of values across voxels instead of absolute intensities, this approach accounts for idiosyncrasies in the scale of activity across studies, influenced by factors including differences in acquisition, preprocessing, voxel size, contrast weights, and other nuisance factors. This implies that local activity estimates are lower in images with more overall activations across the insula, leading to more conservative results, as domain convergence and selectivity analyses are performed on the relative activation maps.

We chose z-scoring after considering several alternative harmonization methods including ComBat^175^, L2 normalization, and standard deviation normalization. These alternatives were either not applicable due to the nested relationship between scanning parameters and tasks, or did not significantly improve harmonization (see Supplementary Methods for detailed discussion of harmonization approaches and their limitations). Similar normalization procedures are common in multivariate pattern analysis in fMRI and in machine learning and multivariate statistics more broadly (e.g., in profile analysis)^176–178^. Here, it allows us to focus on relative differences in brain patterns across the four mental constructs while minimizing the impact of study-specific factors.

### Multiclass support vector machine classifier

We trained multi-class linear SVM classifiers with 5-fold cross-validation for hyperparameter optimization (box constraint). The classifiers were trained to discriminate each domain from the others (one vs. all scheme) using two studies per subdomain for training and a third study as an independent test set for domain and subdomain-level evaluations (e.g., pain from all other domains, thermal pain from all other subdomains). To estimate out-of-study generalization error, we systematically sampled different studies for training and test sets by leaving a different study out in each subdomain for each model, training 100 models in total with splits stratified by subdomain, and reported the average performance on the test sets (see Supplementary Fig. 11 for a schematic description of study selection).

### Bayes factors

We calculated Bayes Factors (BFs) at the voxel level using Bayes Factor one-sample t-test with the Jeffrey-Zellner-Siow prior (JZS, Cauchy distribution on effect size; ref. ^65^) to identify voxels in the insula exhibiting domain-general or domain-selective activation patterns. For each domain, we aggregated data across all three subdomains, yielding *n* = 135 samples per domain (3 subdomains × 3 studies × 15 participants). BF directly compares the probability of the data under two competing hypotheses: the alternative hypothesis (an effect exists) versus the null hypothesis (no effect).

First, the Bayes Factor for a one-sample t-test (with *n* = 135 per domain, aggregating across 3 subdomains × 3 studies × 15 participants) is calculated as follows (Eq. 1):

1 $${B}_{01}=\frac{{\left(1+\frac{{t}^{2}}{\nu }\right)}^{-(\nu+1)/2}}{{\int }_{0}^{\infty }{(1+{Ng}{r}^{2})}^{-1/2}{\left(1+\frac{{t}^{2}}{\nu (1+{Ng}{r}^{2})}\right)\!}^{-(\nu+1)/2}{(2\pi )}^{-1/2}\,{g}^{-3/2}\,{e}^{-1/(2g)}{dg}}$$

where $t$ is the t-statistic, $N$ is the sample size, $\nu$ is the degrees of freedom ($N-1$), $g$ is a prior parameter on effect size that is integrated out under the JZS prior, and $r$ is the scale factor (set to 0.707, indicating a moderate expected effect size; refs. ^65,98^).

We set thresholds at BF > 4.32 for an effect and BF < 0.23 (an inverse of 4.32) for no effect, corresponding to 4.32:1 odds. This decision was supported by previous literature suggesting that Bayes Factors with JZS between 3 and 10 are considered moderate-level evidence^179–181^. These thresholds were chosen to ensure the identification of effects and null effects, minimizing the potential for false positives while maintaining sensitivity to true effects.

Voxels were considered activated only when showing both BF > 4.32 and positive t-statistics. The spatial extent and configuration of functional zones remained stable across a range of thresholds (Supplementary Fig. 12). Evidence for no activation included cases of either evidence for no effect (BF < 0.23 with either positive or negative t-statistics) or evidence for a deactivation effect (BF > 4.32 with negative t-statistics). By conjunction of BFs across all domains, domain-general voxels required activation across all domains, while domain-selective voxels required activation in their designated domain and evidence for no activation in other domains. Specifically, domain-general voxels were defined as those with BF > 4.32 and *t* > 0 across all domains. Domain-selective voxels were defined as those with (1) BF > 4.32 and *t* > 0 for the designated domain, and (2) BF < 0.23 or BF > 4.32 and *t* < 0 for the other domains.

### Anatomical definition of insula

For our primary analyses, we defined the insula based on established anatomical landmarks, including the three short gyri of the anterior insula (anterior, middle, and posterior short gyri), the two long gyri of the posterior insula (anterior and posterior long gyri), and the anterior inferior cortex, bounded by the peri-insula sulcus that separates the insula from surrounding opercula. Given ongoing debates about insular boundaries and functional connectivity with adjacent opercular regions, we conducted supplementary analyses using an expanded anatomical definition. This expanded mask included the insula proper plus adjacent opercular regions (OP3, OP5, OP7, and OP9) from 2 different atlases: HCP-MMP1.0^182^ from the Human Connectome Project (HCP)^153^ and Julich-Brain Cytoarchitectonic Atlas^23,68^. This tested whether our main findings depend on specific anatomical definitions (see Supplementary Methods and Supplementary Fig. 7).

### Validation with independent datasets

To validate the identified functional zones, we tested activations in each zone using independent validation datasets selected from the ANiC database (after our primary dataset was finalized) and from open data repositories (e.g., Human Connectome Project (HCP)^153^) when domain-specific data were unavailable in the database. We selected one study per domain known to robustly and selectively engage that domain: thermal pain stimulation for pain (*n* = 51; high pain)^82^, Monetary Incentive Delay task for appetitive processes (*n* = 32; monetary reward anticipation)^83^, viewing of negative images for aversive processes (*n* = 160; viewing of negative images selected from the International Affective Picture System (IAPS))^84^, and n-back working memory task from the HCP dataset for cognitive control (*n* = 365, restricted to genetically unrelated participants; 2-back (place))^85^. See Supplementary Table 5 for detailed study information.

Validation data underwent identical quality control procedures and preprocessing pipelines as the primary datasets. We analyzed global activation patterns by comparing the mean/SD values across domains to test whether domain differences in overall insular activation replicated. We then extracted mean activation values from the five functional zones identified in our primary analysis (one domain-general zone and four domain-selective zones). For domain-selective zones, we assessed selectivity by comparing activation in the target domain against pooled off-target domains. For domain-general zones, we tested whether each domain showed significant activation above zero.

### Neurosynth functional decoding

We examined spatial correlations between the identified insular zones and two types of meta-analytic maps: 525-term and 50-topic association test maps that show voxels preferentially associated with studies related to particular psychological terms or topics. Topic maps were streamlined from a set of 100 topics (“vs-topics−100”) that were extracted using Latent Dirichlet Allocation (LDA) from abstracts of all articles in the Neurosynth database as of July, 2015 (11,406 articles), after excluding non-psychologically relevant topics. We used standardized point-biserial correlations to calculate spatial correlations between our identified insular zones and these two types of meta-analytic maps for topics and terms. Correlation coefficients were z-scored within each zone to examine the relative strength of correlations across topics for each zone. We report topics showing standardized correlation > 1 and the top 10 highest and lowest correlating terms for each insular zone. See Supplementary Table 2 for a full list of Neurosynth topic maps used in the current analysis.

### Neurosynth functional diversity analysis

To assess functional diversity across brain regions, we used the uniformity test maps from the same set of 50 topics, which show voxels consistently activated across studies associated with each topic. For each brain voxel, we calculated the proportion of topics showing significant activation. Voxels were then ranked brain-wide based on this proportion to quantify their functional diversity across psychological topics.

### Coactivation between the insula and other brain areas

We performed a meta-analytic coactivation analysis using activation coordinates from 27,072 studies registered in the Neurosynth repository as of April, 2022. We created meta-analytic binary activation maps using Multi-level kernel density Analysis (MKDA; ref. ^183^) with a 4-mm smoothing kernel. In these maps, a voxel was coded as active for a study if the reported peak coordinate was within 4-mm of the given voxel. Using these meta-analytic activation maps, we calculated point-biserial correlations between each domain-general and domain-selective insular zone and each voxel in the cortical and subcortical regions of the whole brain, excluding the insula. The resulting correlations were thresholded at FDR-corrected *q* < 0.001 and cluster size > 100 voxels. Voxels above the threshold were assigned to one of the insular zones based on which zone showed the maximum correlation, provided that the maximum correlation with one of the insular zones was at least 10% higher than the next highest correlation.

To examine the relationship between the coactivated regions and seven resting-state networks (combined from refs. ^87–89^), we calculated the percentage of voxels in the thresholded coactivated regions that overlapped with each of the seven resting-state networks in the cortical, subcortical, and cerebellar regions. A supplementary analysis using unthresholded coactivation maps is provided in Supplementary Fig. 13.

### Cytoarchitectonic profiling

To determine the best matching cytoarchitectonic parcel for each insular zone, we calculated spatial overlap between each insular zone and each atlas parcel from the Julich-Brain Cytoarchitectonic Atlas^23,68^ (see Fig. 6) using the Dice Coefficient (DC), a measure ranging from 0 (no intersection) to 1 (complete intersection). This atlas includes 16 distinct parcels in the insula based on their common cytoarchitectonic characteristics (granular, dysgranular, and agranular). We selected parcels with a DC higher than 0.1 to report and note which group each insular zone is predominantly associated with.

### Neurotransmitter system profiling

We used a comprehensive receptor and transporter binding map atlas (Neuromaps^73,74^) that contains 40 PET-derived binding maps covering 19 neurotransmitter receptors and transporters and 9 systems in over 1200 healthy individuals. The atlas includes maps for the following receptors and transporters (transporters indicated with*): α4β2, M1, VAChT* (acetylcholine); CB1 (cannabinoid); D1, D2, D3, DAT* (dopamine); GABAA/BZ (GABA); mGluR5, NMDA (glutamate); H3 (histamine); NET (norepinephrine); MOR (opioid); 5-HT1a, 5-HT1b, 5-HT2a, 5-HT4, 5-HT6, 5-HTT* (serotonin). Here, we used 36 images after removing 4 images due to issues found during implementation.

We calculated point-biserial correlations between each insular zone and each receptor and transporter map in the atlas. Higher correlations indicate the relatively greater density of a given neurotransmitter receptor/transporter. To evaluate the significance and error variability of associations, we bootstrapped each domain-general and selective insular map for 100 iterations. For each iteration, we resampled individual contrast maps and regenerated four insular maps for each domain based on these bootstrap samples. Spatial correlations were then calculated between each bootstrapped insular map and each PET binding map, reporting the mean and standard deviation of these spatial correlations across the 100 bootstrap samples as an estimate of the standard error. For the representativeness of the results, we only interpret the results where (1) there is more than one study on the same neurotransmitter receptor or transporter, and (2) the results from all studies on the same neurotransmitter receptor or transporter are in agreement. We made exceptions for cases showing meaningful associations based on previous literature. We applied the same approach to the coactivated extra-insular regions of each insular zone. For each insular zone, we computed Pearson’s correlations between its neurotransmitter receptor profile (pattern of receptor densities across different systems) and the receptor profile of its corresponding coactivated regions to test for shared patterns of receptor/transporter distributions.

### Reporting summary

Further information on research design is available in the Nature Portfolio Reporting Summary linked to this article.

## Supplementary information

Supplementary information

Reporting Summary

Transparent Peer Review file

## Source data

Source Data

## Data Availability

The fMRI data from the main-analysis studies 1, 2, 4, 5, 7, 8, 19, 20, 22, 23, 25, 26, 28, 29, 31, and 32 are available at 10.6084/m9.figshare.24033402.v2. Data from studies 3 and 6 are available at https://neurovault.org/collections/8707/. Data from the validation dataset for cognitive control (n-back working memory task) are available from the Human Connectome Project database. The remaining datasets are available upon request from the corresponding authors of the individual studies. The Neurosynth dataset is available at https://github.com/canlab/Neuroimaging_Pattern_Masks/tree/master/neurosynth, the cytoarchitecture maps at https://github.com/canlab/Neuroimaging_Pattern_Masks/tree/master/Atlases_and_parcellations/2020_JulichBrain_v3.0.3, and the neurotransmitter receptor/transporter maps at https://github.com/canlab/Neuroimaging_Pattern_Masks/tree/master/Atlases_and_parcellations/2022_Hansen_PET_tracer_maps. Source data are provided with this paper.

## Code availability

Matlab code for implementing all analyses is available at https://github.com/canlab/ and https://github.com/mijinjkwon/proj_insula_anic.
