# Supplementary material for: Convergent and selective representations of pain, appetitive processes, aversive processes, and cognitive control in the insula
Source: Nat Commun. 2026 Apr 14;17:5186. doi: 10.1038/s41467-026-71568-9 (PMC13254212; doi:10.1038/s41467-026-71568-9)
Supplement: Supplementary file 1 — Supplementary information [file 41467_2026_71568_MOESM1_ESM.pdf]

Supplementary Information

Convergent and selective representations of pain, appetitive processes, aversive processes, and cognitive control in the insula

Mijin Kwon[1*], Ke Bo[1], Rotem Botvinik-Nezer[1,2], Philip A. Kragel[3,4], Lukas Van Oudenhove[5], Tor D. Wager[1*], The Affective Neuroimaging Consortium[6]

[1] Department of Psychological and Brain Sciences, Dartmouth College, Hanover, NH, United States

[2] Department of Psychology, The Hebrew University of Jerusalem, Jerusalem, Israel

[3] Department of Psychology, Emory University, Atlanta, GA, United States

[4] Department of Psychiatry and Behavioral Sciences, Emory University, Atlanta, GA, United States

[5] Laboratory for Brain-Gut Axis Studies (LaBGAS), Translational Research in Gastrointestinal Disorders (TARGID), Department of Chronic Diseases and Metabolism (CHROMETA), University of Leuven, Leuven, Belgium

[6] Group author

* Corresponding authors

Correspondence to:

Tor D. Wager
Diana L. Taylor Distinguished Professor
Presidential Cluster in Neuroscience and
Department of Psychological and Brain Sciences
Dartmouth College
Email: tor.d.wager@dartmouth.edu

Mijin Kwon
Department of Psychological and Brain Sciences
Dartmouth College
Email: mijin.kwon.gr@dartmouth.edu

Supplementary Methods

Data harmonization and normalization

When analyzing data aggregated from multiple studies across various functional domains, it is crucial to account for two types of variability or biases to accurately identify functionally convergent and selective areas in the insula. Here we refer to these two as 1) study-wide variability and 2) functional domain-wide variability. Study-wide variability is primarily a technical issue, while domain-wide variability reflects both biological and technical/methodological differences between domains.

Study-wide variability: Data from different studies and sites are inevitably measured on different scales due to variable factors during data acquisition and analysis. These factors include scanner, acquisition, and analysis variables, such as field strength, TR, TE, acquired voxel size, flip angle, choice of baseline state, stimulus timing, physiological noise removal and filtering choices, scaling of the hemodynamic response function(s) used, model regressors and contrast weights, choice to analyze percent signal change, method for converting to percent signal change, choice to resample voxels, and contrast scaling across multiple runs.

Domain-wide variability: The magnitude of brain activity associated with each functional domain differs. For example, in our dataset, painful stimuli generate more pronounced (higher activation) and widespread activity across the brain, including the insula, compared to the other domains we examined. These differences reflect both biological factors (e.g., pain's engagement of arousal and salience systems) and technical/methodological factors (e.g., differences in task design, stimulus parameters, and implementation across domains). While the biological differences are meaningful signals reflecting how domains genuinely differ in their neural engagement, they create analytical challenges when identifying domain-selective regions. Without proper data harmonization to account for these differences in signal levels, it would be challenging to identify functionally selective areas for other domains.

Consideration of other data harmonization and normalization methods

To address these issues, we explored a range of data harmonization and normalization methods, including ComBat[1], L2 normalization, normalization with standard deviation as a norm, and z-scoring. ComBat has been successfully applied in various neuroimaging modalities, such as DTI[2], cortical thickness[3], volumetric T1[4], functional connectivity[5,6], and task-based fMRI[6]. However, ComBat was not applicable in our case due to the nested relationship between sites/scanning parameters and task/study. L2 normalization and normalization with standard deviation as a norm did not significantly improve data harmonization compared to raw data. Therefore, we decided to analyze relative patterns after applying z-scoring normalization at the image level across voxels to account for site, scanner, and inter-study variability while still finding meaningful differences in activation patterns within the insula between the included functional domains. Although this approach may result in the loss of some informative signals, such as pain-related activity in the posterior insula, it enables the identification of local coding patterns for each domain.

Supplementary analysis with expanded insula boundaries

Given the close functional connectivity between insular cortex and adjacent opercular regions, we tested whether our findings depend on specific anatomical boundary definitions. We repeated our main Bayes Factor analysis to identify domain-general and domain-selective zones with an expanded mask that included both insula proper and adjacent opercular regions. The expanded mask was constructed using parcels from two complementary atlases: HCP-MMP1.0[7] and Julich-Brain Cytoarchitectonic Atlas[8]. For the Julich-Brain Atlas, we selected cytoarchitectonically-defined opercular areas OP3, OP5, OP7, and OP9. For HCP-MMP1.0, since this atlas does not contain the same opercular parcels, we identified anatomically corresponding regions based on spatial proximity. The following parcels were selected from each atlas:

1. **Julich-Brain Cytoarchitectonic Atlas**:
    - Insular parcels: Ig1, Ig2, Ig3, Id1, Id2, Id3, Id4, Id6, Id7, Id8, Id9, Id10, Ia1, Ia2 (bilateral)
    - Opercular parcels: OP3, OP5, OP7, OP9 (bilateral)
2. **HCP-MMP1.0 Atlas**:
    - Insular parcels: 52, PI, Ig, PoI1, PoI2, MI, AVI, AAIC, Pir (bilateral)
    - Opercular parcels: FOP2, FOP3, FOP4, FOP5 (bilateral)

Results from this expanded analysis are presented in Supplementary Fig. 7. We reported results only from the analysis using the parcels from the Julich-Brain Cytoarchitectonic Atlas in the main results, but Supplementary Fig. 7 includes results from both atlases for comparison, demonstrating consistent findings across different parcellation schemes.

Supplementary Figures

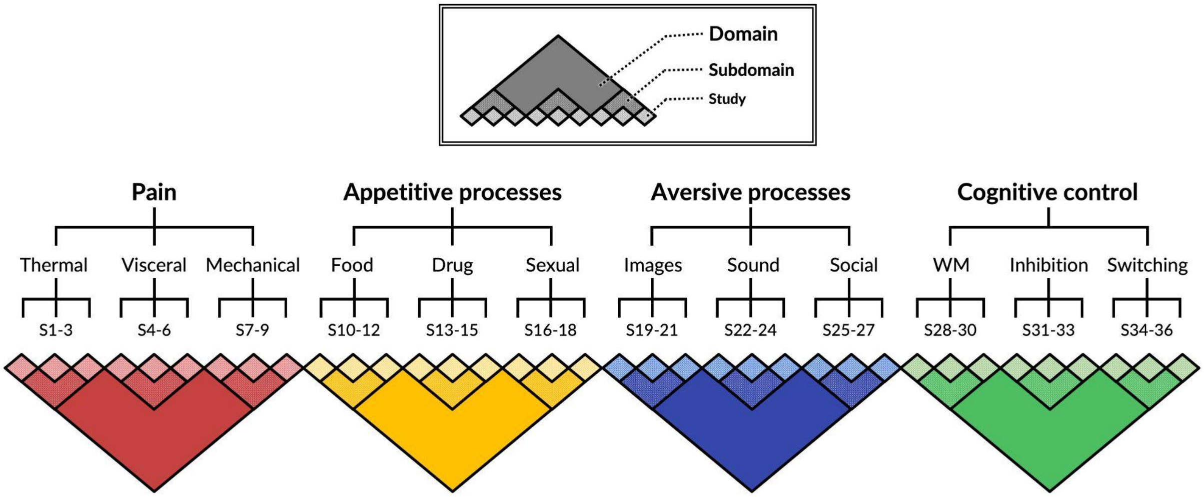

Supplementary Fig. 1

Multi-study data structure. Dataset used in the current study consists of 15 participants from each of 36 fMRI studies systematically sampled across four functional domains closely linked to insular function: somatic pain, non-somatic appetitive processes, non-somatic aversive processes, and cognitive control. Each functional domain comprises three subdomains representing different experimental paradigms within that domain, with three studies per subdomain to ensure representativeness and generalizability. Pain domain includes thermal (thermal stimulation), mechanical (mechanical stimulation), and visceral (visceral stimulation) subdomains. Appetitive processes domain includes food (food images), drug (drug images), and sexual (sexual images) subdomains. Aversive processes domain includes images (negative images), sound (aversive sounds), and social (negative social interactions) subdomains. Cognitive control domain includes WM (working memory), inhibition (response inhibition), and switching (attention switching) subdomains.

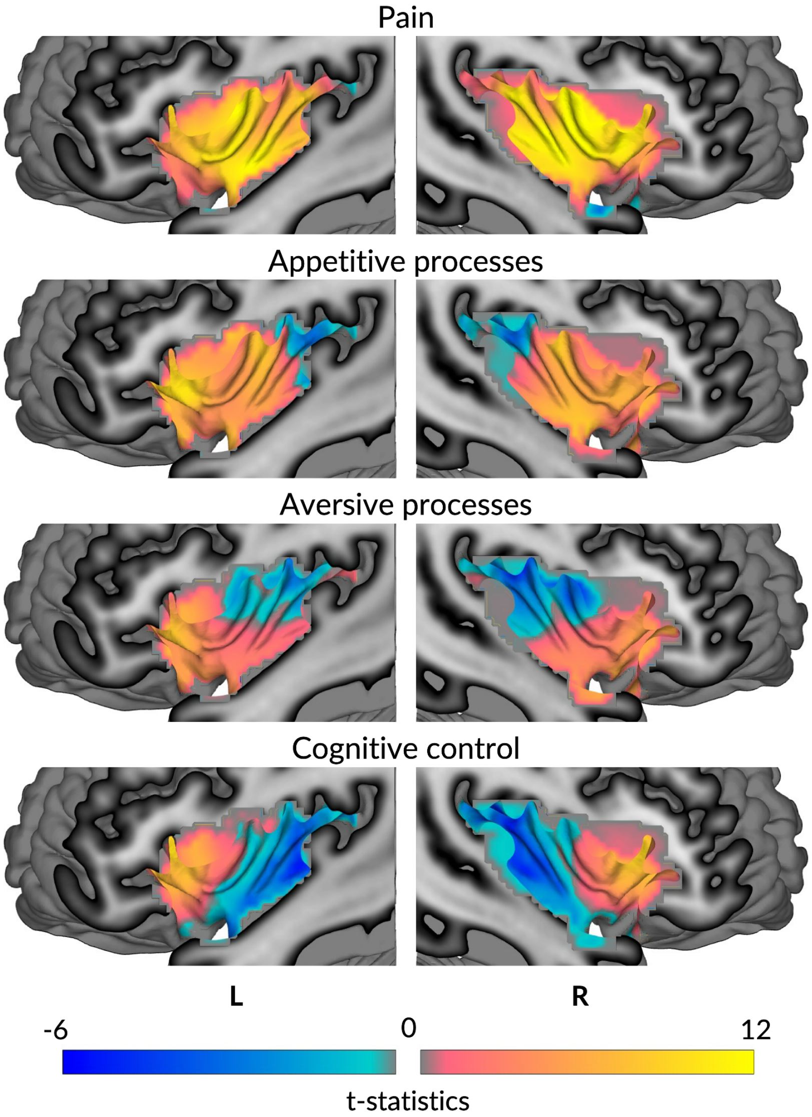

Supplementary Fig. 2

Domain-level t-statistic maps (unthresholded) for global insular activation across domains. Statistical parametric maps showing t-values from one-sample t-tests (n=135 per domain) for mean activation values normalized by each study's average voxel-wise between-subject SD to account for differences in signal scale across studies. Pain shows stronger and more widespread activation throughout the insula compared to other domains. Color scale represents t-statistic values.

Pain-selective zones with L2 normalization

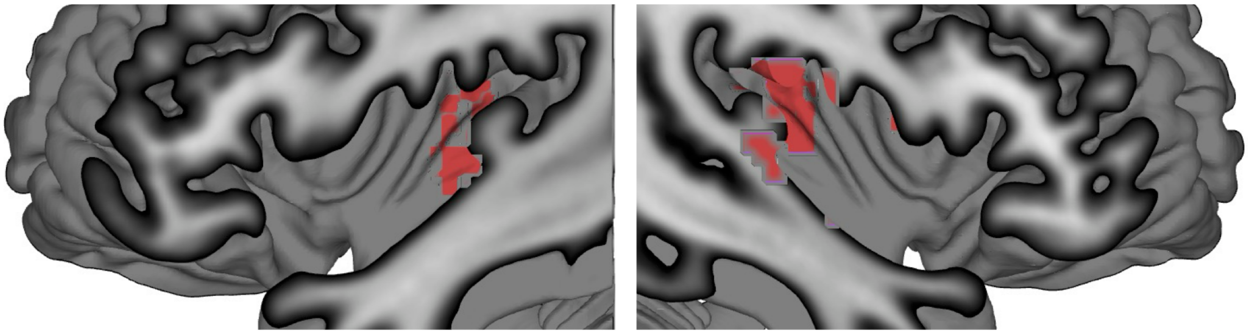

Supplementary Fig. 3

L2 normalization reveals pain-selective regions in posterior insula. Domain-selective zones identified using L2 normalization, which scales activation patterns to unit length without mean centering. In contrast to z-scoring normalization (used in the main analysis), which standardizes both mean and variance across voxels, L2 normalization maintains the sign and spatial distribution of activation patterns. This reveals pain-selective zones in the posterior insula that were obscured in the main analysis, confirming that pain-related signals are present in posterior regions but are masked in our primary analysis due to pain's significantly higher whole-insula activation compared to other domains.

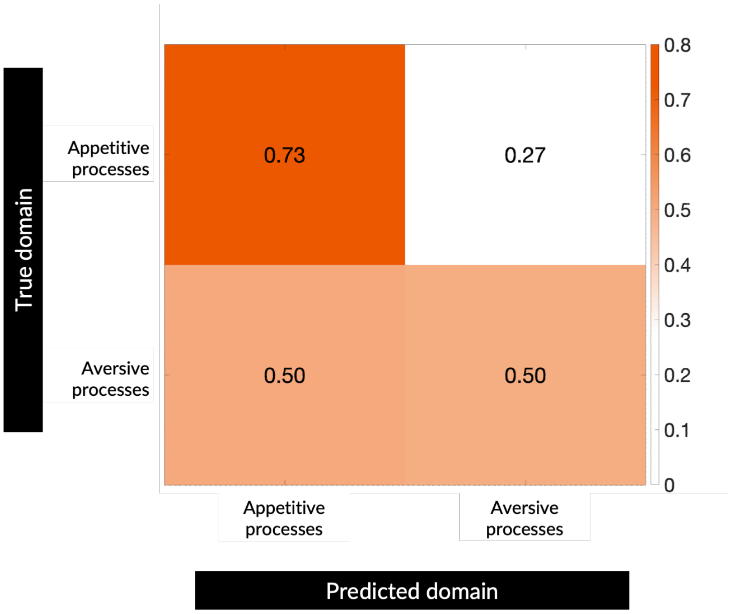

Supplementary Fig. 4

Pairwise classification between appetitive and aversive processes. To further investigate the multiclass classification patterns observed in Fig. 2b, we trained binary SVM classifiers specifically between appetitive and aversive processes. When classifying appetitive processes, accuracy was above chance (73%), but when classifying aversive processes, accuracy was at chance level (50%). This pattern suggests that while appetitive processes have distinct neural representations, aversive processes share substantial neural patterns with a subset of appetitive processes or potentially greater heterogeneity in their neural representations, which may result in more conservative results in identifying aversive-selective zones.

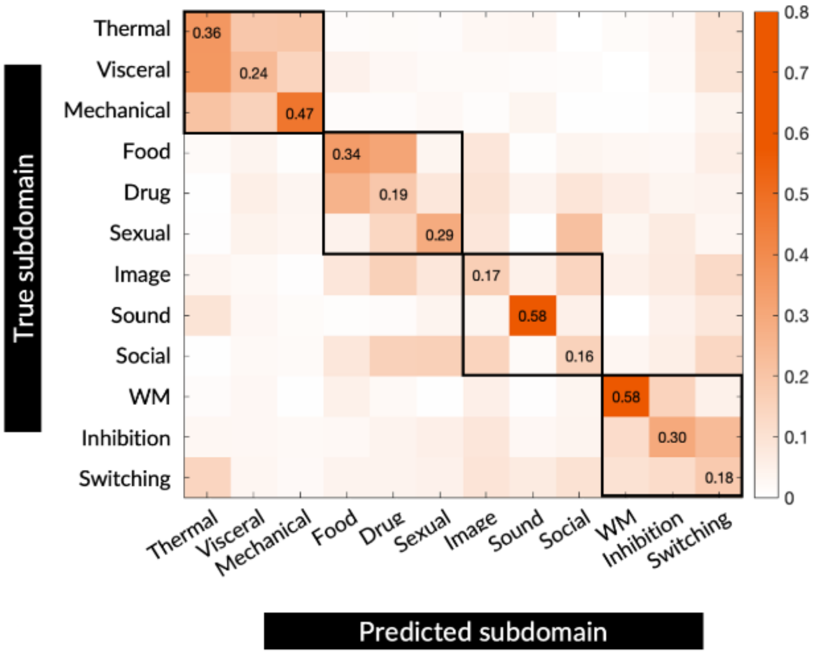

Supplementary Fig. 5

Subdomain classification using insular activation patterns. Confusion matrix shows prediction accuracies for multiclass SVM classifiers trained to discriminate between 12 subdomains (3 per domain) using the same leave-one-study-out scheme as domain classification (see Methods for details). Classifiers performed above chance (8.3%) for all subdomains (mean=32.11%, range: 15.6% for aversive social interaction to 57.8% for working memory), with mechanical pain, aversive sound, and working memory showing higher discriminability compared to other subdomains.

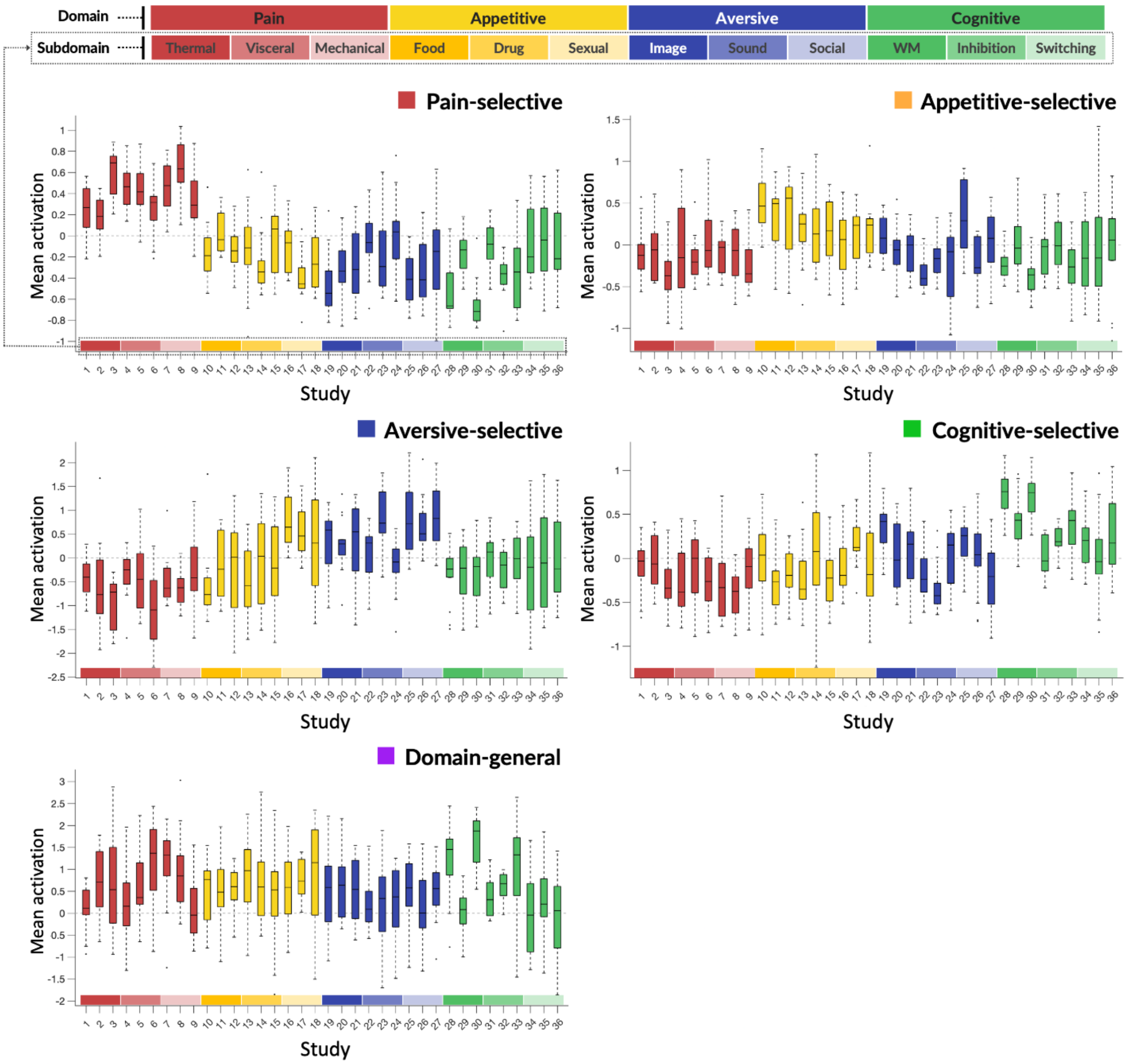

Supplementary Fig. 6

Activation profiles of insular zones. Mean z-scored contrast coefficients for domain-general and domain-selective zones across all 36 individual studies, grouped by domains and subdomains. Zones were identified using Bayes Factor analysis at the domain level (aggregating across all studies within each domain); this figure displays the activation values from these domain-defined zones separated by individual study to show consistency of activation profiles across studies and subdomains. Domain-general zones show high activation across all domains, while domain-selective zones show high activation for their designated domain and low/no activation for other domains. These patterns are consistent across studies and subdomains within each domain with few exceptions. The centerline shows the median; box edges represent first (25th percentile) and third (75th percentile) quartiles, with the box length showing the interquartile range (IQR, middle 50% of data). Whiskers extend to the most extreme values within 1.5 × IQR from the box edges, and points beyond the whiskers indicate outliers. Source data are provided as a Source Data file.

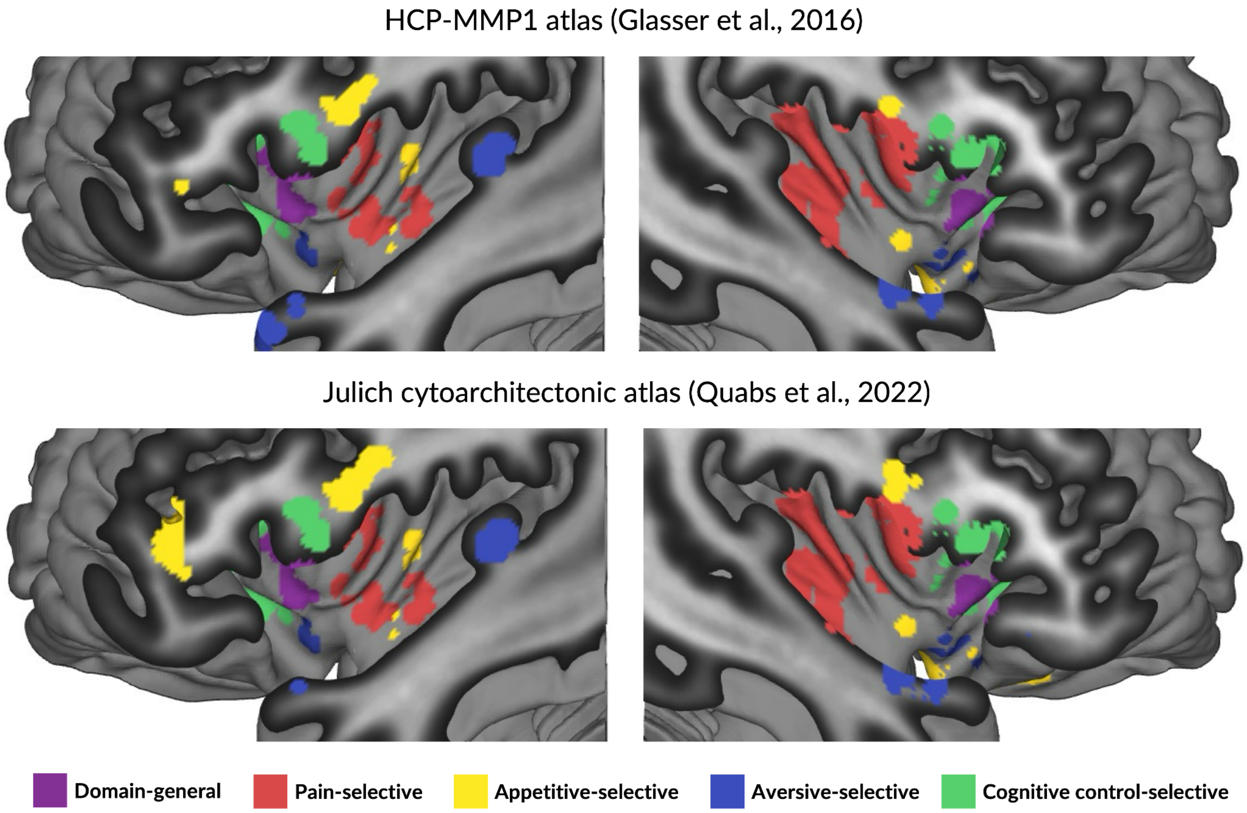

Supplementary Fig. 7

Domain-general and domain-selective zones using expanded anatomical definition including opercular regions. Analysis using masks that include both insula proper and adjacent opercular regions from Julich-Brain Cytoarchitectonic Atlas (top) and HCP-MMP1.0 Atlas (bottom). This analysis revealed similar domain-general and domain-selective patterns in opercular areas with known structural connections to the insula[9]. For example, pain-selective activation appeared primarily in OP5 and domain-general activation was found in OP7 and OP9, consistent with their connectivity to mid-posterior and dorsal anterior insula.

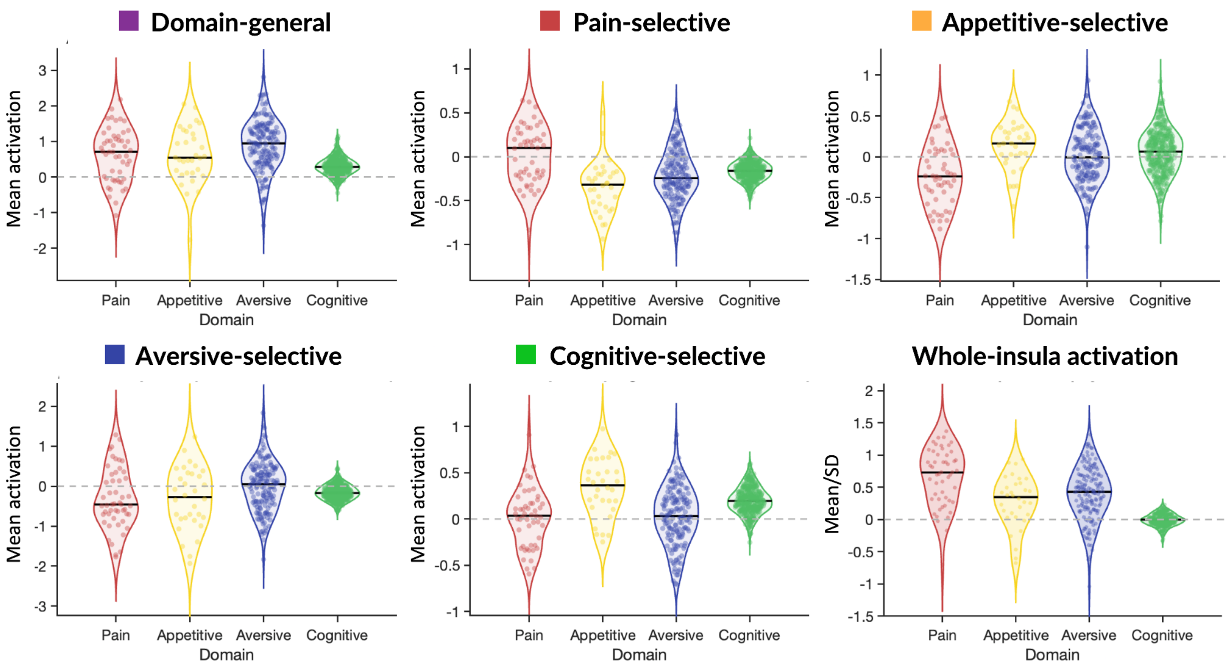

Supplementary Fig. 8

Validation of insular zones using independent datasets. Activation profiles from independent validation datasets (n=608) for the five identified functional zones. First five panels show mean activation within domain-general, pain-selective, appetitive-selective, aversive-selective, and cognitive-selective zones. The bottom last panel shows global insula activation (mean/SD) across domains in the validation datasets. Source data are provided as a Source Data file.

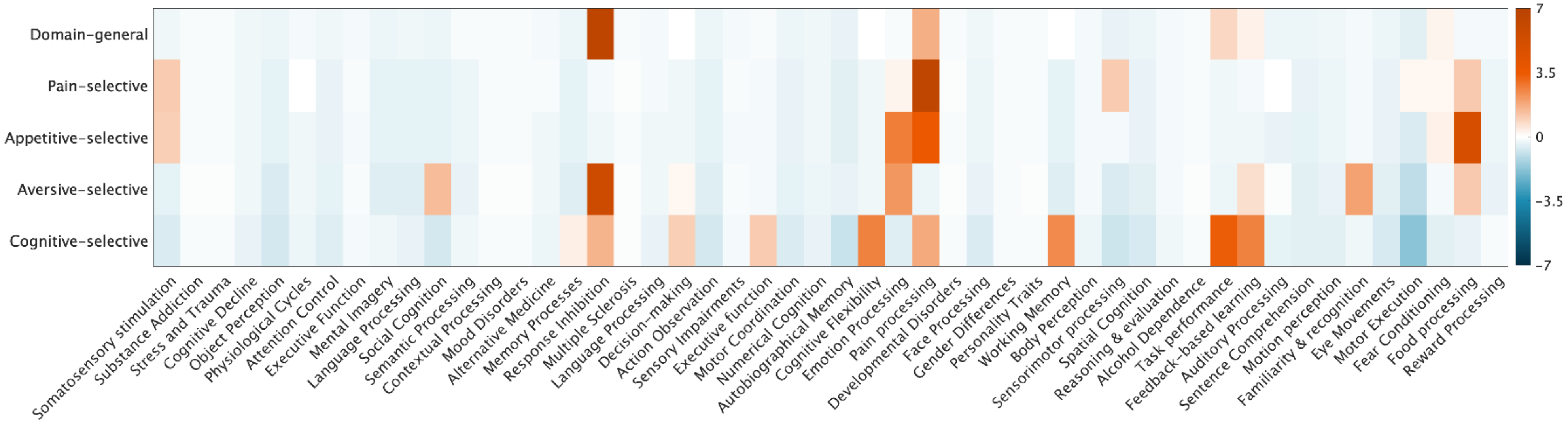

Supplementary Fig. 9

Complete topic-level decoding of insular zones using Neurosynth. Heatmap shows standardized point-biserial correlations between domain-general and domain-selective insular zones and all 50 psychological topic maps.

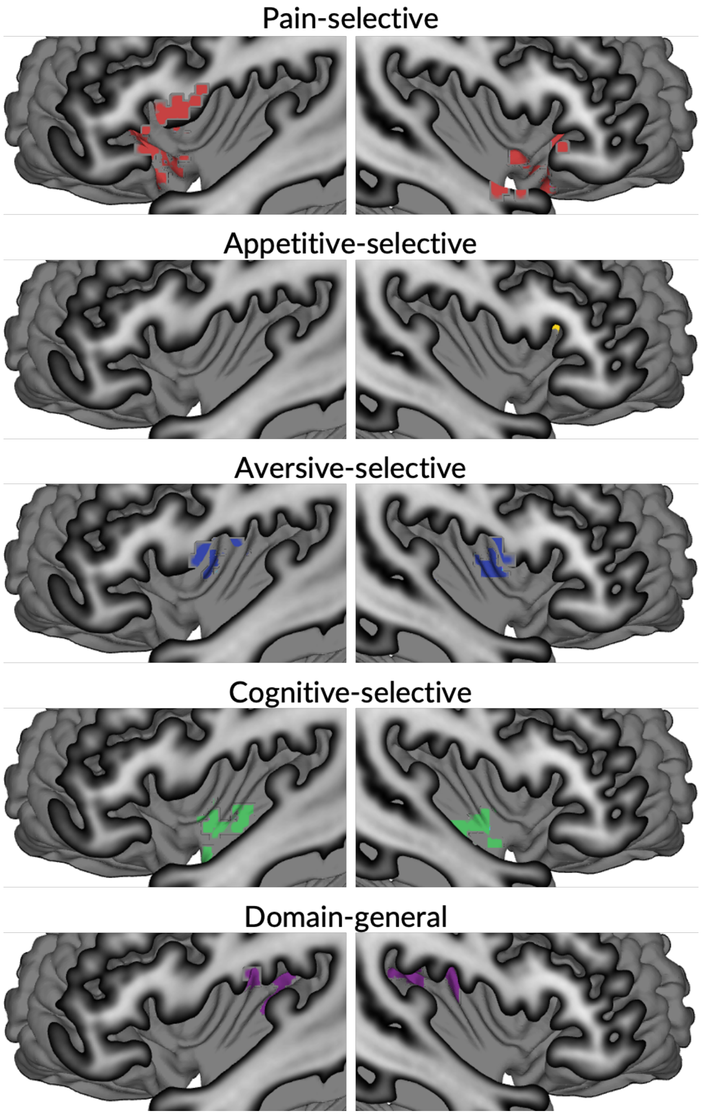

Supplementary Fig. 10

Deactivation-based functional zones in the insula. Domain-general zones identified from deactivation patterns were predominantly located in the posterior-most portion of the insula. Domain-selective deactivation zones showed distinct distributions: pain-selective in dorsal and ventral anterior insula, appetitive-selective in left dorsal anterior insula, aversive-selective in dorsal mid insula, and cognitive control-selective in ventral mid insula.

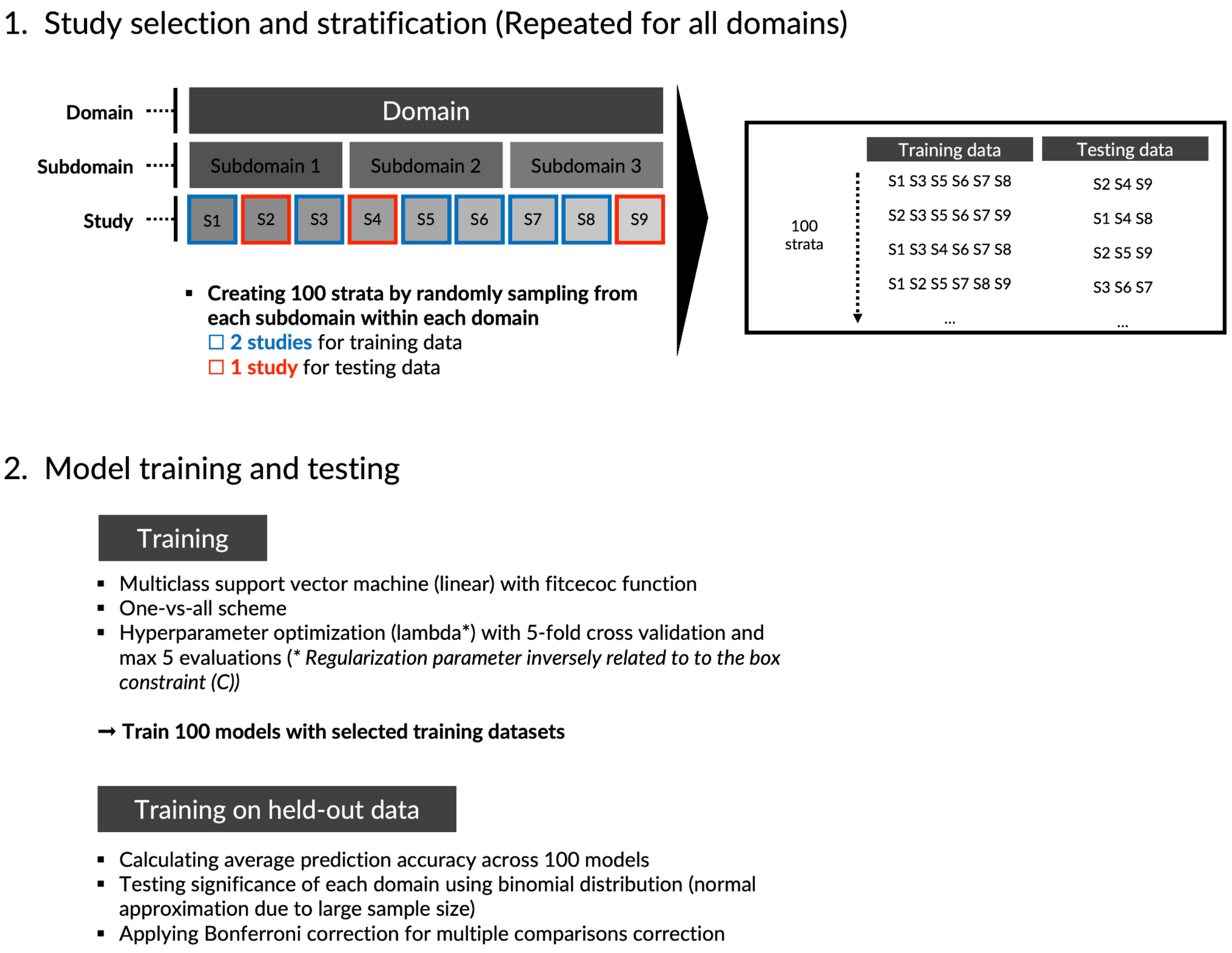

Supplementary Fig. 11

Schematic description of study selection and training and testing of multi-class Support Vector Machine (SVM) model. Part 1 shows study selection and stratification for one example domain. Each domain comprises three subdomains with three studies each (9 studies total per domain). For each iteration, one study per subdomain is held out as the test set (red border), while the remaining two serve as training data (blue border), ensuring the classifier is tested on entirely independent studies. Different held-out combinations are systematically sampled across 100 iterations. Part 2 shows the training and testing procedure. Training data from all four domains are used to fit a multi-class linear SVM with a one-vs-all scheme and 5-fold cross-validation for hyperparameter optimization (box constraint). The trained classifier is then applied to the held-out studies to predict domain labels. Classification accuracy is averaged across all 100 test sets to estimate out-of-study generalization performance.

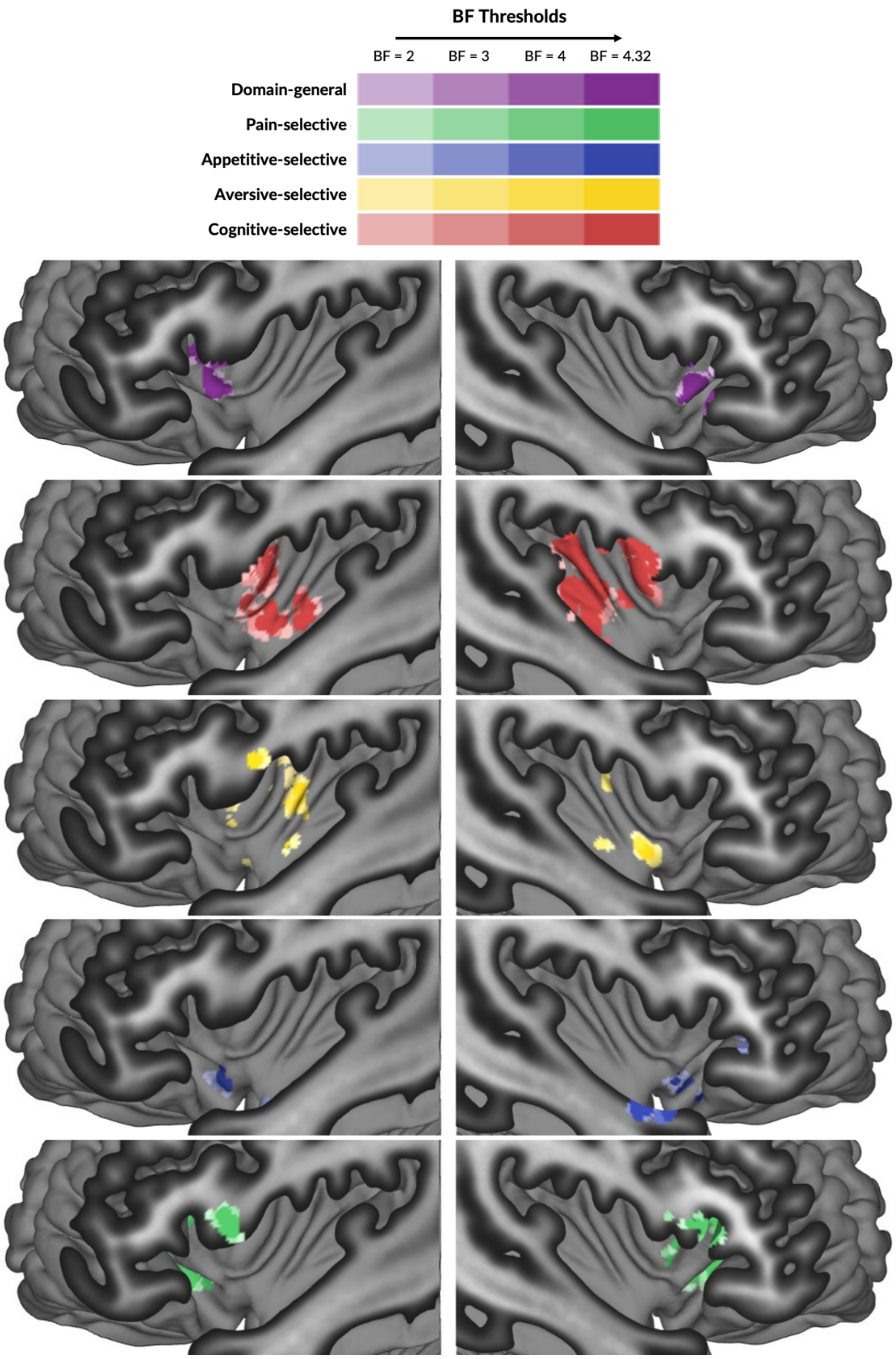

Supplementary Fig. 12

Domain-general and domain-selective zones at different Bayes Factor thresholds (BF = 2, 3, 4, and 4.32). Each row of the figure and color legend shows a different zone type: domain-general (purple), pain-selective (red), appetitive-selective (yellow), aversive-selective (blue), and cognitive control-selective (green). Left and right columns show left and right hemispheres,

respectively. Color intensity indicates the BF threshold at which each voxel survives, with lighter shades corresponding to lower thresholds and darker shades to higher thresholds from BF = 2 (lowest) to 4.32 (highest, the threshold used in the main analysis). Voxels surviving higher thresholds are spatial subsets of those at lower thresholds. The spatial extent and overall configuration of functional zones remain stable across thresholds, indicating that the identified zones are robust to the choice of BF threshold.

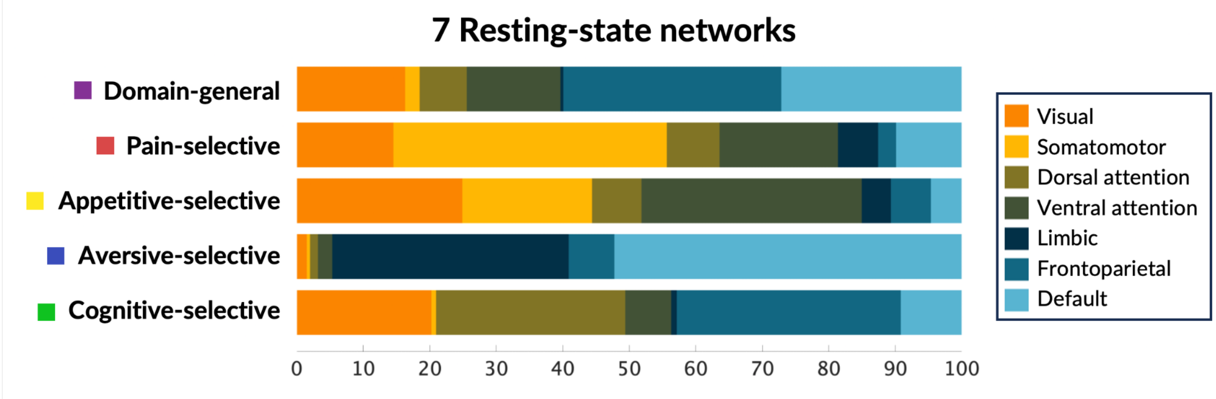

Supplementary Fig. 13

Resting-state network affiliations using unthresholded coactivation maps. This analysis shows network affiliations including subthreshold coactivated voxels, complementing the thresholded analysis in Fig. 5b. Source data are provided as a Source Data file.

Supplementary Tables

Supplementary Table 1

Domain-selective and domain-general insular clusters. Cluster information including center coordinates in standard MNI (Montreal Neurological Institute) space and parcellation labels using three existing atlases[7,8,10]

Insular cluster	L/R	Number of voxels	MNI Coordinates			Mean Bayes factors	Parcellations		
			X	Y	Z		7	10	8
Pain-selective	L	98	-40	2	2	22.80 [3.08, 97.98]	Ctx_PoI2_L	Posterior short gyrus (left)	L_Area_Id6
	R	222	38	-6	2		Ctx_PoI2_R	Anterior long gyrus (right)	R_Area_Op5
Appetitive-selective	L	9	-38	-8	2	6.88 [3.04,14.10]	Ctx_PoI2_L	Anterior long gyrus (left)	L_Area_Id5
	R	5	34	-12	-8		Ctx_PoI1_R	Posterior long gyrus (right)	R_Area_Ia3
Aversive-selective	L	33	-38	12	-16	10.12 [2.99, 25.73]	Ctx_AAIC_L	Anterior inferior gyrus (left)	L_Area_Id10
	R	42	36	14	-14		Ctx_AAIC_R	Anterior inferior gyrus (right)	R_Area_TI
Cognitive-selective	L	58	-28	20	8	15.46 [2.96, 51.72]	Ctx_FOP4_L	Anterior short gyrus (left)	L_Area_Id7
	R	84	30	20	4		Ctx_AVI_R	Anterior short gyrus (right)	R_Area_Id6
Domain-general	L	55	-42	16	-2	24.60 [3.13, 68.94] (Pain)<br>26.40 [2.98, 61.20] (Appetitive)<br>11.25 [3.00, 26.22] (Aversive)<br>20.70 [3.29, 56.28] (Cognitive)	Ctx_MI_L	Anterior short gyrus (left)	L_Area_Id6
	R	65	44	18	-4		Ctx_44_R	Anterior inferior gyrus (right)	R_Area_Id8'

Supplementary Table 2

Neurosynth topic maps selected for the current study and their correlations with each domain-general and domain-selective insular zone

Topic label	Domain-selective and domain-general insular zones					Neurosynth Topic ID
	Pain-selective	Appetitive-selective	Aversive-selective	Cognitive-selective	Domain-general	
**Somatosensory Stimulation**	1.0425	1.0072	-0.3993	-0.5538	-0.2708	0
**Substance Addiction**	-0.1215	-0.1551	-0.0977	-0.1420	-0.1541	2
**Stress and Trauma**	-0.1194	-0.1531	-0.0945	-0.1377	-0.1529	4
**Cognitive Decline**	-0.2390	-0.2606	-0.2701	-0.3774	-0.2204	5
**Object Perception**	-0.3890	-0.3947	-0.5712	-0.6783	-0.3045	6
**Physiological Cycles**	-0.0137	-0.2308	-0.2214	-0.3109	-0.2017	7
**Attention Control**	-0.3467	-0.3574	-0.4281	-0.4966	-0.2244	9
**Executive Function**	-0.1460	-0.1770	-0.1335	-0.1910	-0.1679	13
**Mental Imagery**	-0.3938	-0.3861	-0.4971	-0.2657	-0.3077	14
**Language Processing**	-0.4150	-0.4049	-0.5280	-0.3580	-0.2688	15
**Social Cognition**	-0.3836	-0.3900	1.3726	-0.6613	-0.3015	17
**Semantic Processing**	-0.2892	-0.3057	-0.3373	-0.2522	-0.1610	20
**Contextual Processing**	-0.1155	-0.1497	-0.0889	-0.1300	-0.1508	21
**Mood Disorders**	-0.1161	-0.1502	-0.0897	-0.1311	-0.1511	22
**Alternative Medicine**	-0.1163	-0.2323	-0.2238	-0.3142	-0.2026	23
**Memory Processes**	-0.4258	-0.4058	-0.5440	0.3508	-0.3257	24
**Response Inhibition**	-0.2176	-0.2414	5.5929	1.5550	6.5496	25
**Multiple Sclerosis**	-0.1042	-0.1395	-0.0723	-0.1073	-0.1444	30
**Language Processing (2)**	-0.2166	-0.2427	-0.2408	-0.3374	-0.2027	32
**Decision-making**	-0.2214	-0.2448	0.1886	0.9870	-0.0404	34
**Action Observation**	-0.3908	-0.3970	-0.4927	-0.6813	-0.3059	40
**Sensory Impairments**	-0.1578	-0.1876	-0.1509	-0.2147	-0.1746	46
**Executive Function (2)**	-0.1857	-0.2149	-0.1954	1.0597	-0.1109	49
**Motor Coordination**	-0.3480	-0.3589	-0.4304	-0.5963	-0.2820	51
**Numerical Cognition**	-0.2428	-0.2640	-0.2755	-0.3779	-0.2225	52

**Autobiographical Memory**	-0.4722	-0.4702	-0.3787	-0.8443	-0.3683	56
**Cognitive Flexibility**	-0.2249	-0.2479	-0.2493	2.6202	0.0488	58
**Emotion Processing**	0.2267	2.7119	2.1519	-0.5264	-0.1547	60
**Pain Processing**	6.5060	3.5111	-0.2935	1.7713	1.6717	61
**Developmental Disorders**	-0.1082	-0.1430	-0.0781	-0.1153	-0.1466	62
**Face Processing**	-0.3056	-0.3192	-0.3676	-0.5723	-0.2579	65
**Gender Differences**	-0.1306	-0.1599	-0.1110	-0.1602	-0.1593	66
**Personality Traits**	-0.1103	-0.1450	-0.0812	-0.1195	-0.1478	67
**Working Memory**	-0.4072	-0.4117	-0.5167	2.4255	-0.0402	68
**Body Perception**	-0.1919	-0.2182	-0.2009	-0.2829	-0.1938	70
**Sensorimotor Processing**	1.0622	-0.1656	-0.5678	-0.7829	-0.3348	72
**Spatial Cognition**	-0.3599	-0.3692	-0.4474	-0.6150	-0.2885	75
**Reasoning & Evaluation**	-0.1808	-0.2083	-0.1846	-0.2607	-0.1876	77
**Alcohol Dependence**	-0.1240	-0.1573	-0.1013	-0.1470	-0.1555	81
**Task Performance**	-0.2436	-0.2647	-0.2767	3.3536	0.8578	82
**Feedback-based Learning**	-0.2169	-0.2407	0.7106	2.6108	0.2978	85
**Auditory Processing**	-0.0534	-0.3679	-0.0616	-0.4330	-0.2877	86
**Sentence Comprehension**	-0.3753	-0.3831	-0.4516	-0.4684	-0.2941	87
**Motion Perception**	-0.2930	-0.3092	-0.3493	-0.4855	-0.2508	88
**Familiarity & Recognition**	-0.1762	-0.2041	1.9528	-0.2515	-0.1849	90
**Eye Movements**	-0.3571	-0.3667	-0.4432	-0.6138	-0.2869	93
**Motor Execution**	0.1785	-0.5495	-1.0050	-1.7706	-0.4728	95
**Fear Conditioning**	0.1967	0.3050	-0.2151	-0.4761	0.2336	97
**Food Processing**	1.1238	4.9280	1.1365	-0.3482	-0.2116	98
**Reward Processing**	-0.2900	-0.3177	-0.3428	-0.1450	-0.1861	99

Supplementary Table 3

List of highly matching cytoarchitectonic parcels for each insular zone

Insular cluster	L/R	Parcel	Dice coefficient	Cytoarchitectonic feature
Pain-selective	L	Id6 (L)	0.260	Dysgranular, Dorsal anterior
		Id3 (L)	0.160	Dysgranular, Dorsal anterior
		Id5 (L)	0.120	Agranular-dysgranular, Inferior posterior
	R	Id6 (R)	0.190	Dysgranular, Dorsal anterior
		Id3 (R)	0.160	Granular-dysgranular, Posterior
		Id2 (R)	0.150	Granular-dysgranular, Posterior
		Ig2 (R)	0.120	Granular-dysgranular, Posterior
Appetitive-selective	L	Id5 (L)	0.200	Agranular-dysgranular, Inferior
	R	Ia3 (R)	0.110	Agranular, Ventral anterior cluster
Aversive-selective	L	Id10 (L)	0.170	Agranular, Ventral anterior
	R	Id10 (R)	0.170	Agranular, Ventral anterior
		Id9 (R)	0.140	Agranular, Ventral anterior
Cognitive-selective	L	Id7 (L)	0.260	Dysgranular, Dorsal anterior
		Id6 (L)	0.120	Dysgranular, Dorsal anterior
	R	Id6 (R)	0.210	Agranular, Ventral anterior
		Id8 (R)	0.160	Dysgranular, Dorsal anterior
		Id7 (R)	0.150	Dysgranular, Dorsal anterior
		Id10 (R)	0.100	Agranular, Ventral anterior
Domain-general	L	Id6 (L)	0.260	Dysgranular, Dorsal anterior
	R	Id8 (R)	0.160	Agranular, Ventral anterior

Supplementary Table 4

Study info for main analysis

Study #	Domain	Subdomain	Publication	N	Contrasts	Stimulus/ Paradigm	Experimental design	Stimulus dynamics	N (female)	Mean Age	IRB/Ethics Approval Committee	MRI System
1	Pain	Thermal	Atlas et al. (2010)[11]	15	High vs low pain	Thermal stimulation	Event-related	10s duration (1.5s ramp up, 7s plateau, 1.5s ramp down); individually calibrated	19 (9)	25.5	Columbia University	1.5T GE Signa TwinSpeed Excite HD
2	Pain	Thermal	Wager et al. (2013)[12]	15	49.3° C vs baseline	Thermal stimulation	Event-related	10s duration; 44.3-49.3°C in 1°C increments	33 (22)	27.9	Columbia University	1.5T GE Signa TwinSpeed Excite HD
3	Pain	Thermal	Krishnan et al. (2016)[13]	15	High pain (48 degree) vs baseline	Thermal stimulation	Event-related	11s duration (2s ramp-up, 7s plateau, 2s ramp-down); 46, 47, 48°C fixed temperatures	28 (10)	25.2	University of Colorado Boulder	3T Siemens Tim Trio
4	Pain	Visceral	Kano et al. (2017)[14]	15	Distension vs baseline	Rectal distention	Event-related	18s distension (approximately 5s inflation, approximately 10s at threshold); individually calibrated	29 (15)	22.5	Tohoku University School of Medicine	3T Siemens TrioTIM
5	Pain	Visceral	Rubio et al. (2015)[15]	15	Distension vs baseline	Rectal distention	Block	18s distension (approximately 5s inflation, 13s at threshold); individually calibrated	15 (9)	24*	Comité de Protection des Personnes Sud Est V, France	3T Philips Achieva TX

6	Pain	Visceral	Coen et al. (2011)[16]	15	Distension vs rest	Esophageal pain	Event-related	1s phasic distension at pain tolerance threshold; individually calibrated	31 (16)	30	King's College London, UK	3T GE Signa Excite II
7	Pain	Mechanical	Kragel et al. (2018)[17]	15	7 kg/cm2 vs baseline	Pressure Stimulation	Event-related	10s duration; 7 kg/cm² to thumbnail	15 (4)	26.9	University of Colorado Boulder	3T Siemens TrioTIM
8	Pain	Mechanical	Čeko et al. (2022)[18]	15	4, 5, 6 and 7 kg/cm2 vs baseline	Pressure Stimulation	Event-related	10s duration; 4, 5, 6, 7 kg/cm² to thumbnail	15 (8)	24.2	University of Colorado Boulder	3T Siemens Prisma
9	Pain	Mechanical	Ashar et al. (Unpublished)	15	High vs low pressure	Pressure Stimulation	Event-related	6s duration; 4, 7 kg/cm² to thumbnail	141 (75)	41.7	University of Colorado Boulder	3T Siemens Prisma
10	Appetitive Processes	Food	Koban et al. (2023)[19]	15	Food cue vs. baseline (HC*)	Craving regulation with food cues	Event-related	6s image presentation	22 (9)	26.4	Columbia University	1.5T GE Signa TwinSpeed Excite HD
11	Appetitive Processes	Food	Koban et al. (2023)[19]	15	Food cue vs. baseline (HC*)	Craving regulation with food cues	Event-related	6s image presentation	18 (6)	42.1	Yale University	3T Siemens Magnetom Trio
12	Appetitive Processes	Food	Koban et al. (2023)[19]	15	Food cue vs. baseline (Smoker)	Craving regulation with food cues	Event-related	6s image presentation	21 (8)	26.8	Columbia University	1.5T GE Signa TwinSpeed Excite HD
13	Appetitive Processes	Drug	Koban et al. (2023)[19]	15	Drug cue vs. baseline (Drinker)	Craving regulation with drug cues	Event-related	6s image presentation	17 (7)	33.4	Yale University	3T Siemens Tim Trio
14	Appetitive Processes	Drug	Koban et al. (2023)[19]	15	Drug cue vs. baseline (Cocaine user)	Craving regulation with drug cues (cocaine)	Event-related	6s image presentation	21 (3)	43.5	Yale University	3T Siemens Magnetom Trio

15	Appetitive Processes	Drug	Koban et al. (2023)[19]	15	Drug cue vs. baseline (Smoker)	Craving regulation with drug cues (cigarette)	Event-related	6s image presentation	21 (8)	26.8	Columbia University	1.5T GE Signa TwinSpeed Excite HD
16	Appetitive Processes	Sexual	Wehrum et al. (2013)[20]	15	Sexual vs. neutral pictures	Sexually arousing images	Block design	3s per picture; 5 pictures per block	100 (50)	25.4	German Psychological Society	1.5T Siemens Symphony with quantum gradient system
17	Appetitive Processes	Sexual	Stark et al. (2019)[21]	15	Sexual video vs. baseline	Sexually arousing videos	Event-related	8s video presentation	70 (33)	25.7	German Psychological Society	3T Siemens Prisma
18	Appetitive Processes	Sexual	Kragel et al. (2019)[22]	15	Sexual images vs. baseline	Sexually arousing images from IAPS and GAPED	Event-related	4s image presentation	18 (10)	25	University of Colorado Boulder	3T Siemens Healthcare
19	Aversive Processes	Visual	Gianaros et al. (2014)[23]	15	Negative pictures vs baseline	Images from IAPS	Event-related	7s image presentation	183 (88)	42.7	University of Pittsburgh	1.5 and 3T GE Signa LX Horizon Echospeed ,
20	Aversive Processes	Visual	Yarkoni et al. (2011)[24]	15	Negative vs neutral pictures	Images from IAPS	Event-related	~10s image presentation (pooled from 5 studies)	108 (NR)	NR	Stanford University, Columbia University	1.5T GE Signa Twin Speed Excite HD scanner
21	Aversive Processes	Visual	Kober et al. (2019)[25]	15	Negative pictures vs baseline	Images from IAPS	Event-related	8s stimulus presentation	16 (5)	31.75	Columbia University	1.5T GE Signa Twin Speed Excite HD scanner
22	Aversive Processes	Auditory	Kragel et al. (2018)[17]	15	Unpleasant Sounds vs baseline	Sounds from IADS	Event-related	8s duration; sounds from IADS database	15 (7)	31.1	University of Colorado Boulder	3T Siemens Tim Trio

23	Aversive Processes	Auditory	Geuter et al. (2020)[26]; Kragel et al. (2018)[17]	15	Unpleasant Sounds vs baseline	Sounds from IADS	Event-related	8s duration; sounds from IADS database	15 (9)	24.4	University of Colorado Boulder	3T Siemens Tim Trio
24	Aversive Processes	Auditory	Ashar et al. (Unpublished)	15	Sound high vs. low in unpleasantne ss	Aversive sound (knife scraping on glass)	Event-related	6s duration; knife scraping sound at 2 intensity levels	141 (75)	41.7	University of Colorado Boulder	3T Siemens Prisma
25	Aversive Processes	Social	Kross et al. (2011)[27]	15	Images of ex-partner vs friend	Images of ex-partners	Event-related	15s image presentation	40 (21)	20.8	Columbia University	1.5T GE Signa TwinSpeed
26	Aversive Processes	Social	Krishnan et al. (2016)[13]	15	High pain images vs baseline	Images of others in pain	Event-related	11s image presentation	30 (12)	25.2	University of Colorado Boulder	3T Siemens Tim Trio
27	Aversive Processes	Social	Yu et al. (2020)[28]	15	Self-incorrect vs. baseline	Guilt from causing pain due to one's error	Event-related	3s feedback of performance	24 (11)	22	Peking University	3T Siemens Tesla Trio
28	Cognitive Control	WM	DeYoung et al. (2009)[29]	15	3-back blocks vs baseline	N-back (faces and words) task	Block	2s per stimulus	104 (59)	22.7	Washington University Medical Center	3T Siemens Allegra
29	Cognitive Control	WM	van Ast et al. (2016)[30]	15	N-back blocks vs baseline	N-back (words) task	Event-related	2s per stimulus	21 (10)	22.2	Columbia University	3T Philips Achieva
30	Cognitive Control	WM	Unpublished	15	Word event vs. fixation	Updating working memory task	Event-related	2s per stimulus	30 (16)	28.1	University of Colorado Boulder	3T Magnetom Trio
31	Cognitive Control	Inhibition	Aron et al. (2007)[31]	15	All trials vs baseline	Stop signal task	Event-related	~1s per stimulus	15 (5)	28.1	UCLA	3T Siemens Allegra
32	Cognitive Control	Inhibition	Xue et al. (2008)[32]	15	All trials vs baseline	Stop signal task	Event-related	~1s per stimulus	15 (9)	23.6	UCLA	3T Siemens Allegra
33	Cognitive Control	Inhibition	Unpublished	15	Antisaccade vs. fixation	Response inhibition task	Event-related	3.82s per stimulus (cue 2.32s, target 1.5s)	30 (16)	28.1	University of Colorado Boulder	3T MAGNETO M Trio

34	Cognitive Control	Switching	Unpublished	15	Switching vs. fixation	Set shifting task	Event-related	~3s	30 (16)	28.1	University of Colorado Boulder	3T MAGNETOM Trio
35	Cognitive Control	Switching	Wager et al. (2005)[33]	15	Switch vs. non-switch	Attention switching task (external)	Event-related	self-paced	39 (NR)	NR	University of Michigan	3T GE Signa
36	Cognitive Control	Switching	Wager et al. (2005)[33]	15	Switch vs. non-switch	Attention switching task (internal)	Event-related	self-paced	39 (NR)	NR	University of Michigan	3T GE Signa

** HC: healthy controls*

Supplementary Table 5

Study info for validation datasets

Domain	Publication	N*	Contrasts	Stimulus/ Paradigm	Experimental design	Stimulus dynamics	Mean Age	IRB/Ethics Approval Committee	MRI System
Pain	Woo et al. (2014)[34]	51	Heat pain vs warmth	Thermal stimulation	Event-related	15s duration (1.5s ramp up, 12s plateau, 1.5s ramp down); individually calibrated	20.8	Columbia University	1.5T GE Signa TwinSpeed Excite HD
Appetitive Process	MacNiven et al. (2018)[35]	32	Gain vs. no-gain anticipation	Monetary Incentive Delay (MID) task[36]	Event-related	cue, anticipation, target response, and outcome	32	Stanford University	3 Tesla GE Discover MR750
Aversive Process	Gianaros et al. (2020)[37]	160	Negative vs neutral images	Images from IAPS	Event-related	7s image presentation	39.7	University of Pittsburgh	3T Siemens Tim Trio
Cognitive control	Barch et al. (2013)[38]	365	2-back vs 0-back (place)	N-back (place) task	Block	2s per stimulus	28.7	N/A	Siemens 3T Connectome Skyra

** Represents the final number of participants included after preprocessing and quality control procedures.*

Supplementary References

1. Johnson, W. E., Li, C. & Rabinovic, A. Adjusting batch effects in microarray expression data using empirical Bayes methods. Biostatistics 8, 118–127 (2007).
2. Fortin, J.-P. et al. Harmonization of multi-site diffusion tensor imaging data. NeuroImage 161, 149–170 (2017).
3. Fortin, J.-P. et al. Harmonization of cortical thickness measurements across scanners and sites. NeuroImage 167, 104–120 (2018).
4. Pomponio, R. et al. Harmonization of large MRI datasets for the analysis of brain imaging patterns throughout the lifespan. NeuroImage 208, 116450 (2020).
5. Yu, M. et al. Statistical harmonization corrects site effects in functional connectivity measurements from multi-site fMRI data. Hum. Brain Mapp. 39, 4213–4227 (2018).
6. Nielson, D. M. et al. Detecting and harmonizing scanner differences in the ABCD study - annual release 1.0. 309260 Preprint at https://doi.org/10.1101/309260 (2018).
7. Glasser, M. F. et al. A multi-modal parcellation of human cerebral cortex. Nature 536, 171–178 (2016).
8. Quabs, J. et al. Cytoarchitecture, probability maps and segregation of the human insula. NeuroImage 260, 119453 (2022).
9. Quabs, J., Bittner, N. & Caspers, S. Structural Connectivity Differences Reflect Microstructural Heterogeneity of the Human Insular Cortex. Hum. Brain Mapp. 46, e70231 (2025).
10. Faillenot, I., Heckemann, R. A., Frot, M. & Hammers, A. Macroanatomy and 3D probabilistic atlas of the human insula. NeuroImage 150, 88–98 (2017).
11. Atlas, L. Y., Bolger, N., Lindquist, M. A. & Wager, T. D. Brain Mediators of Predictive Cue Effects on Perceived Pain. J. Neurosci. 30, 12964–12977 (2010).

12. Wager, T. D. et al. An fMRI-Based Neurologic Signature of Physical Pain. N. Engl. J. Med. 368, 1388–1397 (2013).
13. Krishnan, A. et al. Somatic and vicarious pain are represented by dissociable multivariate brain patterns. eLife 5, e15166 (2016).
14. Kano, M. et al. Influence of Uncertain Anticipation on Brain Responses to Aversive Rectal Distension in Patients With Irritable Bowel Syndrome. Psychosom. Med. 79, 988 (2017).
15. Rubio, A. et al. Uncertainty in anticipation of uncomfortable rectal distension is modulated by the autonomic nervous system — A fMRI study in healthy volunteers. NeuroImage 107, 10–22 (2015).
16. Coen, S. J. et al. Neuroticism Influences Brain Activity During the Experience of Visceral Pain. Gastroenterology 141, 909-917.e1 (2011).
17. Kragel, P. A. et al. Generalizable representations of pain, cognitive control, and negative emotion in medial frontal cortex. Nat. Neurosci. 21, 283–289 (2018).
18. Čeko, M., Kragel, P. A., Woo, C.-W., López-Solà, M. & Wager, T. D. Common and stimulus-type-specific brain representations of negative affect. Nat. Neurosci. 25, 760–770 (2022).
19. Koban, L., Wager, T. D. & Kober, H. A neuromarker for drug and food craving distinguishes drug users from non-users. Nat. Neurosci. 26, 316–325 (2023).
20. Wehrum, S. et al. Gender Commonalities and Differences in the Neural Processing of Visual Sexual Stimuli. J. Sex. Med. 10, 1328–1342 (2013).
21. Stark, R. et al. No Sex Difference Found: Cues of Sexual Stimuli Activate the Reward System in both Sexes. Neuroscience 416, 63–73 (2019).
22. Kragel, P. A., Reddan, M. C., LaBar, K. S. & Wager, T. D. Emotion schemas are embedded in the human visual system. Sci. Adv. 16 (2019).
23. Gianaros, P. J. et al. An Inflammatory Pathway Links Atherosclerotic Cardiovascular Disease Risk to Neural Activity Evoked by the Cognitive Regulation of Emotion. Biol. Psychiatry 75, 738–745 (2014).

24. Yarkoni, T., Poldrack, R. A., Nichols, T. E., Van Essen, D. C. & Wager, T. D. Large-scale automated synthesis of human functional neuroimaging data. Nat. Methods 8, 665–670 (2011).

25. Kober, H., Buhle, J., Weber, J., Ochsner, K. N. & Wager, T. D. Let it be: mindful acceptance down-regulates pain and negative emotion. Soc. Cogn. Affect. Neurosci. 14, 1147–1158 (2019).

26. Geuter, S. et al. Multiple Brain Networks Mediating Stimulus–Pain Relationships in Humans. Cereb. Cortex 30, 4204–4219 (2020).

27. Kross, E., Berman, M. G., Mischel, W., Smith, E. E. & Wager, T. D. Social rejection shares somatosensory representations with physical pain. Proc. Natl. Acad. Sci. 108, 6270–6275 (2011).

28. Yu, H. et al. A Generalizable Multivariate Brain Pattern for Interpersonal Guilt. Cereb. Cortex 30, 3558–3572 (2020).

29. DeYoung, C. G., Shamosh, N. A., Green, A. E., Braver, T. S. & Gray, J. R. Intellect as distinct from Openness: Differences revealed by fMRI of working memory. J. Pers. Soc. Psychol. 97, 883–892 (2009).

30. van Ast, V. A. et al. Brain Mechanisms of Social Threat Effects on Working Memory. Cereb. Cortex 26, 544–556 (2016).

31. Aron, A. R., Behrens, T. E., Smith, S., Frank, M. J. & Poldrack, R. A. Triangulating a Cognitive Control Network Using Diffusion-Weighted Magnetic Resonance Imaging (MRI) and Functional MRI. J. Neurosci. 27, 3743–3752 (2007).

32. Xue, G., Aron, A. R. & Poldrack, R. A. Common Neural Substrates for Inhibition of Spoken and Manual Responses. Cereb. Cortex 18, 1923–1932 (2008).

33. Wager, T. D., Jonides, J., Smith, E. E. & Nichols, T. E. Toward a taxonomy of attention shifting: individual differences in fMRI during multiple shift types. Cogn. Affect. Behav. Neurosci. https://doi.org/10.3758/CABN.5.2.127 (2005) doi:10.3758/CABN.5.2.127.

34. Woo, C.-W. et al. Separate neural representations for physical pain and social rejection. Nat. Commun. 5, 5380 (2014).

35. MacNiven, K. H. et al. Association of Neural Responses to Drug Cues With Subsequent Relapse to Stimulant Use. JAMA Netw. Open 1, e186466 (2018).

36. Knutson, B., Adams, C. M., Fong, G. W. & Hommer, D. Anticipation of Increasing Monetary Reward Selectively Recruits Nucleus Accumbens. J. Neurosci. 21, RC159–RC159 (2001).

37. Gianaros, P. J. et al. Affective brain patterns as multivariate neural correlates of cardiovascular disease risk. Soc. Cogn. Affect. Neurosci. 15, 1034–1045 (2020).

38. Barch, D. M. et al. Function in the human connectome: Task-fMRI and individual differences in behavior. NeuroImage 80, 169–189 (2013).

Full List of Members of The Affective Neuroimaging Consortium

Yoni K. Ashar: Division of General Internal Medicine, University of Colorado Anschutz Medical Campus, Aurora, CO, United States

Lauren Atlas: National Center for Complementary and Integrative Health, National Institute of Health, Bethesda, MD, United States; National Institute of Mental Health, National Institute of Health, Bethesda, MD, United States; National Institute on Drug Abuse, National Institute of Health, Baltimore, MD, United States

Lisa Feldman Barrett: Department of Psychology, College of Science, Northeastern University, Boston, MA, United States; Department of Psychiatry and the Athinoula A. Martinos Center for Biomedical Imaging, Massachusetts General Hospital, Boston, MA, United States

Benjamin Becker: Department of Psychology, The University of Hong Kong, Hong Kong, China

Luke Chang: Department of Psychological and Brain Sciences, Dartmouth College, Hanover, NH, United States

Luana Colloca: Department of Pain and Translational Symptom Science, School of Nursing, University of Maryland, Baltimore, MD, United States

Christopher G. Davey: Department of Psychiatry, The University of Melbourne, Melbourne, Australia

Sigrid Elsenbruch: Department of Medical Psychology and Medical Sociology, Center for Medical Psychology and Translational Neurosciences, Ruhr University Bochum, Bochum, Germany; Department of Neurology, Center for Translational and Behavioral Neuroscience (C-TNBS), University Hospital Essen, University of Duisburg-Essen, Essen, Germany

Miquel A. Fullana: Institut d'Investigacions Biomèdiques August Pi i Sunyer (IDIBAPS), Barcelona, Spain; Adult Psychiatry and Psychology Department, Institute of Neurosciences, Hospital Clinic, Barcelona, Spain

Valeria Gazzola: The Netherlands Institute for Neuroscience, KNAW research institute, Amsterdam, The Netherlands; Department of Psychology, University of Amsterdam, Amsterdam, The Netherlands

Ben J. Harrison: Department of Psychiatry, The University of Melbourne, Melbourne, Australia

Olivia K. Harrison: Department of Psychology, University of Otago, Dunedin, New Zealand; Translational Neuromodeling Unit, University of Zurich and ETH Zurich, Zurich, Switzerland

Alec Jamieson: Department of Psychiatry, The University of Melbourne, Melbourne, Australia

Christian Keysers: The Netherlands Institute for Neuroscience, KNAW research institute, Amsterdam, The Netherlands; Department of Psychology, University of Amsterdam, Amsterdam, The Netherlands

Brian Knutson: Department of Psychology, Stanford University, Stanford, CA, United States

Leonie Koban: Lyon Neuroscience Research Center (CRNL), CNRS, INSERM, Université Claude Bernard Lyon 1, Bron, France; Le Vinatier Psychiatrie Universitaire Lyon Métropole, Bron, France

Hedy Kober: Department of Psychology, University of California Berkeley, Berkeley, CA, United States; Department of Psychiatry, Yale University, New Haven, CT, United States

Kevin S. LaBar: Department of Psychology and Neuroscience, Duke University, Durham, NC, United States

Claus Lamm: Department of Cognition, Emotion, and Methods in Psychology, Faculty of Psychology, University of Vienna, Vienna, Austria

Martin Lindquist: Department of Biostatistics, Johns Hopkins Bloomberg School of Public Health, Baltimore, MD, United States

Tina Lonsdorf: Biological Psychology and Cognitive Neuroscience, Bielefeld University, Bielefeld, Germany; Institute for Systems Neuroscience, University Medical Center Hamburg Eppendorf, Hamburg, Germany

Marina Lopez-Sola: Serra Hunter Programme, Department of Medicine, School of Medicine and Health Sciences, University of Barcelona, Barcelona, Spain; Institute of Neuroscience, University of Barcelona, Barcelona, Spain; Institut d'Investigacions Mèdiques August Pi i Sunyer, Barcelona, Spain

Elizabeth Reynolds Losin: Department of Biobehavioral Health, Pennsylvania State University, University Park, PA, United States

Yina Ma: State Key Laboratory of Cognitive Neuroscience and Learning IDG/McGovern Institute for Brain Research, Beijing Normal University, Beijing, China

Christian J. Merz: Department of Cognitive Psychology, Institute of Cognitive Neuroscience, Faculty of Psychology, Ruhr University Bochum, Bochum, Germany

Hideki Mochizuki: Department of Dermatology and Cutaneous Surgery and Miami Itch Center, Miller School of Medicine, University of Miami, Miami, United States

Vitaly Napadow: Department of Physical Medicine and Rehabilitation, Spaulding Rehabilitation Hospital, Harvard Medical School, Charlestown, MA, United States; Athinoula A. Martinos Center for Biomedical Imaging, Massachusetts General Hospital, Harvard Medical School, Charlestown, MA, United States

Lauri Nummenmaa: Turku PET Centre and Turku University Hospital, Turku, Finland; Department of Psychology, University of Turku, Turku, Finland

Kyle Pattinson: Nuffield Department of Clin. Neurosciences, University of Oxford, Oxford, United Kingdom

Luiz Pessoa: Department of Psychology and Maryland Neuroimaging Center, University of Maryland, College Park, Maryland, United States; Department of Electrical and Computer Engineering, University of Maryland, College Park, Maryland, United States

Hilke Plassmann: INSEAD, Fontainebleau, France; Paris Brain Institute (ICM), Sorbonne University, Paris, France

Pierre Rainville: Department of Stomatology, Université de Montréal, Montréal, Canada; Research Center of the Montreal Geriatric University Institute, Montréal, Canada

Marianne Reddan: School of Psychology and Neuroscience, Center for Cognitive Neuroimaging, University of Glasgow, Glasgow, United Kingdom

Rebecca Saxe: Department of Brain and Cognitive Sciences, Massachusetts Institute of Technology, Cambridge, MA, United States; McGovern Institute for Brain Research, Massachusetts Institute of Technology, Cambridge, MA, United States

Daniela Schiller: Department of Psychiatry, Department of Neuroscience, Icahn School of Medicine, Mount Sinai, New York, NY, United States; Friedman Brain Institute, Icahn School of Medicine, Mount Sinai, New York, NY, United States

Alexander J. Shackman: Department of Psychology, University of Maryland, College Park, MD, United States; Neuroscience and Cognitive Science Program, University of Maryland, College Park, MD, United States; Maryland Neuroimaging Center, University of Maryland, College Park, MD, United States

Dana Small: Department of Neurology and Neurosurgery, Department of Medicine, and Department of Psychology, McGill University, Montréal, Canada; Research Institute of the McGill University Health Centre, Montréal, Canada; Modern Diet and Physiology Research Center (MDPRC), Montréal, Canada

Jason F. Smith: Department of Psychology, University of Maryland, College Park, MD, United States

Carles Soriano-Mas: Department of Psychiatry, Bellvitge University Hosp., Bellvitge Biomed. Institute-IDIBELL, Barcelona, Spain; CIBERSAM, Madrid, Spain; Department of Social Psychology and Quantitative Psychology, Institute of Neurosciences, University of Barcelona, Spain

Rudolf Stark: Department of Psychotherapy and Systems Neuroscience, Justus-Liebig-University Giessen, Giessen, Germany; Bender Institute of Neuroimaging, Justus-Liebig-University Giessen, Giessen, Germany; Center of Mind, Brain, and Behavior, Universities of Marburg and Giessen, Giessen, Germany

Bram Vervliet: Department of Brain and Cognition, KU Leuven, Leuven, Belgium; Leuven Brain Institute, KU Leuven, Leuven, Belgium

Choong-Wan Woo: Center for Neuroscience Imaging Research, Institute for Basic Science, Suwon, Republic of Korea; Department of Biomedical Engineering, Sungkyunkwan University, Suwon, Republic of Korea; Department of Intelligent Precision Healthcare Convergence, Sungkyunkwan University, Suwon, Republic of Korea; Department of Brain Science and Engineering, Sungkyunkwan University, Suwon, Republic of Korea

Fadel Zeidan: Department of Anesthesiology, University of California San Diego, La Jolla, CA, United States

Feng Zhou: Faculty of Psychology, Southwest University, Chongqing, China
