# Supplementary material for: Convergent and selective representations of pain, appetitive processes, aversive processes, and cognitive control in the insula
Source: Nat Commun. 2026 Apr 14;17:5186. doi: 10.1038/s41467-026-71568-9 (PMC13254212; doi:10.1038/s41467-026-71568-9)
Supplement: Supplementary file 2 — Reporting Summary [file 41467_2026_71568_MOESM2_ESM.pdf]

nature portfolio

Corresponding author(s): Tor D. Wager & Mijin Kwon

Last updated by author(s): Mar 17, 2026

Reporting Summary

Nature Portfolio wishes to improve the reproducibility of the work that we publish. This form provides structure for consistency and transparency in reporting. For further information on Nature Portfolio policies, see our Editorial Policies and the Editorial Policy Checklist.

Statistics

For all statistical analyses, confirm that the following items are present in the figure legend, table legend, main text, or Methods section.

n/a	Confirmed	
☐	☒	The exact sample size (*n*) for each experimental group/condition, given as a discrete number and unit of measurement
☐	☒	A statement on whether measurements were taken from distinct samples or whether the same sample was measured repeatedly
☐	☒	The statistical test(s) used AND whether they are one- or two-sided<br>*Only common tests should be described solely by name; describe more complex techniques in the Methods section.*
☒	☐	A description of all covariates tested
☐	☒	A description of any assumptions or corrections, such as tests of normality and adjustment for multiple comparisons
☐	☒	A full description of the statistical parameters including central tendency (e.g. means) or other basic estimates (e.g. regression coefficient) AND variation (e.g. standard deviation) or associated estimates of uncertainty (e.g. confidence intervals)
☐	☒	For null hypothesis testing, the test statistic (e.g. *F*, *t*, *r*) with confidence intervals, effect sizes, degrees of freedom and *P* value noted<br>*Give P values as exact values whenever suitable.*
☐	☒	For Bayesian analysis, information on the choice of priors and Markov chain Monte Carlo settings
☒	☐	For hierarchical and complex designs, identification of the appropriate level for tests and full reporting of outcomes
☐	☒	Estimates of effect sizes (e.g. Cohen's *d*, Pearson's *r*), indicating how they were calculated

Our web collection on statistics for biologists contains articles on many of the points above.

Software and code

Policy information about availability of computer code

Data collection	Data collection methods varied across studies. Details are provided in the original publications listed in Supplementary Table 4 (main analysis) and Supplementary Table 5 (validation analysis).
Data analysis	Analysis was conducted using CANLab and SPM software implemented in MATLAB. Code for implementing all analyses is available at https://github.com/canlab/, https://github.com/mijinjkwon/proj_insula_anic, and www.fil.ion.ucl.ac.uk/spm/software/.

For manuscripts utilizing custom algorithms or software that are central to the research but not yet described in published literature, software must be made available to editors and reviewers. We strongly encourage code deposition in a community repository (e.g. GitHub). See the Nature Portfolio guidelines for submitting code & software for further information.

Data

Policy information about availability of data

All manuscripts must include a data availability statement. This statement should provide the following information, where applicable:

- Accession codes, unique identifiers, or web links for publicly available datasets
- A description of any restrictions on data availability
- For clinical datasets or third party data, please ensure that the statement adheres to our policy

The fMRI data from main-analysis studies 1, 2, 4, 5, 7, 8, 19, 20, 22, 23, 25, 26, 28, 29, 31, and 32 are available at https://doi.org/10.6084/m9.figshare.24033402.v2. Data from studies 3 and 6 are available at https://neurovault.org/collections/8707/. Data from the validation dataset for cognitive control (n-back working memory task) are available from the Human Connectome Project database (https://www.humanconnectome.org). The remaining main-analysis and validation datasets are

available upon request from the corresponding authors of the individual studies. The Neurosynth dataset used in the analyses is available at https://github.com/canlab/Neuroimaging_Pattern_Masks/tree/master/neurosynth, the cytoarchitecture maps at https://github.com/canlab/Neuroimaging_Pattern_Masks/tree/master/Atlases_and_parcellations/2020_JulichBrain_v3.0.3, and the neurotransmitter receptor/transporter maps at https://github.com/canlab/Neuroimaging_Pattern_Masks/tree/master/Atlases_and_parcellations/2022_Hansen_PET_tracer_maps.

Research involving human participants, their data, or biological material

Policy information about studies with human participants or human data. See also policy information about sex, gender (identity/presentation), and sexual orientation and race, ethnicity and racism.

Reporting on sex and gender	Sex, determined by self-report across all studies for 36 study contrasts in the main analysis and 4 in the validation analysis, is documented in Supplementary Table 4 and Supplementary Table 5, respectively. While the study included both male and female participants, it was not designed or powered to systematically examine sex differences in insular function, and therefore no sex-based analyses were performed. Gender information beyond binary sex was not uniformly collected across individual studies, precluding gender-based analyses.
Reporting on race, ethnicity, or other socially relevant groupings	Race, ethnicity, and other socially relevant grouping information was collected differently across the studies included in the main and validation analyses. The current study was not designed or powered to investigate differences among racial, ethnic, or other social groups in insular function, and therefore no analyses were performed examining these factors.
Population characteristics	Population characteristics are summarized in Supplementary Table 4 (main analysis) and Supplementary Table 5 (validation). Specific inclusion/exclusion criteria are detailed in the original publications.
Recruitment	Recruitment methods varied across studies and are detailed in the original publications. Our systematic sampling across multiple studies and sites helps mitigate potential study-specific recruitment biases.
Ethics oversight	Each study in the main analysis and validation analysis was approved by its respective institutional review board, as listed in Supplementary Table 4 and Supplementary Table 5, respectively.

Note that full information on the approval of the study protocol must also be provided in the manuscript.

Field-specific reporting

Please select the one below that is the best fit for your research. If you are not sure, read the appropriate sections before making your selection.

[x] Life sciences [ ] Behavioural & social sciences [ ] Ecological, evolutionary & environmental sciences

For a reference copy of the document with all sections, see nature.com/documents/nr-reporting-summary-flat.pdf

Life sciences study design

All studies must disclose on these points even when the disclosure is negative.

Sample size	The current analysis included 540 participants (15 participants sampled from 36 studies). Sample size determinations for individual studies are detailed in their original publications.
Data exclusions	No data were excluded from the analysis.
Replication	Direct replication was attempted by validating primary findings against independent datasets for each domain.
Randomization	As a mega-analysis of existing studies, this was not a randomized study. Contrasts were constructed within subjects to compare effects across studies. Participants were recruited independently for each study. Domain assignment was determined by the experimental manipulation used in each study (e.g., studies using thermal stimulation were assigned to the pain domain).
Blinding	Investigators were not blinded during data analysis but were unaware of group comparisons during original data collection.

Reporting for specific materials, systems and methods

We require information from authors about some types of materials, experimental systems and methods used in many studies. Here, indicate whether each material, system or method listed is relevant to your study. If you are not sure if a list item applies to your research, read the appropriate section before selecting a response.

Materials & experimental systems

n/a	Involved in the study
x	Antibodies
x	Eukaryotic cell lines
x	Palaeontology and archaeology
x	Animals and other organisms
x	Clinical data
x	Dual use research of concern
x	Plants

Methods

n/a	Involved in the study
x	ChIP-seq
x	Flow cytometry
	x MRI-based neuroimaging

Plants

Seed stocks: Not applicable to the current study

Novel plant genotypes: Not applicable to the current study

Authentication: Not applicable to the current study

Magnetic resonance imaging

Experimental design

Design type: Multiple experimental designs were used across 36 studies. Our approach aims to generalize across these methodological factors.

Design specifications: Trial number, stimulus duration, and scan length varied across studies. Details are provided in the original publications referenced in Supplementary Table 4.

Behavioral performance measures: Behavioral data were not included.

Acquisition

Imaging type(s): BOLD fMRI

Field strength: 1.5 and 3 Tesla

Sequence & imaging parameters: EPI (standard and multi-band) and spiral in-out sequences were used for data acquisition. Details are provided in the original publications listed in Supplementary Table 4.

Area of acquisition: Whole brain scans were used.

Diffusion MRI: [ ] Used [x] Not used

Preprocessing

Preprocessing software: Each study used different preprocessing pipeline and software. Details are provided in the original publications listed in Supplementary Table 4.

Normalization: Images were normalized to Montreal Neurological Institute (MNI) space using study-specific MNI templates.

Normalization template: The templates used depend on the study but all studies used MNI templates

Noise and artifact removal: Regression of motion parameters was performed in all studies.

Volume censoring: Outlier timepoints identified via Mahalanobis distance using a chi-square test were excluded in some studies.

Statistical modeling & inference

Model type and settings: Data used were all 2nd level contrast maps sampled from 36 studies and they were then evaluated by Bayes factor for the purpose of conjunction analysis to identify domain-general and domain-selective voxels.

Effect(s) tested	Bayes Factors were calculated for each functional domain to compare evidence for alternative and null hypotheses using JZS prior (Rouder et al., 2009). Thresholds were set at 4.32 for alternative and 0.23 (1/4.32) for null hypothesis, corresponding to FDR-corrected $q < 0.01$. Voxels were classified as activated when showing both BF > 4.32 and positive t-statistics. Evidence for no activation included cases of either BF < 0.23 or BF > 4.32 with negative t-statistics. With conjucntion analysis, voxels activated in all four domains were classified as domain-general, while voxels activated in one domain with evidence for no activation in other domains were classified as domain-selective.

Specify type of analysis: ☐ Whole brain ☐ ROI-based ☒ Both

Anatomical location(s): Insula

Statistic type for inference (See Eklund et al. 2016)	Voxel-wise statics were used.
Correction	FDR correction

Models & analysis

n/a	Involved in the study
☒	☐ Functional and/or effective connectivity
☒	☐ Graph analysis
☒	☐ Multivariate modeling or predictive analysis
