# Supplementary material for: Convergent and selective representations of pain, appetitive processes, aversive processes, and cognitive control in the insula
Source: Nat Commun. 2026 Apr 14;17:5186. doi: 10.1038/s41467-026-71568-9 (PMC13254212; doi:10.1038/s41467-026-71568-9)
Supplement: Supplementary file 3 — Transparent Peer Review file [file 41467_2026_71568_MOESM3_ESM.pdf]

nature portfolio

Peer Review File

Convergent and selective representations of pain, appetitive processes, aversive processes, and cognitive control in the insula

Corresponding Author: Professor Tor Wager

This file contains all reviewer reports in order by version, followed by all author rebuttals in order by version.

Version 0:

Reviewer comments:

Reviewer #1

(Remarks to the Author)
Mijin Kwon and colleagues present an important study on the convergent and selective representation of functional domains in the human insula, employing Bayesian mega-analysis alongside functional and microstructural/transmitter-related decoding.

The following key results are reported:
1. Across four tested domains representing key aspects of insular functionality (pain, aversion, appetite, and cognitive control), the insula exhibited both convergent zones—responding to multiple domains—and selective zones, which were domain-specific.
2. The results are supported by functional decoding and coactivation analyses.
3. The borders between domains align, at least partially, with microstructural parcellation boundaries and variations in neurotransmitter profiles.

Overall, the study was carefully conducted using a straightforward, valid, and reliable methodology. The data analysis and interpretation are thorough, and all claims are well supported by the underlying data. Limitations are carefully acknowledged and discussed. The manuscript is well written, presenting its findings with coherence and clarity, complemented by clear and easily interpretable figures.

The reported findings are of considerable importance not only for researchers focusing on the insula but also for the broader neuroscience community. The insula, particularly the anterior part, is among the most frequently activated structures in functional neuroimaging studies. However, a precise characterization of its broad functionality has been largely missing. This study builds upon and extends the work of Kurth et al. (2010), which was, so far (at least to my knowledge), the only comprehensive study on this topic. Given the insula's increasing recognition as a common substrate in numerous neuropsychiatric disorders, establishing a detailed structural/functional anatomical model of this brain region is crucial. By projecting functional results onto a microstructural framework, this study represents an important step toward such an integrated model of the human insula.

Below are some comments that may be helpful for a minor revision of the manuscript:

1. Clarification of microstructural-related claims:
In both the methods and results sections, you state that the microstructural organization of the human insula consists of "16 areas grouped into four clusters (posterior, dorsal anterior, inferior posterior, and ventral anterior) based on cytoarchitectonic characteristics (granular-dysgranular, dysgranular, dysgranular-agranular, and agranular)." However, this statement regarding the clustering solution is not accurate. The cluster analysis we conducted in our paper was based on the areas available at the time and did not include the areas of the ventral anterior insula and certain parts of the agranular ventral posterior pole/Limen insulae. While these areas are publicly available, the publication, along with an overall cluster analysis, is still in preparation and not yet published. Therefore, there is currently no comprehensive clustering solution incorporating all cytoarchitectonic areas, and no evidence supports the existence of a fourth fully agranular cluster in the ventral anterior insula as implied in this statement. A similar issue arises in Figure 5, where you depict an agranular ventral

anterior cluster. The greyed-out areas were not part of the cluster analysis and contain both agranular and dysgranular characteristics rather than being purely agranular.

Given that your analysis focuses on specific areas rather than a clustering solution and does not center on cytoarchitectonic details, I recommend omitting references to the clustering solution from the text and figure and instead referring directly to the areas.

In that regard, I noted some further minor issues in the manuscript that need clarification:
• "Pain-selective zones were a notable exception to this pattern of cytoarchitectonic associations, showing a complex pattern spanning multiple cytoarchitectonic clusters that both aligns with and diverges from previous findings." → Since a full cluster analysis covering all insular areas is not yet available, I suggest replacing "clusters" with "areas" to ensure accuracy.
• "Primarily in agranular insula, with appetitive-selective zones overlapping most strongly with the left inferior posterior cluster."
• "Aversive-selective zones in agranular regions (Id10) align with known limbic system projections and correspond to regions showing specific responses to negative emotional stimuli in electrophysiological recordings."
→ The region you describe here is not agranular. Id10 stands for "Insular dysgranular area 10"; therefore, it is not agranular.
• You state in the discussion that "Id6 showed hypervigilance and anxiety when stimulated."
→ While this is partially true, as mentioned in our paper, the majority of findings in this area indicate non-responsiveness or visceral sensations. Please revise this statement to be more precise, such as: "Id6 showed hypervigilance and anxiety in a minor subset of patients when stimulated."
• Further, you write: "As with resting-state networks, our functional insular zones mapped onto cytoarchitecture to some degree, but cytoarchitectonic boundaries were not sufficient to describe them completely." → Please note that your analysis utilized maximum probability maps, assigning each voxel to the area with the highest probability. As a result, activations near the borders between two areas may be difficult to interpret because there is still some probability that this activation is located within the neighboring area. Just be aware of the probabilistic nature of the microstructural maps. You might want to acknowledge this limitation in the cytoarchitectonic-related discussion part or limitations section.

2. References:
In all instances where you cited the microstructural insula atlas, such as in the statement:
"To distinguish between these possibilities and better understand the nature of these zones, we further characterized them through multiple levels of analysis: meta-analytic functional decoding (using Neurosynth19,42), coactivation with other brain regions66,67, cytoarchitectonic mapping23,68, and neurotransmitter system profiling69,70."
Please also include the work of Kurth, Grodzinsky, and Hein, as they all contributed to the insula microstructural atlas. The relevant references can be found in the caption of Figure 13 in our paper, "Cytoarchitecture, probability maps, and segregation of the human insula."

Furthermore, as you have discussed Kurth's work, "A link between the systems: functional differentiation and integration within the human insula revealed by meta-analysis," in detail in the introduction, it would be beneficial to briefly mention and compare his findings with your results in the discussion section. Since his results regarding a convergence zone are localized in a region comparable to your findings, such a comparison would further support your results and provide a clearer conceptual link between the arguments presented in the introduction and discussion.

Additionally, I suggest two more papers that directly link area Id7 with specific functions that should be at least cited or in the case of Sabat at al. briefly discussed in the discussion section:
M. Sabat, C. de Dampierre, & C. Tallon-Baudry, Evidence for domain-general arousal from semantic and neuroimaging meta-analyses reconciles opposing views on arousal, Proc. Natl. Acad. Sci. U.S.A. 122(6), e2413808122, https://doi.org/10.1073/pnas.2413808122 (2025).
Grodzinsky, Y., Deschamps, I., Pieperhoff, P. et al., Logical negation mapped onto the brain, Brain Struct Funct 225, 19–31 (2020). https://doi.org/10.1007/s00429-019-01975-w

Finally, in the discussion, you state:
"Specifically, we did not observe pain-selective activity in Ig1 (bilateral), Ig2 (left), and Id2 (left) of the dorsal posterior insula —regions where many previous studies found pain-selective activity54,55,118 and where direct stimulation reliably evokes pain29,56,119–121."
To the best of my knowledge, none of these studies directly associated their findings with microstructural areas, except for our paper, "Subjective states induced by intracranial electrical stimulation match the cytoarchitectonic organization of the human insula." Therefore, your claim here appears speculative and lacks direct supporting evidence. I recommend rephrasing this statement as:"In an anatomically comparable localization, many previous studies have reported pain-selective activity.

3. discussing network results:
In the discussion, you state:
"With respect to canonical resting-state cortical networks, domain-general and domain-selective insular zones and their coactivated systems were unevenly distributed across the networks, but did not align in a one-to-one fashion with them."
While this statement is profound based on your data, I believe there are some noteworthy trends in your findings. For instance, the aversive-selective domain exhibits up to 30% of significant coactivation voxels in the limbic network, whereas other domains show a maximum of approximately 5%. In other words, your data suggest that most domains are characterized by a specific network that is more pronounced in that domain. I think you do not need to be so restrictive in this

section—discussing the trends that are clearly evident in your data would further strengthen your results.

Additionally, Figure 4 shows that 10–20% of the significant coactivation voxels for each domain contribute to the visual network. However, from my point of view this is not clearly conveyed in the text or the figure. From my understanding, the visual network, as presented in the paper by Yeo et al., is primarily localized in the occipital lobe. Yet, in both the surface projection and the text, I do not find anatomical regions or voxels associated with that part of the brain. Given the substantial proportion of voxels across all domains contributing to the visual network, some activation should be visible in the surface projection. Could you clarify which anatomical structures you are referring to when showing up to 20% visual network participation?

(Remarks on code availability)

Reviewer #2

(Remarks to the Author)
This manuscript presents a large-scale mega-analysis of functional MRI data (n = 540 participants, 36 studies) to investigate the spatial organization of domain-general and domain-specific representations within the human insula. Using a voxel-wise Bayesian model selection approach, the authors identify a domain-general convergence zone located primarily in the dorsal anterior insula, surrounded by domain-selective territories for pain, appetitive, aversive, and cognitive processes. Supplementary analyses include meta-analytic decoding, coactivation mapping, and receptor profiling to characterize the identified regions.

While the general aim is timely and the dataset is substantial, I have significant concerns regarding key aspects of the study's anatomical definition, methodological rigor, statistical robustness, and the conceptual novelty of the findings. These issues, detailed below, raise important questions about the reliability and interpretability of the conclusions.

1.
The anatomical definition of the insula omits key opercular regions (OP3, OP5, OP7, and OP9) that straddle the superior limiting sulcus. This omission critically impacts the study's findings. OP3, in particular, corresponds to the dorsal posterior insula, a key hub for nociceptive processing (Craig, 2002; Segerdahl et al., 2015, Nature Neuroscience), and its exclusion likely accounts for the reported absence of a posterior pain-selective hotspot. Furthermore, excluding OP7 and OP9 could affect the interpretation of convergence and selectivity within the dorsal anterior insula. Given that the central conclusions rely on the spatial organization of functional domains, an anatomically comprehensive definition of the insula is essential. The authors should either redefine the insular mask to include these opercular areas and reanalyze the data, or explicitly justify their exclusion and discuss the implications for their findings.

2.
Even with anatomical corrections, the conclusions heavily rely on the consistency and validity of the datasets included in the mega-analysis. It is unclear how "pain" was defined across studies, including differences in stimulus type (thermal vs. mechanical), stimulus dynamics (single temperature vs. ramping), and experimental design (block vs. event-related). Such differences could significantly impact insular activation patterns and undermine the assumption of domain uniformity. Similar concerns apply to the appetitive, aversive, and cognitive control domains. It would strengthen the manuscript if the authors provided detailed information on task definitions and experimental designs across studies, and discussed how paradigm variability may limit the specificity of the reported domain organization.

3.
The insula exhibits considerable inter-individual variability in sulcal and gyral morphology (e.g., DOI 10.1007/s00429-010-0248-y), which can significantly affect the spatial localization of functional activations. The first short gyrus, in particular, varies notably between individuals. Given that the authors report a core domain-general focus on the first short gyrus surrounded by domain-selective regions, the reliance solely on normalized MNI-space analyses raises concerns about anatomical reproducibility. The authors should acknowledge the limitations inherent to template-based normalization and discuss how individual variability might influence the interpretation of domain convergence and selectivity. Validation of the reported organization in native anatomical space would be ideal to reinforce the robustness of their conclusions.

4.
The identification of domain-selective regions is central to the study's conclusions, but the domain-selective maps (Figure 2) appear small, patchy, and irregular, raising concerns about whether they represent meaningful functional specialization or statistical noise. Additionally, the claim of "largely symmetrical" organization across hemispheres is not fully supported by the data; for example, the cognitive domain (yellow) shows markedly different localization between the left and right insula. True biological specialization would be expected to produce more coherent and reproducible fields. The authors should critically examine the degree of asymmetry present in their results and discuss the potential impact of smoothing, statistical thresholding, and averaging across individuals on the observed patterns.

5.
While the use of a Bayesian framework is appropriate in principle, the pooling of beta maps across 36 studies without modeling "study" as a random effect ignores potential systematic variability between datasets, which could bias the voxel-level results. Furthermore, the Bayes Factor threshold applied (BF > 3) represents only modest evidence by conventional

standards, and no correction for multiple comparisons across voxels appears to have been implemented. These statistical choices could impact the robustness of the findings. A clearer discussion of these limitations, including the rationale behind the selected statistical thresholds and the potential influence of multiple comparison issues, would significantly strengthen the manuscript.

6.
The manuscript presents detailed voxel-wise maps of convergence and selectivity, but no validation procedures (e.g., cross-validation or permutation testing) were employed to assess the robustness of the spatial patterns. Given the complexity of the insula and the heterogeneity of the pooled datasets, validation of the observed structures is crucial to ensure they are not artifacts of sample composition or thresholding practices. The authors should explicitly acknowledge this limitation and consider implementing, or at least proposing, validation procedures in future work.

7.
While the mega-analysis approach is valuable, the finding that the anterior insula engages across multiple domains is well established in prior literature. Although the authors propose a refined spatial structure-a core of convergence surrounded by domain-selective zones-serious concerns about anatomical precision, domain construct validity, statistical robustness, and inter-individual variability call into question the reproducibility of this fine-grained organization. As such, it is unclear whether the manuscript delivers a sufficiently novel or robust advance beyond existing models of insular function.

(Remarks on code availability)

Reviewer #3

(Remarks to the Author)
Kwon and colleagues analyzed through a mega-analysis the profiles of insula activity related with different domains, with a focus on their degree of selectivity or overlap.

I first of all commend the authors for the great amount of work done, providing a complete picture by means of a wide range of analyses, bridging the macro with the micro scale.
The manuscript is in my opinion very well written and generally easy to follow.

My main concern pertains the number of considered studies.
I see that mega-analyses are "better powered" than coordinate-based meta-analyses, so this is not a matter of statistical soundness, but rather of representation of the cognitive domains. Are 9 studies (further divided across 3 sub-domains) enough to describe something as wide as cognitive control, or pain? I think this is even more relevant as the aim here is to think in terms of selectivity. If I think of other (more or less) recent works on selectivity analyses in MRI, the idea is to test the domain of interest against the widest possible picture of alternative cases.

Related to this, I’m not very familiar with the anic database, but it is not clear to me if more studies would have been available for each domain to be selected. If this was the case, I’m wondering whether the selection of different triplets would have changed the results a lot (something that could be tested through a bootstrap approach).

Following on the studies topic, why did the authors decide to include 2 experiments in the aversive domain (exp 26 and 27) being related to some extent with pain? Isn't this counterintuitive in a framework of specificity analysis?

Also, why did the authors decide to include data from several unpublished studies? This is generally discouraged in meta-analyses.

On a more methodological side, I could have got it wrong, but I’ve some reservation concerning the idea (expressed in the supplementary) that if one domain generated wider activation, normalization should be used to let other domains emerge. If it is the case that pain activates the whole insula, isn’t it exactly the kind of information that a similar paper is looking for?

A few minor points:

I very much liked the composite nature of figure 1, but some aspects were a bit confusing to me. First, the mentioning of SVM in the title was unexpected, as I hadn’t understood from the previous sections that any machine learning was performed. Second, I’m not very sure of what map is referred in point d of the caption. Is it that appearing in panel c? Or did the authors refer to the diagram in panel d, that in this case I would not define a map?

As commented on figure 1, I would consider to introduce SVM somewhere before the results section, since the methodological framework seems rather to be on mega-analyses and Bayesian statistics only.

At first read, it is not clear why 33.17% for aversive processes is not above-chance, that is previously set to 25%. I guess it depends on the p, but this could be maybe made more straightforward for readers.

I’m not a native English speaker, but the term “coherent” in the sentence "Overall, these findings indicate that three of the four domains show coherent insular representations" makes me think that the three domains are similar among them (while the details in brackets clarifies it is not so). I would suggest to consider to change “coherent” with a different synonym.

Based on this I would suggest minor revision of the manuscript before further consideration.

(Remarks on code availability)

Version 1:

Reviewer comments:

Reviewer #1

(Remarks to the Author)
The authors have addressed all of my previous revision concerns. I have no further comments.

(Remarks on code availability)

Reviewer #2

(Remarks to the Author)
I would like to thank the authors for the exceptionally thorough and careful revision. They have engaged seriously with the reviewers' comments, added substantial new analyses, clarified methodological choices, and significantly strengthened both the Results and Discussion. My original concerns have been addressed in a rather satisfactory and constructive manner, and the manuscript is substantially improved.

At an interpretive level, even if I personally remain a bit skeptic about the "map" generated here, I view the proposed model as a useful and stimulating framework, and I will be very interested to see how future studies refine, qualify, or challenge it using higher-resolution imaging, within-subject approaches, electrophysiology, or causal perturbations. Given the likely impact of this work, it would be important—especially in talks and pedagogical contexts—to present the model alongside its current limitations, so as to avoid it being interpreted as a definitive or exhaustive map. Historical precedent illustrates how simplified maps can unintentionally harden into dogma despite more complex underlying organization. (For example, Penfield's motor homunculus was often interpreted as a fine-grained musculotopy when Penfield himself stated that the inherent organization was more complex and when later studies showed that the motor cortex is made of complex overlapping re-representations of muscle groups likely supporting muscle synergies. Today, despite many demonstrations of the complexity of M1, readers of textbooks are often misled to see the map as musculotopic. Had Penfield emphasized his word of caution, maybe textbooks would have also carried that caution forward.)

I have one remaining, relatively minor conceptual point concerning terminology, specifically the use of the term "domain-selective". For example, in the case of pain, because posterior nociceptive signals re-emerge under alternative normalization schemes, and because anatomical and electrophysiological work demonstrates dense spinothalamocortical input to dorsal posterior insula, there is a risk that "pain-selective" could be read as implying exclusive nociceptive encoding or privileged afferent termination. Of course, pain per se is more than nociception but the terminology can still lead to confusion. This comment reminds me of the works by Mouraux and colleagues (e.g., https://pubmed.ncbi.nlm.nih.gov/26734726/; https://pubmed.ncbi.nlm.nih.gov/21040755/), showing that the insula and other so-called "pain matrix" regions are in fact similarly activated by any salient stimuli. In this sense, the issue is less about the data themselves than about what is implied by the terminology used to describe them. Finally, at the voxel level, fMRI "selectivity" should not be interpreted as homogeneous neuronal tuning, as individual voxels likely contain mixed neuronal populations responding to multiple interoceptive and exteroceptive features. I would therefore suggest brief clarifying statements—perhaps in the Results and Discussion—explicitly noting that "domain selectivity" refers to relative domain preference under the chosen normalization and analytic framework, rather than modality exclusivity, anatomical input specificity, or labeled-line coding. Alternatively, or in addition, slightly softening the terminology (e.g., "domain-preferential" or "relatively selective") could help pre-empt over-interpretation without altering the substance of the findings. The same applies of course for the other domains.

With these minor clarifications, I am satisfied with the revision and consider the manuscript suitable for publication.

(Remarks on code availability)

Reviewer #3

(Remarks to the Author)
I thank the authors for the time and effort dedicated to replying to my comments.
I have no further requests, and I therefore recommend to accept the paper for publication in the current form.

I congratulate the whole team.

(Remarks on code availability)

Open Access This Peer Review File is licensed under a Creative Commons Attribution 4.0 International License, which permits use, sharing, adaptation, distribution and reproduction in any medium or format, as long as you give appropriate credit to the original author(s) and the source, provide a link to the Creative Commons license, and indicate if changes were made.
In cases where reviewers are anonymous, credit should be given to 'Anonymous Referee' and the source.
The images or other third party material in this Peer Review File are included in the article's Creative Commons license, unless indicated otherwise in a credit line to the material. If material is not included in the article's Creative Commons license and your intended use is not permitted by statutory regulation or exceeds the permitted use, you will need to obtain permission directly from the copyright holder.
To view a copy of this license, visit https://creativecommons.org/licenses/by/4.0/

Response to Reviewers

We would like to thank the reviewers for their thoughtful and constructive feedback on our manuscript. Their comments have helped us strengthen and clarify our work in several important ways. Below, we provide detailed responses to each point raised and indicate the corresponding changes made to the manuscript. All changes are highlighted in blue in the revised manuscript and supplementary materials. In this response letter, we indicate revisions by providing page numbers for extensive changes and direct quotes for specific modifications.

Reviewer #1

Thank you for your positive assessment of our work and valuable suggestions regarding the cytoarchitectonic characterization of the insula.

Comment 1.1. Clarification of microstructural-related claims:

In both the methods and results sections, you state that the microstructural organization of the human insula consists of "16 areas grouped into four clusters (posterior, dorsal anterior, inferior posterior, and ventral anterior) based on cytoarchitectonic characteristics (granular-dysgranular, dysgranular, dysgranular-agranular, and agranular)." However, this statement regarding the clustering solution is not accurate. The cluster analysis we conducted in our paper was based on the areas available at the time and did not include the areas of the ventral anterior insula and certain parts of the agranular ventral posterior pole/Limen insulae. While these areas are publicly available, the publication, along with an overall cluster analysis, is still in preparation and not yet published. Therefore, there is currently no comprehensive clustering solution incorporating all cytoarchitectonic areas, and no evidence supports the existence of a fourth fully agranular cluster in the ventral anterior insula as implied in this statement. A similar issue arises in Figure 5, where you depict an agranular ventral anterior cluster. The greyed-out areas were not part of the cluster analysis and contain both agranular and dysgranular characteristics rather than being purely agranular.

Given that your analysis focuses on specific areas rather than a clustering solution and does not center on cytoarchitectonic details, I recommend omitting references to the clustering solution from the text and figure and instead referring directly to the areas.

Response 1.1. Thank you for the clarification. We have now removed references to the clustering solution and refer directly to the individual areas. Specifically, we have updated all references to use specific cytoarchitectonic areas (e.g., Id6, Id7) without using the four-cluster framework as suggested (pp. 14).

Comment 1.2. In that regard, I noted some further minor issues in the manuscript **that need clarification:**

- ***“Pain-selective zones were a notable exception to this pattern of cytoarchitectonic associations, showing a complex pattern spanning multiple cytoarchitectonic clusters that both aligns with and diverges from previous findings.” → Since a full cluster analysis covering all insular areas is not yet available, I suggest replacing "clusters" with "areas" to ensure accuracy.***

Response 1.2. We have made this change as follows:

“Pain-selective zones were a notable exception to this pattern of cytoarchitectonic associations, showing a complex pattern spanning multiple cytoarchitectonic areas that both aligns with and diverges from previous findings.” (pp. 20)

Comment 1.3.• *“Primarily in agranular insula, with appetitive-selective zones overlapping most strongly with the left inferior posterior cluster.”*

• “Aversive-selective zones in agranular regions (Id10) align with known limbic system projections and correspond to regions showing specific responses to negative emotional stimuli in electrophysiological recordings.”

→ The region you describe here is not agranular. Id10 stands for "Insular dysgranular area 10"; therefore, it is not agranular.

Response 1.3. Thank you for catching this error. We have corrected it by refining terminology to correctly refer to Id10 as dysgranular, not agranular:

“Aversive-selective zones in area Id10 align with known limbic system projections and correspond to regions showing specific responses to negative emotional stimuli in electrophysiological recordings.” (pp. 20)

Comment 1.4 *You state in the discussion that “Id6 showed hypervigilance and anxiety when stimulated.”*

→ While this is partially true, as mentioned in our paper, the majority of findings in this area indicate non-responsiveness or visceral sensations. Please revise this statement to be more precise, such as: “Id6 showed hypervigilance and anxiety in a minor subset of patients when stimulated.”

Response 1.4. Thank you, we have revised the paper to make the suggested change. Specifically, we have clarified that stimulation of Id6 produced hypervigilance and anxiety in a small subset of patients as well as non-responsiveness or visceral sensations as follows:

“Id6 showed primarily visceral sensations with anxiety/hypervigilance in a small subset of cases.” (pp. 20)

Comment 1.5. *Further, you write: “As with resting-state networks, our functional insular zones mapped onto cytoarchitecture to some degree, but cytoarchitectonic boundaries*

were not sufficient to describe them completely." → Please note that your analysis utilized maximum probability maps, assigning each voxel to the area with the highest probability. As a result, activations near the borders between two areas may be difficult to interpret because there is still some probability that this activation is located within the neighboring area. Just be aware of the probabilistic nature of the microstructural maps. You might want to acknowledge this limitation in the cytoarchitectonic-related discussion part or limitations section.

Response 1.5. We have now noted that the analysis used maximum probability maps, and mentioned this caveat in the results and discussion. We have added acknowledgment of the probabilistic nature of cytoarchitectonic maps in the limitations section as follows:

"Fifth, our cytoarchitectonic mapping relied on maximum probability maps from the Julich-Brain atlas, where each voxel is assigned to the area with the highest probability. This approach simplifies the inherent uncertainty in these assignments, particularly at borders between areas. Future studies could address this by using probabilistic approaches that preserve uncertainty information or by examining structure-function relationships at the individual subject level. We hope our study inspires future work to expand this approach and address these outstanding long-term goals." (pp. 23).

Comment 1.6. References:
In all instances where you cited the microstructural insula atlas, such as in the statement:
"To distinguish between these possibilities and better understand the nature of these zones, we further characterized them through multiple levels of analysis: meta-analytic functional decoding (using Neurosynth19,42), coactivation with other brain regions66,67, cytoarchitectonic mapping23,68, and neurotransmitter system profiling69,70."
Please also include the work of Kurth, Grodzinsky, and Hein, as they all contributed to the insula microstructural atlas. The relevant references can be found in the caption of Figure 13 in our paper, "Cytoarchitecture, probability maps, and segregation of the human insula."

Response 1.6. We have now cited work from Grodzinsky et al., (2020), and work from Hein et al., (2021c, 2021b, 2021a) on the microstructural atlas of the insula, in addition to Kurth et al., (2010) as recommended (pp. 3).

Comment 1.7. Furthermore, as you have discussed Kurth's work, "A link between the systems: functional differentiation and integration within the human insula revealed by meta-analysis," in detail in the introduction, it would be beneficial to briefly mention and compare his findings with your results in the discussion section. Since his results regarding a convergence zone are localized in a region comparable to your findings, such a comparison would further support your results and provide a clearer conceptual link between the arguments presented in the introduction and discussion.

Response 1.7. We have now added a paragraph comparing our findings with Kurth et al. (2010) as follows:

"Our findings both confirm and extend Kurth et al.'s seminal meta-analysis[4] of insular function. While Kurth et al. identified partial overlap across domains in anterior-dorsal insula using coordinate-based methods, our image-based mega-analysis provides greater spatial precision, localizing this convergence to specific regions in bilateral dorsal anterior insula. Furthermore, our approach enables us to differentiate domain-general from domain-selective zones and reveal a functional gradient from specialized processing in posterior/ventral regions to multi-domain integration in dorsal anterior insula—a nuanced organization not fully captured in previous work." (pp. 18)

Comment 1.8. Additionally, I suggest two more papers that directly link area Id7 with specific functions that should be at least cited or in the case of Sabat at al. briefly discussed in the discussion section:
M. Sabat, C. de Dampierre, & C. Tallon-Baudry, Evidence for domain-general arousal from semantic and neuroimaging meta-analyses reconciles opposing views on arousal, Proc. Natl. Acad. Sci. U.S.A. 122(6), e2413808122, https://doi.org/10.1073/pnas.2413808122 (2025).
Grodzinsky, Y., Deschamps, I., Pieperhoff, P. et al., Logical negation mapped onto the brain, Brain Struct Funct 225, 19–31 (2020). https://doi.org/10.1007/s00429-019-01975-w

Response 1.8. We have now cited Sabat et al. (2025) and Grodzinsky et al. (2020) on Id7 functions and included discussion on the results of Sabat et al. (2025) as follows:

"Domain-general and cognitive-selective zones were predominantly located in dysgranular cortex (Id6 and Id7, respectively), aligning with recent findings suggesting that dysgranular dAIns is a key region for convergence and cognitive control[20,98,120–127]. For example, Sabat et al. (2025) identified a domain-general arousal hub in similar cytoarchitectonic regions to our cross-domain convergence zone, centered in Id7 but extending into Id6 and Id8." (pp. 20)

Comment 1.9. Finally, in the discussion, you state:
"Specifically, we did not observe pain-selective activity in Ig1 (bilateral), Ig2 (left), and Id2 (left) of the dorsal posterior insula—regions where many previous studies found pain-selective activity54,55,118 and where direct stimulation reliably evokes pain29,56,119–121."
To the best of my knowledge, none of these studies directly associated their findings with microstructural areas, except for our paper, "Subjective states induced by intracranial electrical stimulation match the cytoarchitectonic organization of the human insula." Therefore, your claim here appears speculative and lacks direct supporting evidence. I recommend rephrasing this statement as:"In an anatomically comparable localization, many previous studies have reported pain-selective activity.

Response 1.9. We have now refined our statement about pain-selective activity in specific regions to clarify that previous studies reported pain-selective activity in the dorsal posterior insula without specifying cytoarchitectonic regions as follows:

"*Specifically, we did not observe pain-selective activity in Ig1 (bilateral), Ig2 (left), and Id2 (left) of dorsal posterior insula—regions anatomically comparable to those where many previous studies found pain-selective activity*[54,55,132]" (pp. 20).

Comment 1.10. 3. discussing network results: In the discussion, you state:
"With respect to canonical resting-state cortical networks, domain-general and domain-selective insular zones and their coactivated systems were unevenly distributed across the networks, but did not align in a one-to-one fashion with them."
While this statement is profound based on your data, I believe there are some noteworthy trends in your findings. For instance, the aversive-selective domain exhibits up to 30% of significant coactivation voxels in the limbic network, whereas other domains show a maximum of approximately 5%. In other words, your data suggest that most domains are characterized by a specific network that is more pronounced in that domain. I think you do not need to be so restrictive in this section—discussing the trends that are clearly evident in your data would further strengthen your results.

Response 1.10*.* Thank you for this observation. We have expanded our discussion to highlight specific network associations, including the aversive-selective domain showing particularly strong limbic network affiliation compared to other domains.

The revised discussion now acknowledges that while insular zones showed characteristic network affiliations, including the aversive-selective domain's particularly strong limbic network representation (>40% of coactivated voxels), these associations spanned multiple networks rather than aligning one-to-one. Specifically, we note that aversive zones affiliate with both default and limbic networks, pain and appetitive zones with somatomotor and ventral attention networks, cognitive zones with frontoparietal and dorsal attention networks, and domain-general zones with frontoparietal, ventral attention, and default networks. The changes can be found in Results "Coactivation between insular zones and other brain systems" (pp. 12-13).

This multi-network spanning positions insular zones at strategic intersection points between canonical networks, potentially facilitating flexible inter-network coordination based on psychological demands. Rather than being constrained within single networks, these zones appear optimally positioned to serve as integration hubs, a property consistent with previous findings that affective decoding models span multiple resting-state networks. The revision has been made in the Discussion as follows:

"*With respect to canonical resting-state cortical networks, domain-general and domain-selective insular zones and their coactivated systems showed distinctive distribution patterns. While notable network preferences emerged—with each domain showing stronger affiliation with specific network subsets: aversive with default and limbic networks (with limbic comprising >30% of coactivated voxels), pain and appetitive processes with somatomotor and ventral*

attention, cognitive with frontoparietal and dorsal attention, and domain-general with frontoparietal and default networks—their associations spanned multiple networks and did not align in a one-to-one fashion. This property is consistent with previous findings that decoding models predicting affective experiences span multiple resting-state networks[106–109]. On one hand, resting-state networks may be related to task-based patterns at a coarse level[110] and predictive of individual task-evoked topography[111], but on the other, tasks evoke reorganization of functional connections and networks in ways that cannot be captured by static resting-state patterns[112–114], including alterations of brain-wide neuronal activity patterns by stimuli and activation of neuromodulatory nuclei[115–119]. This may help explain why the insular zones and coactivated systems we identify here are not reducible to simple combinations of resting-state networks. Instead, activations arise from the bridging of multiple networks." (pp. 19-20)

Comment 1.11. Additionally, Figure 4 shows that 10–20% of the significant coactivation voxels for each domain contribute to the visual network. However, from my point of view this is not clearly conveyed in the text or the figure. From my understanding, the visual network, as presented in the paper by Yeo et al., is primarily localized in the occipital lobe. Yet, in both the surface projection and the text, I do not find anatomical regions or voxels associated with that part of the brain. Given the substantial proportion of voxels across all domains contributing to the visual network, some activation should be visible in the surface projection. Could you clarify which anatomical structures you are referring to when showing up to 20% visual network participation?

Response 1.11. We have now clarified our methodological approach for the resting-state network analysis. Our original analysis used unthresholded coactivation maps to provide a more comprehensive and unbiased picture of network affiliations by using the full coactivation patterns rather than analyzing only the peak coactivations that survive our stringent thresholding. However, for consistency with other coactivation analyses in the manuscript (which use thresholded maps at FDR q < 0.001), we have now placed the thresholded network analysis in the main results (Fig. 5b; attached below) and moved the unthresholded analysis to Supplementary Fig. 13). Both analyses show that primary network associations remain consistent, with differences mainly in the extent of visual network representation. The visual network associations noted by the reviewer appear in the unthresholded analysis but are largely absent in the thresholded analysis, explaining their absence from the surface projections. Corresponding revisions have been made as follows:

Results - Fig. 5b:

"*Distribution of coactivated regions across seven resting-state networks[84–86]. Bar graph shows the percentage of coactivated voxels (FDR q<0.001) overlapping with each network across cortical, subcortical, and brainstem regions.*" (pp. 14)

Methods:

"*The resulting correlations were thresholded at FDR-corrected q<0.001 and cluster size>100 voxels. Voxels above the threshold were assigned to one of the insular zones based on which*

zone showed the maximum correlation, provided that the maximum correlation with one of the insular zones was at least 10% higher than the next highest correlation.

To examine the relationship between the coactivated regions and seven resting-state networks (combined from refs.[84–86]), we calculated the percentage of voxels in the thresholded coactivated regions that overlapped with each of the seven resting-state networks in the cortical, subcortical, and cerebellar regions. A supplementary analysis using unthresholded coactivation maps is provided in Supplementary Fig. 13." (pp. 28)

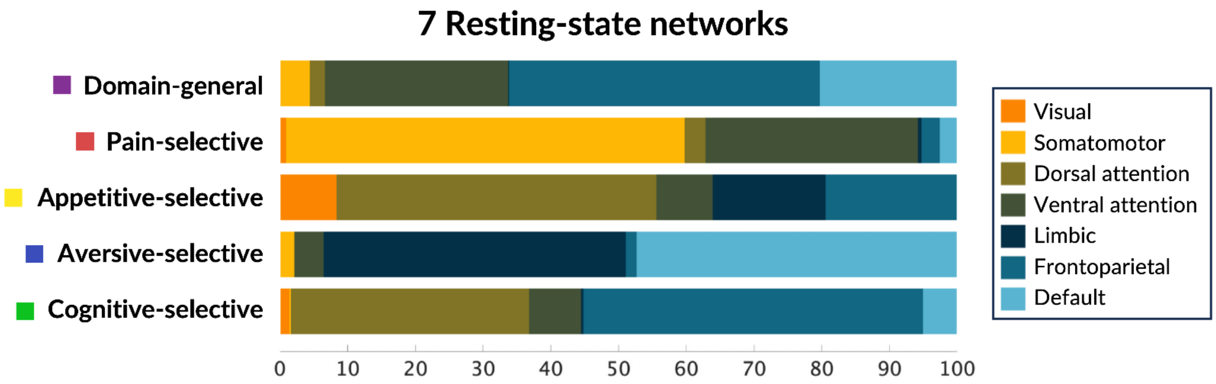

Reviewer #2

We thank Reviewer #2 for their thorough assessment of our manuscript and appreciate their constructive feedback, which has helped us improve the clarity and robustness of our findings.

Comment 2.1. The anatomical definition of the insula omits key opercular regions (OP3, OP5, OP7, and OP9) that straddle the superior limiting sulcus. This omission critically impacts the study's findings. OP3, in particular, corresponds to the dorsal posterior insula, a key hub for nociceptive processing (Craig, 2002; Segerdahl et al., 2015, Nature Neuroscience), and its exclusion likely accounts for the reported absence of a posterior pain-selective hotspot. Furthermore, excluding OP7 and OP9 could affect the interpretation of convergence and selectivity within the dorsal anterior insula. Given that the central conclusions rely on the spatial organization of functional domains, an anatomically comprehensive definition of the insula is essential. The authors should either redefine the insular mask to include these opercular areas and reanalyze the data, or explicitly justify their exclusion and discuss the implications for their findings.

Response 2.1. We appreciate this thoughtful observation regarding anatomical boundaries between insular and opercular regions. To comprehensively address this consideration, we reviewed relevant literature including the references that the reviewer cited and conducted supplementary analyses with expanded anatomical definitions.

The literature indicates that opercular regions are functionally integrated with the insula while remaining anatomically distinct structures. For example, the cited references consistently describe the posterior insular and adjoining medial operculum as anatomically separate regions that form a functional unit (Garcia-Larrea et al., 2010; Segerdahl et al., 2015). Craig (2002) clarifies that while historical atlases incorrectly grouped posterior insular regions with parietal operculum, these remain distinct structures despite their functional integration. For pain processing specifically, this anatomical distinction is supported by evidence of two separate pain representations in the operculo-insular complex, one in the parietal operculum and another in the insular cortex (Mazzola et al., 2006).

Notably, recent tractography evidence (n=1118 in total) demonstrated that the strongest connectivity across the entire insula connectome occurs with adjacent opercula via extreme capsule fibers, with specific insular clusters maintaining preferential connections to distinct opercular areas (Quabs et al., 2025). This suggests that if functional zones extend beyond the insula, they should appear selectively in opercular regions with corresponding structural connections rather than randomly.

To test this hypothesis and address the reviewer's suggestion, we conducted extended analyses using expanded masks including the suggested opercular regions defined based on two atlases: (1) parcels from the Julich cytoarchitectonic atlas, including both insular regions and the suggested opercular parcels from Quabs et al. (2022), (2) all 13 parcels from the insular-opercular group in the HCP-MMP1.0 atlas (Glasser et al., 2016), using their frontal

opercular parcels (FOP2-FOP5), as Glasser et al. did not provide direct correspondence to OP3/5/7/9.

The expanded analyses revealed that, in addition to the insular findings that are the primary focus here, functional zones extend into specific opercular regions in patterns consistent with the structural connectivity architecture described by Quabs et al. (2025). We now report these results in the Results (pp. 9), Methods (pp. 26-27; “Anatomical definition of insula”), Supplementary Methods (pp. 2, “Supplementary analysis with expanded insula boundaries”), and Supplementary Fig. 7. To summarize, pain-selective zones appeared predominantly in OP5 (33% right, 16% left in Julich), with limited representation in OP3 (12.3% right in Julich). Domain-general zones extended into OP7 and OP9 (Julich) precisely the opercular areas with strongest connectivity to dorsal anterior insular regions (Quabs et al., 2025). These findings demonstrate how functional properties extend selectively into anatomically adjacent but distinct structures based on their connectivity patterns.

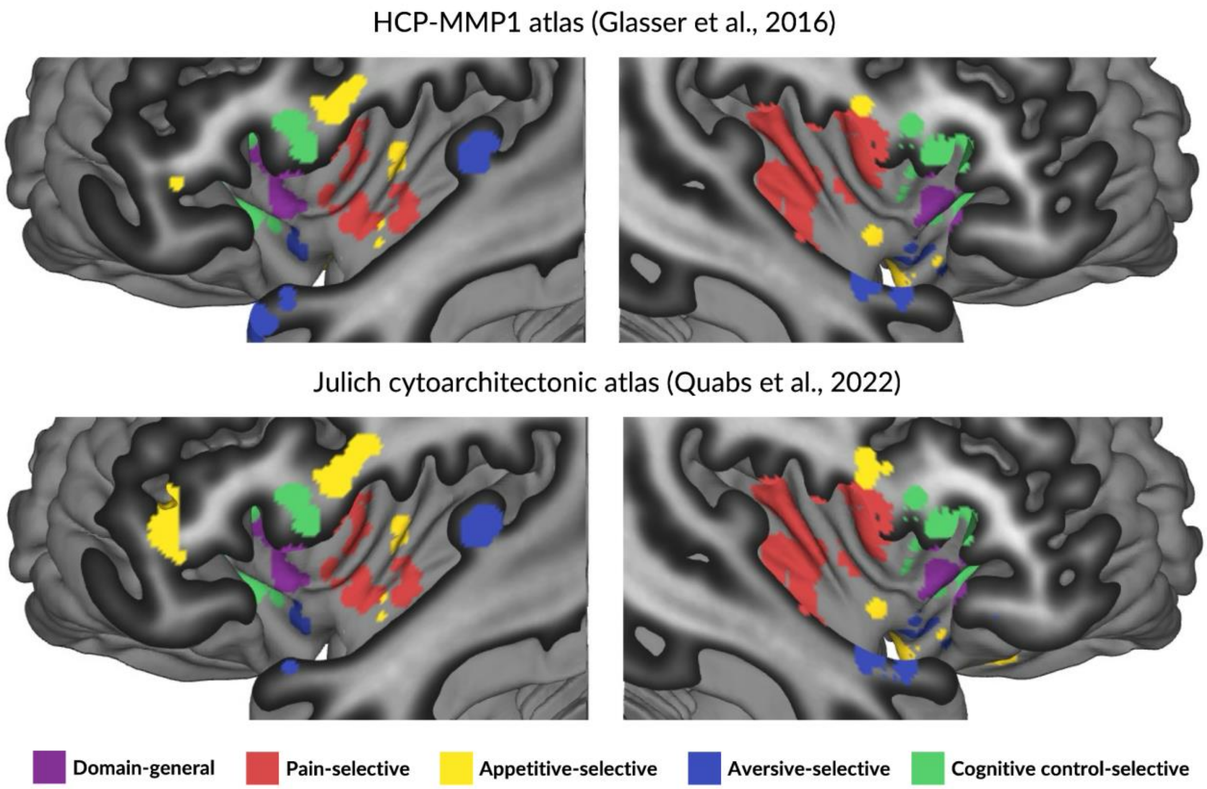

Despite including OP3, we did not observe the strong posterior pain-selective hotspot the reviewer anticipated. This likely reflects our two-stage analysis that examines both global insula activation and normalized activation patterns. We now report global insula activation in the Results (pp. 5; “Global insular activation across domains”; see also Response 3.3), which shows that pain produces higher overall activation across the insula, explaining the absence of additional pain-selective findings in posterior insula and OP3 regions (also see Discussion on pp. 20-21). Importantly, when we applied alternative normalization methods that preserve global activation patterns (L1 and L2 normalizations), we do observe pain-selective regions in the

posterior insula/operculum, confirming these signals are present but obscured by our primary analytical approach (now included in the Supplementary Fig. 6, reproduced here).

Pain-selective zones with L2 normalization

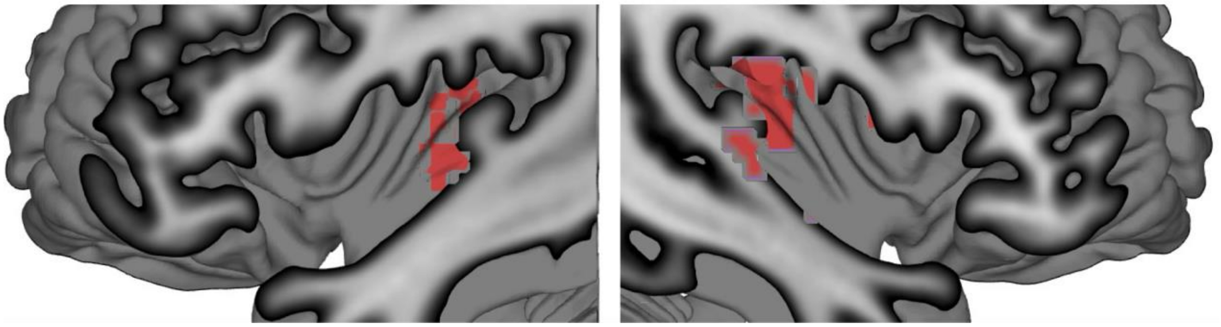

Additionally, we now acknowledge that lateralization effects and somatotopy in posterior insula may produce variability in pain representation that may obscure findings of pain specificity, as our dataset includes right-sided, left-sided, and midline/visceral stimulation (pp. 21).

We appreciate that many readers would like to see results in opercular regions as well. To balance this interest with the main focus of the paper on the insula proper, we have added the additional results including the operculum in Supplementary Fig. 7.

Comment 2.2. Even with anatomical corrections, the conclusions heavily rely on the consistency and validity of the datasets included in the mega-analysis. It is unclear how "pain" was defined across studies, including differences in stimulus type (thermal vs. mechanical), stimulus dynamics (single temperature vs. ramping), and experimental design (block vs. event-related). Such differences could significantly impact insular activation patterns and undermine the assumption of domain uniformity. Similar concerns apply to the appetitive, aversive, and cognitive control domains. It would strengthen the manuscript if the authors provided detailed information on task definitions and experimental designs across studies, and discussed how paradigm variability may limit the specificity of the reported domain organization.

Response 2.2. We agree with the reviewer that the details of task design could, in principle, affect the functional results from the constituent studies and thus the functional selectivity in the aggregate meta-analysis. Individual person-level data are not available for enough studies to systematically analyze variations in details of timing (e.g., stimulus ramps) and other design details. Comprehensively addressing this issue would require systematic sharing of person-level activation maps across the field, similar to the standard reporting of study-level coordinates in tables and recent efforts to publish study-level maps. In addition, in our assessment, studies in the field do not vary and compare all of these designs systematically, crossing and comparing stimulus types, ramp rates, durations, and other variables, so comprehensively addressing this problem is a fundamental challenge for the field. We now more clearly acknowledge the inherent limitations of the current dataset and provide suggestions for future directions on both points in the Discussion on as follows:

"*Second, we acknowledge that more comprehensive sampling of each domain would be ideal, and it would be helpful to test the generality and functional specificity in different sets of studies as person-level data become available. We have partially addressed this through validation with independent datasets (n=608), which confirmed the robustness of our findings. Beyond this, with enough studies with person-level data, future studies could even test studies within subdomains as random effects. Such comprehensive datasets with systematically coded methodological variations would also shed light on how specific methodological variables impact activation patterns. Both of these advances, however, are beyond the scope of what is available and would require systematic sharing of person-level activation maps across the field, similar to the standard reporting of study-level coordinates in tables and recent efforts to publish study-level maps.*" (pp.22)

In addition, we have taken the reviewer's suggestion and updated Supplementary Table 4 to provide additional detailed information on specific task definitions, experimental designs, and data acquisition parameters for each included study.

There are several factors in our study that mitigate some of the concerns about design details, despite the limitations currently inherent in the field. Our systematic sampling approach requires that domain-level effects must generalize across different experimental implementations to be identified in our analysis. Any effects specific to particular paradigms or study-specific idiosyncrasies would not be identified as domain-general or domain-selective in our approach. Our approach was explicitly designed to test domain-level representations that generalize across different experimental paradigms: "*This design enabled us to test whether insular subregions encode domain information in a generalizable way across studies, which is critical for construct validation. For example, general pain-encoding regions should consistently respond to different types of pain (e.g., thermal, mechanical, and visceral pain), while not responding to non-nociceptive perturbations (e.g., emotionally arousing stimuli)*" (pp. 3). Our approach of including multiple studies per subdomain was specifically chosen to address the challenge of generalizing beyond single experimental paradigms. This methodology allows us to identify representations that are robust across different implementations of the same construct.

The example raised by the reviewer concerning pain is a useful example here. We note that in our 20 years of work on pain studies using different design variants, we do observe highly consistent findings in the insula across design variants including stimulus ramp rate, inter-stimulus intervals, and duration. However, we do see meaningful differences based on stimulus type (Čeko et al., 2022), with thermal stimuli producing stronger activation than mechanical (e.g., thumb pressure) in the insula, and vice versa for S1. We constructed the sample of studies here to include three studies of thermal pain, three studies of pressure pain, and three studies of visceral pain (rectal balloon inflation), and it is of interest to us that both domain-general and pain-domain-selective regions responded to all of these types of painful stimuli. The consistency of domain-level patterns despite the variability in lower-level design parameters, now documented more completely in Supplementary Table 4 following your excellent suggestion, strengthens the validity of our findings.

Comment 2.3. The insula exhibits considerable inter-individual variability in sulcal and gyral morphology (e.g., DOI 10.1007/s00429-010-0248-y), which can significantly affect the spatial localization of functional activations. The first short gyrus, in particular, varies notably between individuals. Given that the authors report a core domain-general focus on the first short gyrus surrounded by domain-selective regions, the reliance solely on normalized MNI-space analyses raises concerns about anatomical reproducibility. The authors should acknowledge the limitations inherent to template-based normalization and discuss how individual variability might influence the interpretation of domain convergence and selectivity. Validation of the reported organization in native anatomical space would be ideal to reinforce the robustness of their conclusions.

Response 2.3. Thank you for raising this important point about anatomical variability. We agree that our study, by its design focusing on group analyses across individuals and studies that underwent template-based normalization, cannot speak to whether domain-general regions fall largely on the first short gyrus at the individual level. We also agree that validation in native anatomical space would be ideal, and suggest that future precision neuroimaging studies could further test the anatomical reproducibility of our findings at the individual level. We now acknowledge these points explicitly in the Discussion as follows:

"*Fourth, we acknowledge that the insula exhibits considerable inter-individual variability in sulcal and gyral morphology. Our analyses identify group-level organization using template-based normalization to MNI space, which provides mapping of function onto anatomical features (gyri and sulci) at the group level. Future studies using precision neuroimaging approaches with multiple tasks within-person could examine how anatomical variability might influence functional localization and how closely domain-general and domain-selective regions map onto individual anatomical features.*" (pp. 23)

Comment 2.4. The identification of domain-selective regions is central to the study's conclusions, but the domain-selective maps (Figure 2) appear small, patchy, and irregular, raising concerns about whether they represent meaningful functional specialization or statistical noise. Additionally, the claim of "largely symmetrical" organization across hemispheres is not fully supported by the data; for example, the cognitive domain (yellow) shows markedly different localization between the left and right insula. True biological specialization would be expected to produce more coherent and reproducible fields. The authors should critically examine the degree of asymmetry present in their results and discuss the potential impact of smoothing, statistical thresholding, and averaging across individuals on the observed patterns.

Response 2.4. Thank you for challenging us to reconsider the spatial characteristics of our findings and similarity across hemispheres, and the match with the spatial characteristics that would be expected based on other literature. After considering the reviewer's comment and additional review of the literature, we suggest that rather than representing statistical noise, the "small, patchy, and irregular" appearance reveals important principles of insular organization that converge with findings from comparative neuroanatomy. Many individual fMRI studies use spatial smoothing and cluster-based thresholding, which is necessary to meet statistical

assumptions in some cases (e.g., when using Gaussian random field-based p-value corrections), but also result in artificially smooth-looking blobs, which are often interpreted as expected spatial characteristics. However, work on primate neurophysiology, and recent precision functional mapping studies in humans, reveal more patchy, irregular, and sometimes dimorphic patterns across cerebral hemispheres (e.g., Krockenberger et al., 2023; discussed below), and work on ectopic variants in functional connectivity (Dworetsky et al., 2024). Work on hemispheric lateralization over the past decades also shows important lateralization differences in some functions, including in the insula; for example, lateralization of autonomic functions has been reported in rodents and humans (Oppenheimer & Cechetto, 2016; Oppenheimer et al., 1992). Below, we show in new analyses and visualizations the approximate bilateral symmetry and robustness to thresholding of our domain maps.

Convergent evidence for patchy insular organization

The patchy organization we observe aligns well with high-resolution tract-tracing studies in non-human primates. Krockenberger and colleagues (Krockenberger et al., 2023) demonstrated that connections between prefrontal/cingulate cortex and macaque insula consistently form “sharply delimited patches” and “stripes” that precisely coincide with cytoarchitectonic boundaries. Analyzing five macaque monkeys, they found that different cortical areas (cingulate area 24b', orbital prefrontal areas 13L and 11L) each project to specific sets of insular patches rather than broad territories. Importantly, they showed this patchy organization was visible in neuroanatomical studies dating back decades (Burton et al., 1995; Mesulam & Mufson, 1982; Selemon & Goldman-Rakic, 1988) but had been overlooked. This convergent evidence across species, methods, and laboratories spanning 40 years strongly suggests that modular, patchy organization is a fundamental feature of insular architecture. We have added the following point to the Discussion:

“*The patchy organization we observe aligns with high-resolution tract-tracing studies in non-human primates. Recent tract-tracing studies have demonstrated that connections between prefrontal/cingulate cortex and macaque insula consistently form ‘sharply delimited patches’ that precisely coincide with cytoarchitectonic boundaries*[98]*. This patchy organization, visible across neuroanatomical studies spanning decades*[1,99,100] *but had been overlooked, suggesting that modular, patchy organization is a fundamental feature of insular architecture rather than statistical noise.*” (pp. 19)

A novel hierarchical organization principle in the insula

Our findings reveal an important organizational principle that reconciles seemingly contradictory views of insular organization. While we observe discrete, bounded patches at a local scale, these patches are not randomly distributed. Instead, they follow a systematic spatial arrangement from domain-selective regions in peripheral areas to multi-domain convergence in dorsal anterior insula. This provides empirical evidence for theoretical frameworks of convergence zones (Damasio, 1989; Simmons & Barsalou, 2003) and extends beyond the posterior-to-anterior progression previously proposed for the insula (Craig, 2002, 2009) by revealing convergence from both ventral and posterior regions. This represents a functional

convergence gradient, from specialized to integrated processing, distinct from the smooth cytoarchitectonic gradients that Evrard and others (Evrard, 2019; Evrard et al., 2014; Krockenberger et al., 2023) have challenged. This hierarchical organization, with discrete modules embedded within a global gradient of functional convergence, parallels known principles in other cortical systems (e.g., discrete columns within hierarchical visual areas) and likely reflects the insula's dual requirement to maintain distinct information streams while enabling their integration.

Validation with independent datasets

We validated our insular zones with four independent datasets (n=608 total) not included in our primary analysis, which confirmed that these seemingly "small, patchy" zones are not statistical artifacts but show robust domain-general and domain-selective activation properties even in unseen data. The validation results are detailed in Response 2.6.

Methodological considerations supporting genuine organization

Several methodological factors strengthen confidence that we are observing real biological organization:

1. Our voxel-wise Bayes Factor maps preserve native spatial resolution without cluster-extent thresholding that might artificially create more "coherent" regions by eliminating smaller activations.
2. We applied stringent dual criteria requiring both evidence for activation in one domain (BF > 4.32) AND evidence for null effects (BF < 0.23) in all other domains, thresholds equivalent to FDR-corrected q<0.01. This is much stricter than typical single-contrast analyses.
3. We examined domain-selective maps at multiple Bayes Factor thresholds (BF > 2, 3, 4, 4.32; Supplementary Fig. 12; reproduced below) and demonstrated that core organizational features persist across thresholds while boundaries refine, confirming these patterns represent genuine functional organization rather than statistical artifacts.
4. Individual variability in the precise location of functional regions, whether due to anatomical differences or other factors, can result in more conservative group-level maps where only voxels showing consistent evidence across variable individual topographies survive statistical thresholding. This makes our positive findings particularly notable.

Regarding hemispheric asymmetry, we have revised our hemispheric descriptions to more accurately reflect the patterns in our data with detailed information in Supplementary Table 1:

"*As shown in Fig. 3a, we identified specific regions within dAIns bilaterally that were activated across all domains and were therefore classified as domain-general (purple). These zones included 120 of 6012 insular voxels with relatively balanced bilateral distribution and spanned anterior short and inferior gyri[77]. This part of dAIns maps onto regions most frequently activated across different task domains in the Neurosynth database42 and is substantially higher in the*

principal cortical gradient identified by Margulies et al.[78]*, indicating transmodal (as opposed to unimodal) function. We also identified zones selective to each domain. Pain-selective zones (320 voxels, red in Fig. 3a) included voxels clustered in mid-posterior insula bilaterally, spanning from middle short to posterior long gyrus. Appetitive-selective zones (75 voxels, yellow) were proximal to pain-selective zones in left dorsal and bilateral ventral mid-insula, spanning anterior and posterior long gyri. Aversive-selective zones (142 voxels, blue) were primarily located in the ventral-most portion of bilateral agranular insula, on anterior inferior gyrus. Cognitive-selective zones (142 voxels, green) were predominantly located in the anterior-most portion of dAIns and most dorsal frontal opercular border bilaterally and spanned anterior and middle short gyri. These domain-selective zones showed preferential activations for each given domain and different patterns of hemispheric asymmetry (Supplementary Fig. 6). These activation patterns were consistent across subdomains and studies with few exceptions. Furthermore, these zones displayed distinct patterns of hemispheric asymmetry (e.g., pain and cognitive control show stronger right hemispheric asymmetry; see Supplementary Table 1 for further information on each insular zone).*" (pp. 7-8)

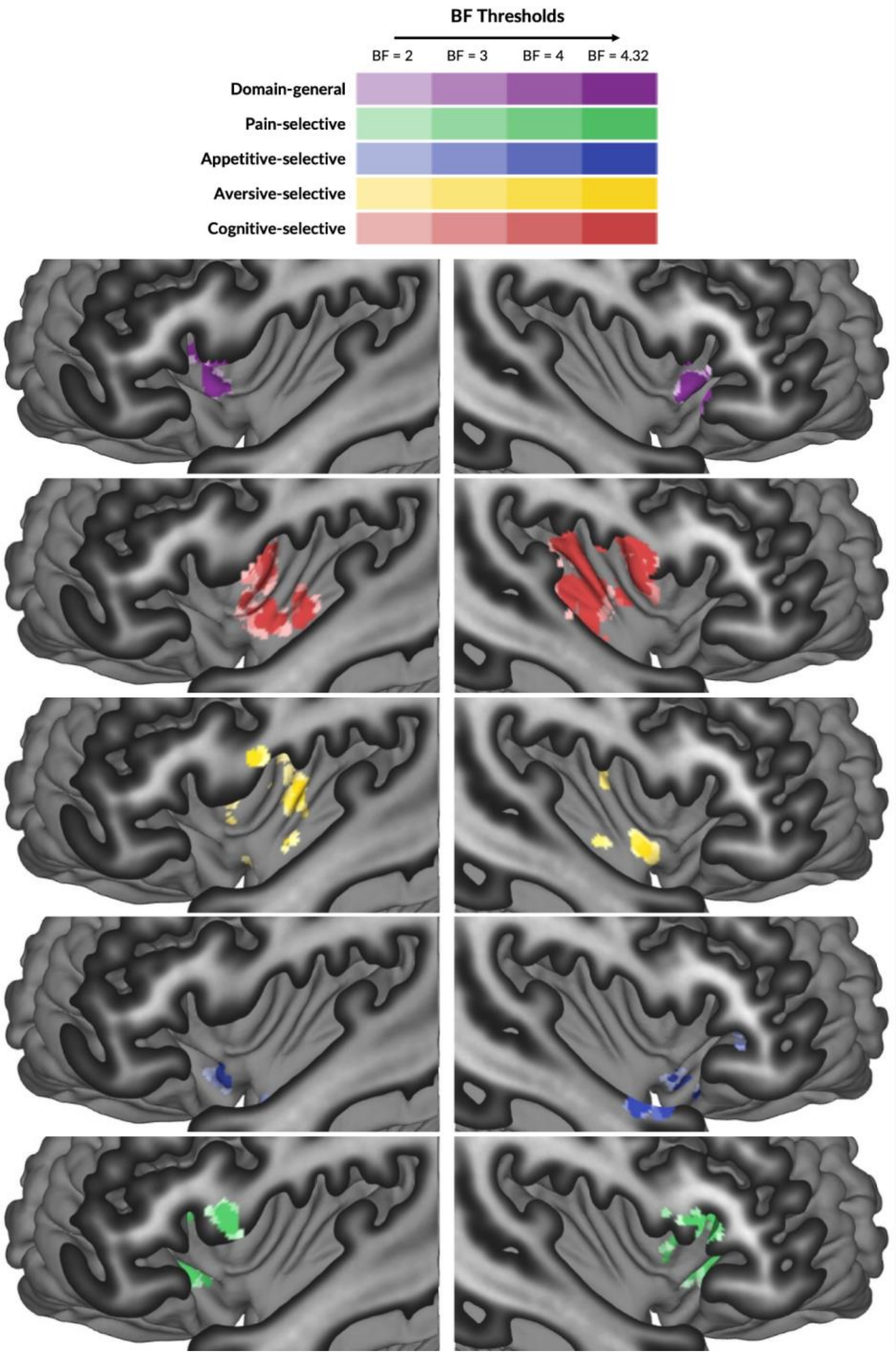

Comment 2.5. While the use of a Bayesian framework is appropriate in principle, the pooling of beta maps across 36 studies without modeling "study" as a random effect ignores potential systematic variability between datasets, which could bias the voxel-level results. Furthermore, the Bayes Factor threshold applied (BF > 3) represents only modest evidence by conventional standards, and no correction for multiple comparisons across voxels appears to have been implemented. These statistical choices could impact the robustness of the findings. A clearer discussion of these limitations, including the

rationale behind the selected statistical thresholds and the potential influence of multiple comparison issues, would significantly strengthen the manuscript.

Response 2.5. We thank the reviewer for raising these important statistical considerations and appreciate the opportunity to clarify our analytical approach.

First, we note that our analysis used a Bayes Factor threshold of 4.32 (not 3 as stated in the comment), which corresponds to q < 0.01 FDR correction. While this represents moderate evidence by traditional Bayesian standards, it is relatively conservative for neuroimaging studies. This ensures our findings meet conventional standards for controlling false discoveries while leveraging the advantages of Bayesian inference.

Regarding the concern about not modeling study as a random effect, we are sensitive to the fact that doing so would be advantageous, but this would require a very large sample of studies for each subdomain for sufficient power (we estimate 30 studies per subdomain, considering the present study's goals of establishing specificity within and generalizability across subdomains), and there are not enough studies to date for some subdomains to achieve this goal. In addition, there is insufficient infrastructure for data sharing to obtain person-level activation maps for these studies. Thus, here we have chosen to leverage the considerable power of using full activation maps for individual people and systematic, stratified sampling of psychological subdomains. Our revised discussion more clearly acknowledges replicating our findings in larger samples of studies as they become available would provide a viable alternative to the study-as-random-effect approach for generalizing to new, unobserved studies and individuals:

"*First, the current study selected a Bayes Factor threshold of 4.32:1, which corresponds to q < 0.01 FDR correction but provides moderate evidence by Bayesian standards, to balance the need for sufficient evidence with practical considerations in detecting effects in neuroimaging data. Future studies with larger sample sizes could employ higher thresholds to identify effects with even stronger evidence.*" (pp. 22)

"*Second, we acknowledge that more comprehensive sampling of each domain would be ideal, and it would be helpful to test the generality and functional specificity in different sets of studies as person-level data become available. We have partially addressed this through validation with independent datasets (n=608), which confirmed the robustness of our findings. Beyond this, with enough studies with person-level data, future studies could even test studies within subdomains as random effects. Such comprehensive datasets with systematically coded methodological variations would also shed light on how specific methodological variables impact activation patterns. Both of these advances, however, are beyond the scope of what is available and would require systematic sharing of person-level activation maps across the field, similar to the standard reporting of study-level coordinates in tables and recent efforts to publish study-level maps.*" (pp. 22)

Comment 2.6. The manuscript presents detailed voxel-wise maps of convergence and selectivity, but no validation procedures (e.g., cross-validation or permutation testing) were employed to assess the robustness of the spatial patterns. Given the complexity of

the insula and the heterogeneity of the pooled datasets, validation of the observed structures is crucial to ensure they are not artifacts of sample composition or thresholding practices. The authors should explicitly acknowledge this limitation and consider implementing, or at least proposing, validation procedures in future work.

Response 2.6. We agree that additional analyses of the spatial patterns would be valuable. To address this concern, we have conducted comprehensive validation using four independent datasets (n=608 total) that were not included in the primary analysis.

We chose one study per domain known to robustly and selectively engage that domain: thermal pain (Woo et al., 2014; n=51), monetary incentive delay task for appetitive processes (MacNiven et al., 2018; n=32), aversive image viewing (Gianaros et al., 2020; n=160), and working memory n-back task for cognitive control from the Human Connectome Project (HCP) dataset (Barch et al., 2013; n=365; restricted to genetically unrelated participants). These datasets were sourced from both the ANiC database (studies not used in main analysis) and external repositories (HCP) when domain-specific data were unavailable in ANiC. All validation data underwent identical quality control procedures and preprocessing pipelines as the primary datasets, ensuring methodological consistency.

Our validation confirmed the robustness of our spatial patterns. Domain selectivity was preserved across all zones, with each showing significant preferential activation for their target domains compared to pooled off-target domains. Pain-selective zones showed the strongest effect ($t(606)=6.98, p<0.001, d=1.02$), followed by cognitive-selective ($t(606)=6.86, p<0.001, d=0.57$), aversive-selective ($t(606)=4.32, p<0.001, d=0.40$), and appetitive-selective zones ($t(606)=2.04, p=0.042, d=0.37$). Activation in the domain-general zones were significantly above zero for all domains: pain ($t(50)=6.01, p<0.001, d=0.84$), appetitive processes ($t(31)=4.32, p<0.001, d=0.76$), aversive processes ($t(159)=15.63, p<0.001, d=1.24$), and cognitive control ($t(364)=22.79, p<0.001, d=1.19$).

The validation revealed nuanced patterns reflecting task complexity. Most notably, the Monetary Incentive Delay task (Knutson et al., 2001) used for appetitive processes validation (MacNiven et al., 2018) activated both appetitive- and cognitive-selective zones, consistent with its dual demands for reward processing and cognitive control (e.g., maintaining stimulus-reward mappings, inhibitory control during anticipatory delays).

These validation results, now detailed in the Results ("Validation with independent datasets"; pp. 9), Methods (pp. 27; "Validation with independent datasets"), and Supplementary Fig. 8 (reproduced below), demonstrate that our findings reflect generalizable functional organization rather than dataset-specific artifacts. We further discuss these findings in the Discussion, as follows, to acknowledge that some tasks naturally engage multiple functional zones based on their component processes, highlighting the iterative nature of brain-behavior mapping:

"*Relatedly, our validation findings underscore this need for refinement: while our validation confirmed domain selectivity, it also revealed that tasks can activate multiple functional zones based on their component processes. The appetitive task80 in our validation dataset elicited*

activation in both appetitive- and cognitive-selective zones, which aligns with the task's cognitive demands such as maintaining stimulus-reward mappings and sustaining inhibitory control during anticipatory delays, in addition to reward anticipation. This finding also highlights the iterative nature of brain-behavior mapping: task categorization influences the identification of neural patterns, while neural results can inform understanding of task components." (pp. 22-23)

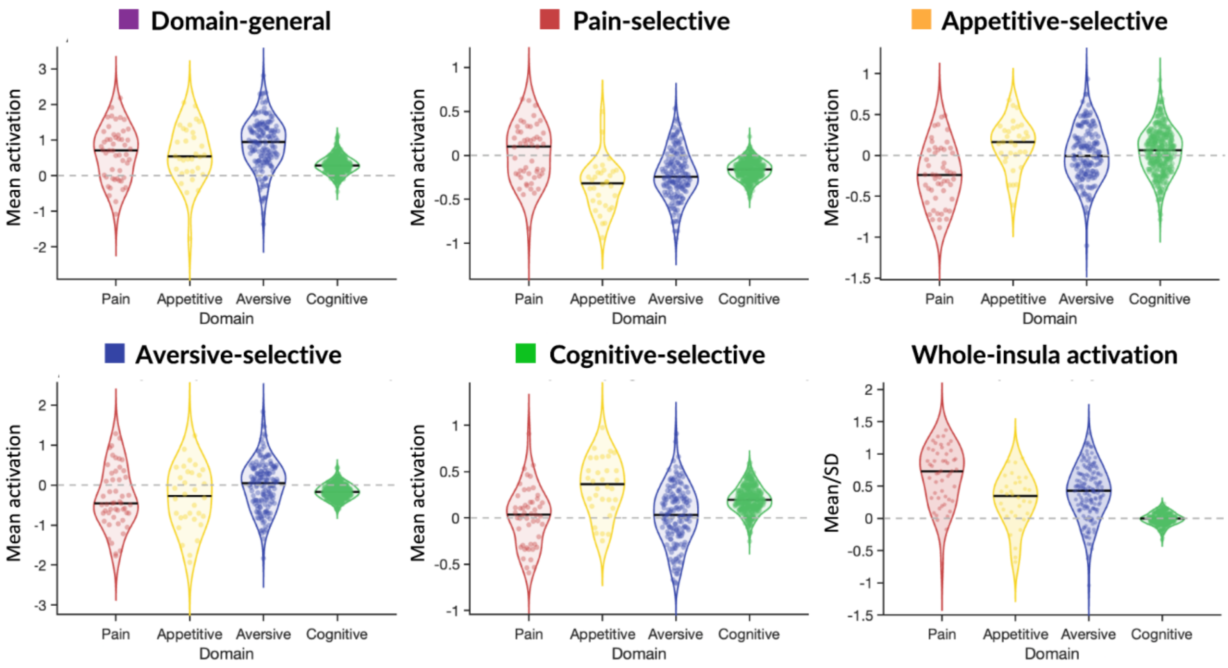

Comment 2.7. While the mega-analysis approach is valuable, the finding that the anterior insula engages across multiple domains is well established in prior literature. Although the authors propose a refined spatial structure-a core of convergence surrounded by domain-selective zones-serious concerns about anatomical precision, domain construct validity, statistical robustness, and inter-individual variability call into question the reproducibility of this fine-grained organization. As such, it is unclear whether the manuscript delivers a sufficiently novel or robust advance beyond existing models of insular function.

Response 2.7. We believe our work makes several crucial novel contributions that significantly advance the field. First, as Reviewer #1 notes, despite the insula being one of the most frequently activated regions in neuroimaging studies, a precise characterization of its functional organization has been largely missing. Our work builds upon and extends previous efforts by providing unprecedented spatial precision in mapping both convergent and domain-selective functional zones.

Regarding anterior insula specifically, while multi-domain engagement or cognitive functions is documented, we demonstrate that regions previously labeled simply as "anterior insula" actually contain both domain-general convergence zones and adjacent domain-selective zones for cognitive control. This spatial segregation reveals functional heterogeneity within an area

traditionally considered homogeneous. As discussed above in Comment 2.4, the convergence zones identified here are not isomorphic with previously identified anatomical zones and are more "patchy" and precisely specified relative to previous, more vague suggestions that the anterior insula as a whole is a convergence zone. They identify both precise areas and a novel functional gradient that can be utilized in future studies.

In addition, we identify functionally selective zones using a mega-analytic approach with full participant-level imaging data, which offers substantially improved spatial resolution compared to coordinate-based meta-analyses that rely only on peak coordinates (Costafreda, 2011; Salimi-Khorshidi et al., 2009), while providing greater statistical power than single-study approaches and generalizability (Petre et al., 2022; Radua et al., 2025). Our dataset is also more functionally diverse and comprehensive because we systematically sampled 12 functional subdomains with 3 studies per subdomain (n = 540 participants with full image information). These zones represent refined targets for use in future studies and are not duplicated by any existing findings in the literature. We expect they will provide substantial enhancements in the ability to target functional zones using, e.g., neuromodulation. For example, major efforts are underway to target drug craving and substance misuse disorders by stimulating the insula with TMS (e.g., Ibrahim et al., 2019; Malik et al., 2018; Perini et al., 2020), and we expect the targets we provide to substantially improve these efforts.

Beyond this spatial precision within anterior insula, our study advances understanding through:

- We reveal a novel hierarchical organization—discrete functional modules embedded within a gradient of convergence from periphery to center. This organizational principle, distinct from both large-sector models and smooth gradients, reconciles previously competing views of insular architecture.
- Our multi-level characterization integrating functional mapping with cytoarchitectonic boundaries and neurotransmitter receptor distributions provides an integrated model of human insula that bridges structure and function—something previously unavailable.
- Our systematic sampling ensures these patterns generalize across 36 independent studies, addressing the fundamental limitation that most insular findings come from single paradigms that may not represent broader organizational principles.

Regarding the reviewer's concerns about reproducibility, we have systematically addressed each methodological issue throughout our responses above, most notably through successful validation of our findings in independent datasets (Response 2.6), acknowledging limitations where appropriate.

We appreciate the reviewer's rigorous evaluation, which has led us to strengthen our manuscript through clearer articulation of our findings. We believe our work not only refines existing knowledge but fundamentally advances understanding by revealing organizational principles operating at multiple spatial scales and across multiple levels of analysis, from functional topography to molecular architecture. This comprehensive characterization provides the field with a precise framework for understanding how one of the brain's most functionally diverse regions achieves both specialized processing and multi-modal integration.

Reviewer #3

We thank Reviewer #3 for their positive comments as well as their thoughtful questions which have helped us improve the clarity and strength of our paper.

Comment 3.1. My main concern pertains the number of considered studies.

I see that mega-analyses are "better powered" than coordinate-based meta-analyses, so this is not a matter of statistical soundness, but rather of representation of the cognitive domains. Are 9 studies (further divided across 3 sub-domains) enough to describe something as wide as cognitive control, or pain? I think this is even more relevant as the aim here is to think in terms of selectivity. If I think of other (more or less) recent works on selectivity analyses in MRI, the idea is to test the domain of interest against the widest possible picture of alternative cases.

Related to this, I'm not very familiar with the anic database, but it is not clear to me if more studies would have been available for each domain to be selected. If this was the case, I'm wondering whether the selection of different triplets would have changed the results a lot (something that could be tested through a bootstrap approach).

Response 3.1. Thank you for raising this important issue. The sample was designed to generalize across 3 studies per subdomain, but we are also sensitive to the idea that different samples of studies could yield different findings (see Response 2.5 above).

To address concerns about robustness, as we also detailed in Response 2.6, we validated our findings with 4 independent datasets (one per domain; n=608 total participants) not included in the primary analysis. All independent studies showed activation in domain-general zones and all domain-selective zones showed significant target selectivity, demonstrating that our functional zones are robust rather than artifacts of our specific study selection. Beyond validation with independent datasets, we also tested generalization using cross-validation within our primary dataset. Our leave-one-study-per-subdomain-out cross-validated classification of domain showed robust classification of the domain identity for held-out studies (detailed in Results "Multivariate classification reveals distinct relative patterns across four functional domains", pp. 5 and Fig. 1c). These validations demonstrate that our domain representations generalize to unseen studies.

Testing different triplet selections as the reviewer suggests would provide valuable additional evidence for robustness. However, alternative studies are available for some subdomains but not for all, preventing us from maintaining the systematic and balanced sampling across all 12 subdomains. Our current dataset represents the studies we have curated to date through systematic sampling. With 9 studies per domain (36 studies and 540 participants total), our sampling exceeds similar published work on generalizable brain representations, such as Kragel et al. (2018), which included 18 total studies. The key strength of our design is the systematic sampling across different operationalizations of each domain, which identifies patterns that generalize across experimental paradigms. While being beyond the scope of this

work, as discussed in Response 2.2 and in the Discussion (pp. 22; cited below), we hope future studies could extend and test our findings as the field develops and systematic sharing of person-level activation maps becomes standard.

"*Second, we acknowledge that more comprehensive sampling of each domain would be ideal, and it would be helpful to test the generality and functional specificity in different sets of studies as person-level data become available. We have partially addressed this through validation with independent datasets (n=608), which confirmed the robustness of our findings. Beyond this, with enough studies with person-level data, future studies could even test studies within subdomains as random effects. Such comprehensive datasets with systematically coded methodological variations would also shed light on how specific methodological variables impact activation patterns. Both of these advances, however, are beyond the scope of what is available and would require systematic sharing of person-level activation maps across the field, similar to the standard reporting of study-level coordinates in tables and recent efforts to publish study-level maps. Our approach here balances comprehensive sampling with practical constraints. With 9 studies per domain across 3 subdomains (540 participants total), our sampling exceeds similar published work on generalizable brain representations, such as Kragel et al.[156] , which included 18 total studies. The key strength of our design is not the absolute number of studies but the systematic sampling across different operationalizations of each domain. This enables us to identify patterns that generalize across different experimental paradigms rather than being tied to specific implementations. Furthermore, while our study systematically sampled four functional domains, several domains important to insular function remain to be examined—e.g., chemosensation such as smell and taste[58,59], heartbeat perception[51–53,157], non-pain somatic aversive experiences like breathlessness[158] and itch[159], autonomic arousal[157], inflammation and sickness[25,48–50], nausea[160], tussis[161], and others. Addressing this limitation will require collaboration and sharing of multiple studies of sufficient size (n ≧15). We view this work as an important step toward understanding generalizable insular representations, while recognizing that future work can extend these findings with additional studies and paradigms.*" (pp. 22)

***Comment 3.2*. Following on the studies topic, why did the authors decide to include 2 experiments in the aversive domain (exp 26 and 27) being related to some extent with pain? Isn't this counterintuitive in a framework of specificity analysis?**

Also, why did the authors decide to include data from several unpublished studies? This is generally discouraged in meta-analyses.

Response 3.2. We included studies of vicarious pain for two main reasons. First, it is of theoretical interest how vicarious pain and somatic pain are related, as this bears on the nature of empathy and psychological pain and how these are encoded in the brain. Because of our interest in this topic over the past decade, we have curated several unique, high-quality datasets on rejection and vicarious pain from our lab and others. We included them in the "non-somatic aversive processes" domain because our empirical work in the past has demonstrated that vicarious pain activates neural patterns more similar to general negative affect than to

directly experienced somatic pain (Chang et al., 2015; Krishnan et al., 2016; Woo et al., 2014). However, the present study provides an empirical test of this property in the insular representation. It could have been the case that vicarious pain produced insula activation patterns similar to somatic pain, in which case the domain specificity tests would have failed and revealed that vicarious pain activates areas otherwise selective for the “pain” construct. As it turns out, the selectivity tests succeeded for both “pain” and “non-somatic aversive processes” when grouping vicarious pain with other non-somatic aversive processes. Additionally, we note that, in the construct validation literature, discriminant validity between constructs is best tested using examples of constructs that are superficially similar but thought to be part of different constructs. Thus, vicarious pain is one of the most interesting test cases in this dataset precisely because it does share some of the psychological characteristics of “pain” without satisfying the core defining property of somatic pain, a direct somatic referent (i.e., nociceptive input). Finding that vicarious pain is distinct from the “pain” construct in its activation patterns, in spite of the similar language humans use to describe them in some cases, helps refine a construct of “pain” that is aligned with neurobiological systems.

Regarding unpublished studies, it is indeed more common to include only published studies in traditional effect size meta-analyses because effect sizes from papers are the only data available. However, a number of important precedents exist for including unpublished data to avoid the “file drawer” problem and obtain a representative sample, especially from clinical trials (e.g., pharmaceutical; Kirsch et al., 2008). The main reason to limit a mega-analysis like this to published data is quality control. Here, the unpublished datasets were collected in our lab and subjected to identical preprocessing and quality control procedures as other studies, and we have validated the data quality. We included 5 unpublished datasets from 2 projects (detailed in Supplementary Table 4) primarily because certain subdomains, particularly mechanical pain and aversive sound, are underrepresented in the published literature and publicly available data is lacking. Without these data, we could not achieve our systematic sample of 36 studies balanced across 12 functional subdomains and 4 domains.

Comment 3.3. On a more methodological side, I could have got it wrong, but I’ve some reservation concerning the idea (expressed in the supplementary) that if one domain generated wider activation, normalization should be used to let other domains emerge. If it is the case that pain activates the whole insula, isn’t it exactly the kind of information that a similar paper is looking for?

Response 3.3. We appreciate this comment and the opportunity to clarify the rationale behind our normalization approach, which we have now made clearer in the revised manuscript. We are indeed interested in both the potential for global insula activation and in the relative patterns after accounting for global activation, and we report both in the manuscript.

Normalization is crucial when combining data across multiple studies to remove scaling differences introduced by scanner parameters, field strengths, and preprocessing pipelines (Botvinik-Nezer et al., 2020; Germani et al., 2025; Poldrack et al., 2017). Without normalization, these technical factors, rather than true biological differences, could dominate observed patterns. We systematically tested other normalization methods as detailed in the

supplementary methods, and found our approach to be most suitable for our research questions.

At the same time, we fully acknowledge the importance of both global insular activation patterns and more localized relative patterns. The widespread activation of the insula during pain, as the reviewer noted, indeed provides important information. Our approach therefore deliberately incorporated a two-part analysis strategy and further elaborated in our revised manuscript:

1. Analyze global insular activation, which reveals domain differences in overall engagement (particularly the robust and widespread activation during pain).
2. Analyze relative local patterns, which identifies regions preferentially engaged by specific domains.

This dual approach addresses important distinctions between widespread activation and functional specialization. Pain likely engages diffuse neuromodulatory systems (e.g., dopaminergic, noradrenergic, serotonergic) that produce widespread activation across the insula and beyond. While this global activation pattern is informative, it doesn't necessarily indicate that all activated regions are specifically processing pain information. Many regions may activate in response to increased arousal or attention demands triggered by these diffuse systems.

Our normalization approach helps distinguish between "regions that activate during pain" and "regions specialized for pain processing." This approach is common practice in representational analyses (e.g., MVPA, RSA; Haxby et al., 2014; Kriegeskorte et al., 2008) and helps identify the distinctive neural signatures of each domain beyond global effects.

We have made the following changes:

- Clarified the logic of our approach and made our two-stage analysis strategy explicit in the Introduction (p. 3) and Methods (p. 25):

  "*After data aggregation, we employed a two-part analytic strategy: (1) examining global insular activation to identify domain differences in overall engagement, and (2) analyzing relative local patterns after z-normalization to identify regions preferentially engaged by specific domains, accounting for scaling differences across studies and revealing patterns that would otherwise be masked by widespread activation.*" (pp.3)

- Moved our analysis of whole-insula activation from the Methods section to the Results section, creating a dedicated "Global Insular Activation" subsection (pp. 5) that emphasizes the finding that pain produces significantly more widespread activation across the insula compared to other domains (Fig. 1a; pp. 4; reproduced below).

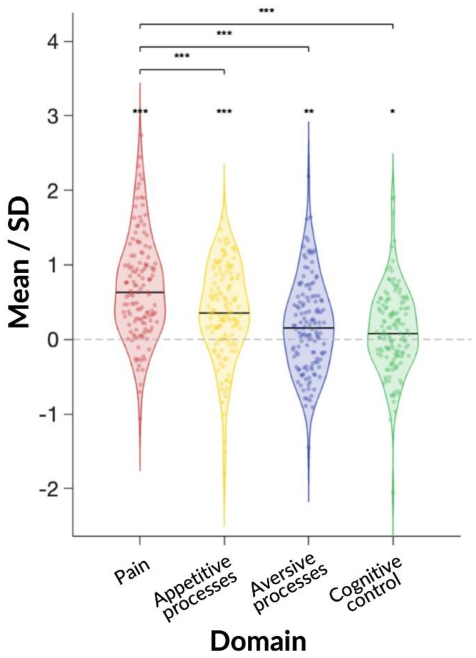

Comment 3.4. A few minor points:

I very much liked the composite nature of figure 1, but some aspects were a bit confusing to me. First, the mentioning of SVM in the title was unexpected, as I hadn't understood from the previous sections that any machine learning was performed.

Second, I'm not very sure of what map is referred in point d of the caption. Is it that appearing in panel c? Or did the authors refer to the diagram in panel d, that in this case I would not define a map?

As commented on figure 1, I would consider to introduce SVM somewhere before the results section, since the methodological framework seems rather to be on mega-analyses and Bayesian statistics only.

Response 3.4. We have added an introduction to multivariate classification in the Introduction section to clarify our analytical approach as follows.

"*We then validated that these domains have separable insular activity patterns that generalize across studies using multivariate pattern analysis (leave-one-study-per-subdomain-out cross-validated support vector machines (SVM))*" (pp. 3)

We also corrected the placement of the caption text "*Example map shows voxel-wise BFs for pain (red: stronger evidence for effect; blue: stronger evidence for no effect)*" from panel (d) to panel (c) where the BF map is actually displayed.

Comment 3.5. At first read, it is not clear why 33.17% for aversive processes is not above-chance, that is previously set to 25%. I guess it depends on the p, but this could be maybe made more straightforward for readers.

Response 3.5. We clarified in the Results that aversive processes showed accuracy (33.17%) that was “numerically above chance but did not reach statistical significance” (pp. 6) making explicit why this numerically higher value is not considered significant.

Comment 3.6. I’m not a native English speaker, but the term “coherent” in the sentence "Overall, these findings indicate that three of the four domains show coherent insular representations" makes me think that the three domains are similar among them (while the details in brackets clarifies it is not so). I would suggest to consider to change “coherent” with a different synonym.

Response 3.6. We replaced “coherent” with “separable” in the Results as follows:

“*At the pattern level, three of the four domains show separable insular representations that generalize across studies and tasks.*” (pp. 6)

Conclusion

We are grateful for the reviewers’ valuable feedback, which has helped us significantly improve our manuscript. We have addressed the issues raised through additional analyses, expanded discussion, and clarification of our methods and findings. We believe these changes have considerably strengthened the paper. Thank you.

References

Barch, D. M., Burgess, G. C., Harms, M. P., Petersen, S. E., Schlaggar, B. L., Corbetta, M., Glasser, M. F., Curtiss, S., Dixit, S., Feldt, C., Nolan, D., Bryant, E., Hartley, T., Footer, O., Bjork, J. M., Poldrack, R., Smith, S., Johansen-Berg, H., Snyder, A. Z., & Van Essen, D. C. (2013). Function in the human connectome: Task-fMRI and individual differences in behavior. *NeuroImage*, *80*, 169–189. https://doi.org/10.1016/j.neuroimage.2013.05.033

Botvinik-Nezer, R., Holzmeister, F., Camerer, C. F., Dreber, A., Huber, J., Johannesson, M., Kirchler, M., Iwanir, R., Mumford, J. A., Adcock, R. A., Avesani, P., Baczkowski, B. M., Bajracharya, A., Bakst, L., Ball, S., Barilari, M., Bault, N., Beaton, D., Beitner, J., … Schonberg, T. (2020). Variability in the analysis of a single neuroimaging dataset by many teams. *Nature*, *582*(7810), Article 7810. https://doi.org/10.1038/s41586-020-2314-9

Burton, H., Fabri, M., & Alloway, K. (1995). Cortical areas within the lateral sulcus connected to cutaneous representations in areas 3b and 1: A revised interpretation of the second somatosensory area in macaque monkeys. *Journal of Comparative Neurology*, *355*(4), 539–562. https://doi.org/10.1002/cne.903550405

Čeko, M., Kragel, P. A., Woo, C.-W., López-Solà, M., & Wager, T. D. (2022). Common and stimulus-type-specific brain representations of negative affect. *Nature Neuroscience*, *25*(6), 760–770. https://doi.org/10.1038/s41593-022-01082-w

Chang, L. J., Gianaros, P. J., Manuck, S. B., Krishnan, A., & Wager, T. D. (2015). A Sensitive and Specific Neural Signature for Picture-Induced Negative Affect. *PLOS Biology*, *13*(6), e1002180. https://doi.org/10.1371/journal.pbio.1002180

Costafreda, S. G. (2011). Meta-Analysis, Mega-Analysis, and Task Analysis in fMRI Research. *Philosophy, Psychiatry, & Psychology*, *18*(4), 275–277. https://doi.org/10.1353/ppp.2011.0049

Craig, A. D. (2002). How do you feel? Interoception: the sense of the physiological condition of the body. *Nature Reviews Neuroscience*, *3*(8), Article 8. https://doi.org/10.1038/nrn894

Craig, A. D. (2009). How do you feel — now? The anterior insula and human awareness. *Nature Reviews Neuroscience*, *10*(1), Article 1. https://doi.org/10.1038/nrn2555

Damasio, A. R. (1989). The Brain Binds Entities and Events by Multiregional Activation from Convergence Zones. *Neural Computation*, *1*(1), 123–132. https://doi.org/10.1162/neco.1989.1.1.123

Dworetsky, A., Seitzman, B. A., Adeyemo, B., Nielsen, A. N., Hatoum, A. S., Smith, D. M., Nichols, T. E., Neta, M., Petersen, S. E., & Gratton, C. (2024). Two common and distinct forms of variation in human functional brain networks. *Nature Neuroscience*, *27*(6), 1187–1198. https://doi.org/10.1038/s41593-024-01618-2

Evrard, H. C. (2019). The Organization of the Primate Insular Cortex. *Frontiers in Neuroanatomy*, *13*. https://www.frontiersin.org/articles/10.3389/fnana.2019.00043

Evrard, H. C., Logothetis, N. K., & (Bud) Craig, A. d. (2014). Modular architectonic organization of the insula in the macaque monkey. *Journal of Comparative Neurology*, *522*(1), 64–97. https://doi.org/10.1002/cne.23436

Garcia-Larrea, L., Perchet, C., Creac'h, C., Convers, P., Peyron, R., Laurent, B., Mauguière, F., & Magnin, M. (2010). Operculo-insular pain (parasylvian pain): A distinct central pain syndrome. *Brain*, *133*(9), 2528–2539. https://doi.org/10.1093/brain/awq220

Germani, E., Rolland, X., Maurel, P., & Maumet, C. (2025). On the validity of fMRI mega-analyses using data processed with different pipelines. *Imaging Neuroscience*, *3*, imag_a_00522. https://doi.org/10.1162/imag_a_00522

Gianaros, P. J., Kraynak, T. E., Kuan, D. C.-H., Gross, J. J., McRae, K., Hariri, A. R., Manuck, S. B., Rasero, J., & Verstynen, T. D. (2020). Affective brain patterns as multivariate neural correlates of cardiovascular disease risk. *Social Cognitive and Affective Neuroscience*, *15*(10), 1034–1045. https://doi.org/10.1093/scan/nsaa050

Glasser, M. F., Coalson, T. S., Robinson, E. C., Hacker, C. D., Harwell, J., Yacoub, E., Ugurbil, K., Andersson, J., Beckmann, C. F., Jenkinson, M., Smith, S. M., & Van Essen, D. C. (2016). A multi-modal parcellation of human cerebral cortex. *Nature*, *536*(7615), 171–178. https://doi.org/10.1038/nature18933

Grodzinsky, Y., Deschamps, I., Pieperhoff, P., Iannilli, F., Agmon, G., Loewenstein, Y., & Amunts, K. (2020). Logical negation mapped onto the brain. *Brain Structure and Function*, *225*(1), 19–31. https://doi.org/10.1007/s00429-019-01975-w

Haxby, J. V., Connolly, A. C., & Guntupalli, J. S. (2014). Decoding Neural Representational Spaces Using Multivariate Pattern Analysis. *Annual Review of Neuroscience*, *37*(1), 435–456. https://doi.org/10.1146/annurev-neuro-062012-170325

Hein, M., Mohlberg, H., Bludau, S., Quabs, J., & Amunts, K. (2021a). *Probabilistic cytoarchitectonic map of Area Ia2 (Insula) (v4.0)* [Dataset]. EBRAINS. https://doi.org/10.25493/BMNK-B8F

Hein, M., Mohlberg, H., Bludau, S., Quabs, J., & Amunts, K. (2021b). *Probabilistic cytoarchitectonic map of Area Ia3 (Insula) (v4.0)* [Dataset]. EBRAINS. https://doi.org/10.25493/QS00-PJ9

Hein, M., Mohlberg, H., Bludau, S., Quabs, J., & Amunts, K. (2021c). *Probabilistic cytoarchitectonic map of Area Id9 (Insula) (v4.0)* [Dataset]. EBRAINS. https://doi.org/10.25493/JMCR-ZNQ

Ibrahim, C., Rubin-Kahana, D. S., Pushparaj, A., Musiol, M., Blumberger, D. M., Daskalakis, Z. J., Zangen, A., & Le Foll, B. (2019). The Insula: A Brain Stimulation Target for the Treatment of Addiction. *Frontiers in Pharmacology*, *10*. https://www.frontiersin.org/articles/10.3389/fphar.2019.00720

Kirsch, I., Deacon, B. J., Huedo-Medina, T. B., Scoboria, A., Moore, T. J., & Johnson, B. T. (2008). Initial Severity and Antidepressant Benefits: A Meta-Analysis of Data Submitted to the Food and Drug Administration. *PLOS Medicine*, *5*(2), e45. https://doi.org/10.1371/journal.pmed.0050045

Knutson, B., Adams, C. M., Fong, G. W., & Hommer, D. (2001). Anticipation of Increasing Monetary Reward Selectively Recruits Nucleus Accumbens. *The Journal of Neuroscience*, *21*(16), RC159–RC159. https://doi.org/10.1523/JNEUROSCI.21-16-j0002.2001

Kriegeskorte, N., Mur, M., & Bandettini, P. (2008). Representational similarity analysis—Connecting the branches of systems neuroscience. *Frontiers in Systems Neuroscience*, *2*. https://www.frontiersin.org/article/10.3389/neuro.06.004.2008

Krishnan, A., Woo, C.-W., Chang, L. J., Ruzic, L., Gu, X., López-Solà, M., Jackson, P. L., Pujol, J., Fan, J., & Wager, T. D. (2016). Somatic and vicarious pain are represented by dissociable multivariate brain patterns. *eLife*, *5*, e15166. https://doi.org/10.7554/eLife.15166

Krockenberger, M. S., Saleh-Mattesich, T. O., & Evrard, H. C. (2023). Cytoarchitectonic and connection stripes in the dysgranular insular cortex in the macaque monkey. *Journal of Comparative Neurology*, *531*(18), 2019–2043. https://doi.org/10.1002/cne.25571

Kurth, F., Eickhoff, S., Schleicher, A., Hoemke, L., Zilles, K., & Amunts, K. (2010). Cytoarchitecture and probabilistic maps of the human posterior insular cortex. *Cerebral Cortex*. https://doi.org/10.1093/CERCOR/BHP208

Kurth, F., Zilles, K., Fox, P. T., Laird, A. R., & Eickhoff, S. B. (2010). A link between the systems: Functional differentiation and integration within the human insula revealed by meta-analysis. *Brain Structure and Function*, *214*(5), 519–534. https://doi.org/10.1007/s00429-010-0255-z

MacNiven, K. H., Jensen, E. L. S., Borg, N., Padula, C. B., Humphreys, K., & Knutson, B. (2018). Association of Neural Responses to Drug Cues With Subsequent Relapse to Stimulant Use. *JAMA Network Open*, *1*(8), e186466. https://doi.org/10.1001/jamanetworkopen.2018.6466

Malik, S., Jacobs, M., Cho, S.-S., Boileau, I., Blumberger, D., Heilig, M., Wilson, A., Daskalakis, Z. J., Strafella, A. P., Zangen, A., & Le Foll, B. (2018). Deep TMS of the insula using the H-coil modulates dopamine release: A crossover [11C] PHNO-PET pilot trial in healthy humans. *Brain Imaging and Behavior*, *12*(5), 1306–1317. https://doi.org/10.1007/s11682-017-9800-1

Mazzola, L., Isnard, J., & Mauguière, F. (2006). Somatosensory and Pain Responses to Stimulation of the Second Somatosensory Area (SII) in Humans. A Comparison with SI and Insular Responses. *Cerebral Cortex*, *16*(7), 960–968. https://doi.org/10.1093/cercor/bhj038

Mesulam, M.-M., & Mufson, E. J. (1982). Insula of the old world monkey. III: Efferent cortical output and comments on function. *Journal of Comparative Neurology*, *212*(1), 38–52. https://doi.org/10.1002/cne.902120104

Oppenheimer, S., & Cechetto, D. (2016). The Insular Cortex and the Regulation of Cardiac Function. *Comprehensive Physiology*, *6*(2), 1081–1133. https://doi.org/10.1002/j.2040-4603.2016.tb00691.x

Oppenheimer, S. M., Gelb, A., Girvin, J. P., & Hachinski, V. C. (1992). Cardiovascular effects of human insular cortex stimulation. *Neurology*, *42*(9), 1727–1727. https://doi.org/10.1212/WNL.42.9.1727

Perini, I., Kämpe, R., Arlestig, T., Karlsson, H., Löfberg, A., Pietrzak, M., Zangen, A., & Heilig, M. (2020). Repetitive transcranial magnetic stimulation targeting the insular cortex for reduction of heavy drinking in treatment-seeking alcohol-dependent subjects: A randomized controlled trial. *Neuropsychopharmacology*, *45*(5), 842–850. https://doi.org/10.1038/s41386-019-0565-7

Petre, B., Kragel, P., Atlas, L. Y., Geuter, S., Jepma, M., Koban, L., Krishnan, A., Lopez-Sola, M., Losin, E. A. R., Roy, M., Woo, C.-W., & Wager, T. D. (2022). A multistudy analysis reveals that evoked pain intensity representation is distributed across brain systems. *PLOS Biology*, *20*(5), e3001620. https://doi.org/10.1371/journal.pbio.3001620

Poldrack, R. A., Baker, C. I., Durnez, J., Gorgolewski, K. J., Matthews, P. M., Munafò, M. R., Nichols, T. E., Poline, J.-B., Vul, E., & Yarkoni, T. (2017). Scanning the horizon: Towards transparent and reproducible neuroimaging research. *Nature Reviews Neuroscience*, *18*(2), 115–126. https://doi.org/10.1038/nrn.2016.167

Quabs, J., Bittner, N., & Caspers, S. (2025). Structural Connectivity Differences Reflect Microstructural Heterogeneity of the Human Insular Cortex. *Human Brain Mapping*, *46*(8), e70231. https://doi.org/10.1002/hbm.70231

Quabs, J., Caspers, S., Schöne, C., Mohlberg, H., Bludau, S., Dickscheid, T., & Amunts, K. (2022). Cytoarchitecture, probability maps and segregation of the human insula. *NeuroImage*, *260*, 119453. https://doi.org/10.1016/j.neuroimage.2022.119453

Radua, J., Savage, H. S., Vilajosana, E., Jamieson, A., Abler, B., Åhs, F., Beckers, T., Cardoner, N., Cisler, J. M., Diniz, J. B., Bach, D. R., Elsenbruch, S., Greening, S. G., Holt, D. J., Kaczkurkin, A. N., Keil, A., Kindt, M., Koch, K., LaBar, K. S., … Fullana, M. A. (2025). Neural correlates of human fear conditioning and sources of variability in 2199 individuals. *Nature Communications*, *16*(1), 7869. https://doi.org/10.1038/s41467-025-63078-x

Sabat, M., de Dampierre, C., & Tallon-Baudry, C. (2025). Evidence for domain-general arousal from semantic and neuroimaging meta-analyses reconciles opposing views on arousal. *Proceedings of the National Academy of Sciences*, *122*(6), e2413808122. https://doi.org/10.1073/pnas.2413808122

Salimi-Khorshidi, G., Smith, S. M., Keltner, J. R., Wager, T. D., & Nichols, T. E. (2009). Meta-analysis of neuroimaging data: A comparison of image-based and coordinate-based pooling of studies. *NeuroImage*, *45*(3), 810–823. https://doi.org/10.1016/j.neuroimage.2008.12.039

Segerdahl, A. R., Mezue, M., Okell, T. W., Farrar, J. T., & Tracey, I. (2015). The dorsal posterior insula subserves a fundamental role in human pain. *Nature Neuroscience*, *18*(4), 499–500. https://doi.org/10.1038/nn.3969

Selemon, L. D., & Goldman-Rakic, P. S. (1988). Common cortical and subcortical targets of the dorsolateral prefrontal and posterior parietal cortices in the rhesus monkey: Evidence for a distributed neural network subserving spatially guided behavior. *Journal of Neuroscience*, *8*(11), 4049–4068. https://doi.org/10.1523/JNEUROSCI.08-11-04049.1988

Simmons, W. K., & Barsalou, L. W. (2003). The Similarity-in-Topography Principle: Reconciling Theories of Conceptual Deficits. *Cognitive Neuropsychology*, *20*(3–6), 451. https://doi.org/10.1080/02643290342000032

Woo, C.-W., Koban, L., Kross, E., Lindquist, M. A., Banich, M. T., Ruzic, L., Andrews-Hanna, J. R., & Wager, T. D. (2014). Separate neural representations for physical pain and social rejection. *Nature Communications*, *5*(1), Article 1. https://doi.org/10.1038/ncomms6380
